# Phosphine-Catalyzed γ′-Carbon 1,6-Conjugate Addition of α-Succinimide Substituted Allenoates with *Para*-Quinone Methides: Synthesis of 4-Diarylmethylated 3,4-Disubstituted Maleimides

**DOI:** 10.3390/molecules29112593

**Published:** 2024-05-31

**Authors:** Zhenzhen Gao, Xiaoming Zhou, Dandan Liu, Baoshen Nie, Hanchong Lu, Xiaotong Chen, Jiahui Wu, Lei Li, Xuekun Wang

**Affiliations:** State Key Laboratory for Macromolecule Drugs and Large-Scale Manufacturing, School of Pharmaceutical Sciences, Liaocheng University, Liaocheng 252059, China; 17861822319@163.com (X.Z.); 17861831787@163.com (D.L.); 15806805721@163.com (B.N.); lhclhlhappyend@163.com (H.L.); 15621508513@163.com (X.C.); 18306595180@163.com (J.W.)

**Keywords:** phosphine-catalyzed, allenoates, 1,6-conjugate addition, *para*-quinone methides

## Abstract

In this paper, an interesting γ′-carbon 1,6-conjugate addition for phosphine-catalyzed α-succinimide substituted allenoates has been disclosed. A wide array of substrates was found to participate in the reaction, resulting in the production of diverse 4-diarylmethylated 3,4-disubstituted maleimides with satisfactory to outstanding yields. Furthermore, a plausible mechanism for the reaction was proposed by the investigators.

## 1. Introduction

**Scheme 1:**
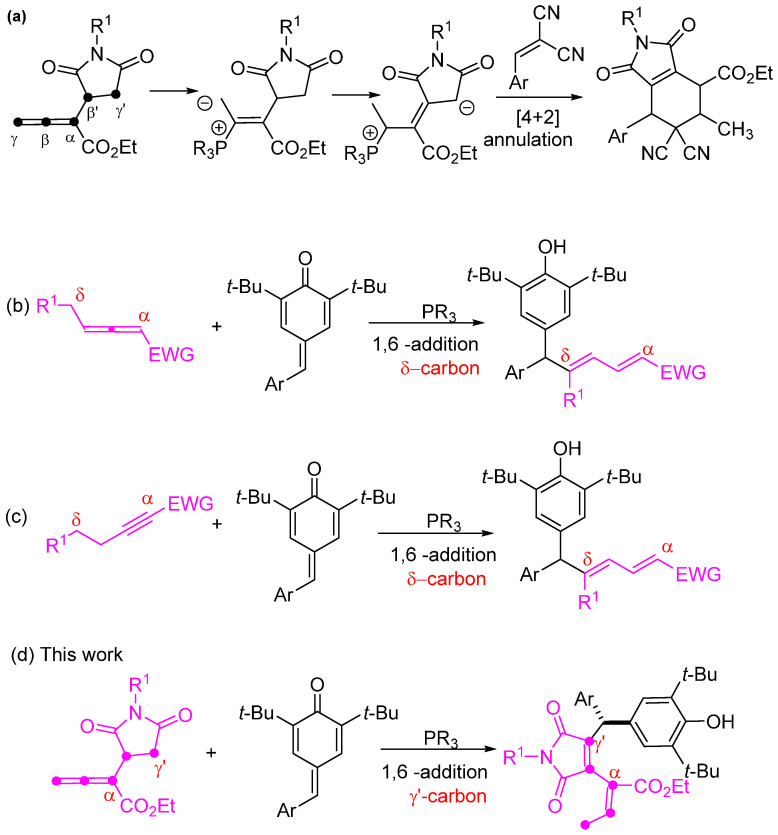
Reaction mode of α-succinimide substituted allenoates and overview of 1, 6-conjugated addition of phosphine catalysis. (**a**) Phosphine-catalyzed α-succinimide substituted allenoate [4 + 2] annulation reaction with 1,1-dicyanoalkenes; (**b**) Phosphine-catalyzed allenoate isomerization/1,6-conjugate addition reaction with *para*-quinone methides; (**c**) Phosphine-catalyzed 1,6-conjugate addition of an alkynoate with *para*-quinone methides; (**d**) Phosphine-catalyzed γ′-carbon 1,6-addition of α-succinimide substituted allenoates to *para*-quinone methides.

Over the past two decades, there has been a notable growth in phosphine catalysis within organic synthesis. This expansion is attributed to the versatile nature of phosphine, attracting attention from numerous research groups [1,2,3,4,5,6,7,8,9,10,11,12]. Since the pioneering work of Kwon’s group in 2003, where they introduced α-substituted allenoates for phosphine-catalyzed [4 + 2] annulation [13], the potential of these allenoates in phosphine catalysis has been extensively explored. While various electrophilic partners have been employed in phosphine-catalyzed nucleophilic additions or annulations with α-substituted allenoates [14,15,16,17,18,19,20,21,22,23,24,25,26,27,28,29,30,31,32,33], there remains value in investigating novel α-substituted allenoates with diverse structures. Such research endeavors hold promise for revealing new reaction pathways and enriching synthetic routes for a wide array of functional molecules. Very recently, our group was the first to report a series of new α-succinimide substituted allenoates, which can undergo a [4 + 2] annulation reaction with 1,1-dicyanoalkenes under phosphine catalysis, involving the γ′-carbon of the allenoates (Scheme 1a) [34]. But there have been very few works related to the γ′-carbon addition of allenoates under phosphine catalysis [35,36]. Therefore, the potential of α-succinimide substituted allenoates in nucleophilic phosphine catalysis remains underexplored. Therefore, continuous exploration of the addition reactions involving this allenoate is highly recommended.

In recent years, *para*-quinone methides have emerged as versatile building blocks for 1,6-conjugate addition reactions, facilitating the synthesis of functionalized diarylmethane derivatives [37,38,39,40,41,42,43,44,45,46,47]. Catalytic reactions play an important role in many transformations of *para*-quinone methides, and some have been established through the use of catalysts such as Lewis acids, Brønsted acids, bases, transition metals, and N-heterocyclic carbenes [38,48]. In a recent investigation, these 1,6-addition reactions to *para*-quinone methides taking place under phosphine catalysis were already reported, achieving the phosphine-catalyzed direct dienylation with *para*-quinone methides. Xu et al. documented a chiral phosphine-catalyzed allenoate isomerization/1,6-conjugate addition cascade reaction with *para*-quinone methides. The reaction exclusively proceeds via the δ-carbon under phosphine catalysis (Scheme 1b) [49]. Another study by Wang et al. in 2021 revealed a phosphine-catalyzed protocol for the 1,6-conjugate addition of an alkynoate via the δ-carbon with *para*-quinone methides (Scheme 1c) [50]. To the best of our knowledge, γ′-carbon 1,6-conjugate addition with *para*-quinone methides catalyzed by phosphine has never been reported before. 

Heterocyclic diarylmethane molecules have exhibited a wide range of biological applications [51,52,53,54,55,56,57,58,59]. As part of our ongoing efforts to develop new methodologies for synthesizing functionalized organic compounds under phosphine catalysis, we introduce an innovative and highly regioselective synthesis of 4-diarylmethylated 3,4-disubstituted maleimides. Herein, we report the first example of a phosphine catalyzed γ′-carbon 1,6-addition of α-succinimide substituted allenoates to *para*-quinone methides (Scheme 1d). 

## 2. Results and Discussion

This investigation commenced by examining the reaction between α-succinimide allenoate **2a** and *para*-quinone methide (*p*-QM) **1a**. A catalyst, MePPh_2_, was employed at 20 mol % with DCM as the solvent. The desired product **3aa** was obtained with a yield of 34% (Table 1, entry 1). Various phosphines were also tested for the cascade remote 1, 6-addition reactions. However, no enhancement in reactivity was observed with the phosphines EtPPh_2_ and PrPPh_2_ (Table 1, entries 2, 3). Fortunately, when the reaction was attempted with Me_2_PPh and PMe_3_, smooth conversion was achieved, yielding the 1,6-conjugate addition product **3aa** with yields of 67% and 92%, respectively (Table 1, entries 4, 5). It is noteworthy that the presence of PCy_3_ or PBu_3_, with their bulky substitution groups, resulted in decreased yields (Table 1, entries 6, 7). Considering the optimal catalyst, PMe_3_, we further optimized the reactions, using different solvents. Solvent screening revealed that DCE yielded similar results to DCM (Table 1, entry 8), while toluene, THF, and EtOAc exhibited low reactivity as solvents (Table 1, entries 9–11). Conversely, CH_3_CN and CH_3_OH resulted in inferior outcomes (Table 1, entries 12, 13). By reducing the loading of PMe_3_ to 10 mol % and 5 mol %, the yields of **3aa** decreased to 85% and 75%, respectively (Table 1, entries 14, 15). Therefore, the optimal reaction conditions were determined to be DCM as the solvent, room temperature, and 20 mol% of PMe_3_ as the catalyst.

**Scheme 2:**
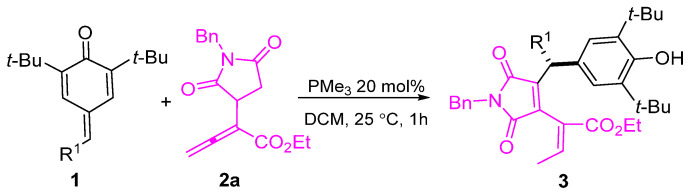

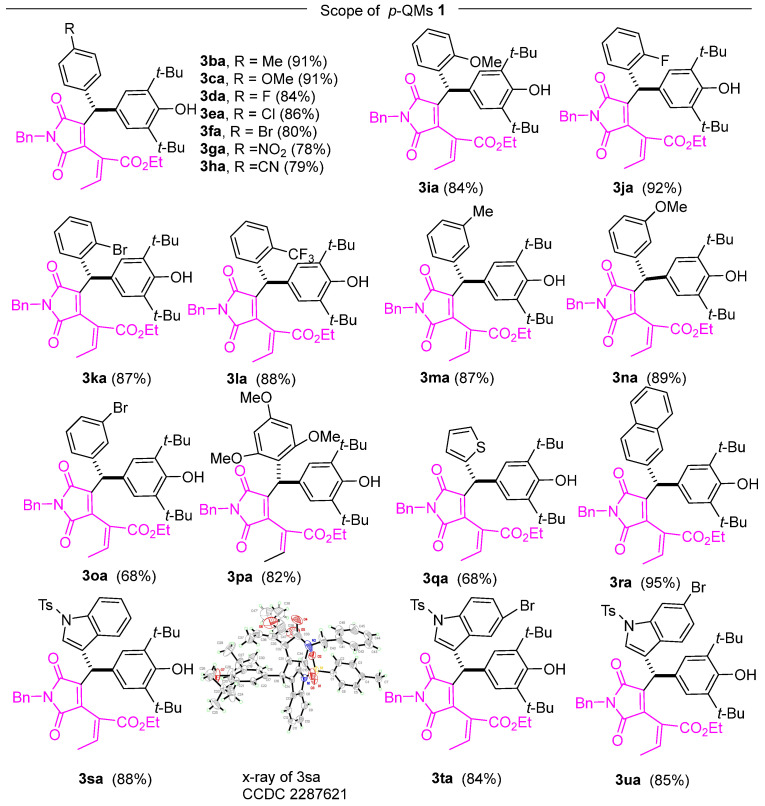
Substrate scope of the *p*-QMs.

Following the determination of optimal conditions for 1,6-conjugate addition, we investigated the substrate scope of this reaction. In Scheme 1, we explored the influence of various functional groups on the benzene ring of *p*-QMs. Under the optimized conditions, it was observed that the *p*-QMs carrying various electronically diverse functional groups at the *para*-position of the phenyl ring facilitated the formation of conjugate adducts with yields ranging from good to excellent. Smooth conjugate addition was achieved with electron-rich methyl- and methoxy-substituted aryl quinone methides, resulting in excellent yields of the adducts (Scheme 2, **3ba**, **3ca**). Similarly, the reactions effectively yielded the corresponding products in good yields (Scheme 2, **3da**–**3ha**) when employing arenes containing electron-deficient functional groups such as halogens, nitro groups, and cyano groups. Additionally, satisfactory performance was demonstrated in the reaction with *p*-QMs containing an ortho-substituted aryl moiety (Scheme 2, **3ia**–**3la**). The applicability of the reaction was expanded to include meta-substituted aryl quinone methides (Scheme 2, **3ma**–**3oa**). Furthermore, trisubstituted aryl quinone methides were investigated, and the conjugate addition yielded the desired in good yields (Scheme 2, **3pa**). Moreover, the reaction was employed with *p*-QMs derived from β-naphthaldehyde, resulting in enhanced product yields (Scheme 2, **3ra**). The reaction also showed success with the *p*-QM substrates generated from thiophene-2-carboxaldehyde and indole-3-carboxaldehyde (Scheme 2, **3qa**, **3sa**–**3ua**). The structure of **3sa** was confirmed via X-ray analysis (CCDC 2287621) [60]. These data are accessible free of charge from the Cambridge Crystallographic Data Centre via http://www.ccdc.cam.ac.uk/data_request/cif. 9 August 2023. (DOI: 10.5517/ccdc.csd.cc2gsg7z). For further details regarding the crystal structure of **3sa**, please refer to the Supplementary Materials.

**Scheme 3:**
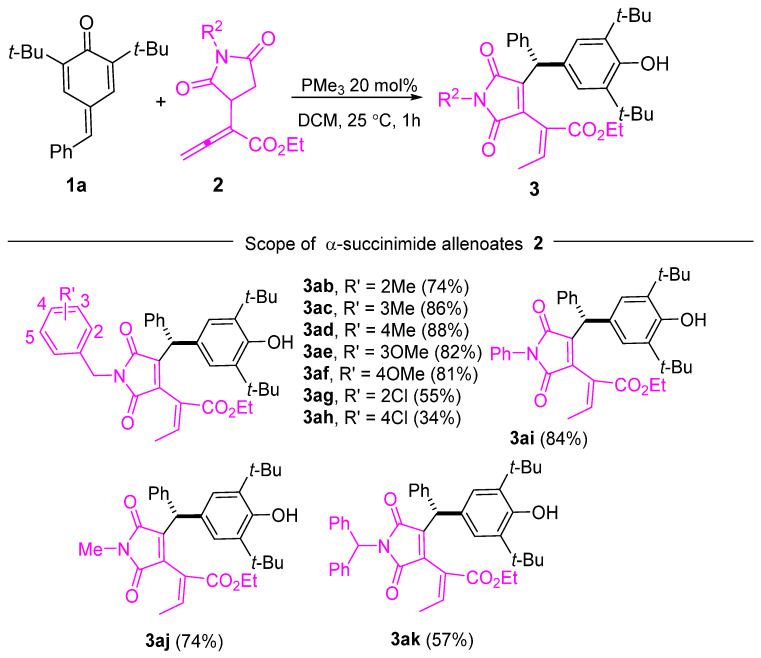
Substrate scope of the α-succinimide allenoates.

In addition to *p*-QMs, we explored the substrate scope of α-succinimide allenoates with various R^2^ groups using **1a** as the coupling partner (Scheme 3). We were pleased to observe that the reactivity of the substrates remained unaffected when electron-donating substituents were introduced into the benzyl phenyl ring, resulting in yields of 74–81% (Scheme 3, **3ab**–**3af**). However, the yields slightly decreased to 55% and 34% for **3ag** and **3ah**, respectively, when R’ groups with electron-withdrawing properties were employed. Encouragingly, the reaction displayed good tolerance toward the phenyl group, as evidenced by the successful formation of the corresponding products in 84% yield (Scheme 3, **3ai**). Additionally, when R^2^ was a methyl group or a diphenyl-substituted methyl group, the products were obtained with respective yields of 74% (Scheme 2, **3aj**) and 57% (Scheme 3, **3ak**).

**Scheme 4:**
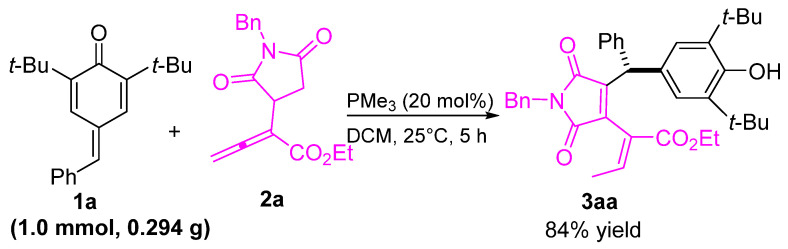
The scale-up reaction.

To demonstrate the applicability of our method, we conducted a scale-up reaction. As depicted in Scheme 4, the reaction between **1a** and allenoate **2a** proceeded smoothly under optimal conditions, yielding desired product **3aa** at the gram scale without any noticeable loss in yield. 

**Scheme 5:**
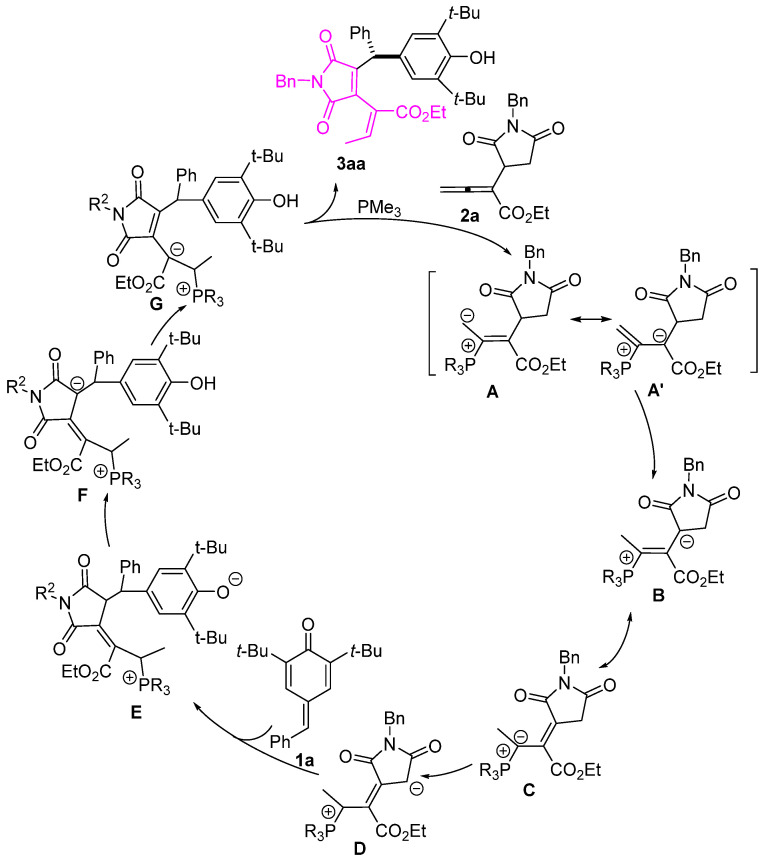
Proposed mechanism. The letter numbers A-G indicate possible intermediate structures in the plausible reaction mechanism.

A plausible reaction mechanism is proposed in Scheme 5. This addition reaction begins with the nucleophilic addition of a phosphine to the allenoates **2a**, forming zwitterionic intermediates (**A**⟷**A**′). Subsequent proton transfer leads to intermediate **B**, followed by the formation of intermediate **D** through isomerization and proton transfer. Intermediate **D** then attacks *p*-QM **1a**, forming intermediate **E**. Subsequently, intermediate **E** undergoes a series of H-transfers to form intermediate **G**. The elimination of PR_3_ from **G** produces product **3aa**, which regenerates PR_3_ to complete the catalytic cycle. 

## 3. Materials and Methods

All chemical reactions were carried out under an argon atmosphere using oven-dried glassware. This setup included magnetic stirring to ensure proper mixing of reagents. Unless otherwise stated, all chemicals were purchased from commercial suppliers and used as received without any additional purification. However, all solvents used in the reactions were purified and dried prior to use, according to standard laboratory procedures. The progress of the chemical reactions was monitored by thin-layer chromatography (TLC) on glass plates precoated with silica gel, and fluorescence quenching with UV light at a wavelength of 254 nm was used to visualize the chromatograms. Any necessary purification of the products was carried out by flash column chromatography using the Qingdao Haiyang flash silica gel (Qingdao, China) with a particle size range of 100–200 mesh. Nuclear magnetic resonance (NMR) spectra, both proton (^1^H) and carbon (^13^C), were recorded in deuterated CDCl_3_ or DMSO-*d*6 using the 500 MHz NMR spectrometer. The melting points of the compounds were determined using an X-4 digital micro-melting point apparatus from Shanghai Jingke (Shanghai, China) to ensure accuracy. An Agilent instrument using electrospray ionization mass spectrometry (ESI-MS) (Campus Drive Stanford, CA, USA) was used to obtain accurate mass measurements. In addition, X-ray crystallographic data were collected using a Bruker D8 VENTURE instrument (Billerica, Germany) to provide detailed structural information on the synthesized compounds. Characterization data of compounds, NMR spectra of compounds and crystallographic data for product 3sa, See Supplementary Materials.

### 3.1. General Procedure for the Synthesis of Para-Quinone Methides **1**

Aldehyde (10 mmol) was added to a solution of the phenol (10 mmol) in toluene (40 mL). The reaction mixture was heated in a Dean–Stark apparatus to reflux. Piperidine (20 mmol) was added dropwise over 1 h, and heating continued until all the starting material had been consumed. After the mixture had cooled just below the boiling point of toluene (100 °C), acetic anhydride (20 mmol) was added. The solution was stirred for 15 min. The residue was extracted three times with dichloromethane. The combined organic layers were sequentially washed with water and brine, dried over magnesium sulfate, filtered, and concentrated. The crude product was purified by flash column chromatography on silica gel to afford the corresponding product **1**.

### 3.2. General Procedure for the Synthesis of α-Succinimide Substituted Allenoates **2**

Maleimide (0.15 mmol), allene (0.30 mmol), DABCO (0.030 mmol), and 1,4-dioxane (1.0 mL) were added to a Schlenk tube. The reaction mixture was stirred at room temperature for 3 h, the solvent was then removed under reduced pressure, and the residue was purified by flash column chromatography (PE/EA = 4/1~2/1) to afford products **2**.

### 3.3. General Procedure for the Phosphine-Catalyzed Direct 1,6-Conjugate Addition

Under argon atmosphere, 1 mL of DCM was added to a mixture of *para*-quinone methide **1** (0.10 mmol), α-succinimide substituted allenoate **2** (0.12 mmol) and catalyst PMe_3_ (20 mol%, 0.02 mmol) in a Schlenk tube at room temperature. The resulting mixture was stirred until the starting material was completely consumed (monitored by TLC) and then concentrated to dryness. The residue was purified through flash column chromatography (PE/EtOAc = 8:1) to afford corresponding cycloaddition products **3**.

## 4. Conclusions

In conclusion, we have introduced a novel method for synthesizing functionalized 4-diarylmethylated 3,4-disubstituted maleimides in satisfactory yields. This method involves a phosphine-catalyzed 1,6-conjugated addition reaction between α-succinimide substituted allenoates and *p*-QMs. Considering the extensive research on the biological activity of natural products and industrially useful compounds containing the 3,4-disubstituted maleimide moiety, our methodology presents a new and efficient protocol for their synthesis.

## Figures and Tables

**Table 1:** Reaction yields under different reaction conditions.

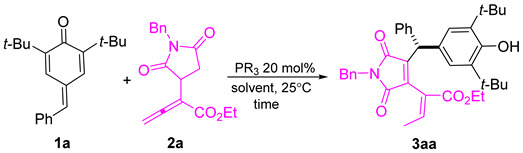				
Entry ^a^	PR_3_	Solvent	Time/h	Yield/% ^b^
1	MePPh_2_	DCM	1	34
2	EtPPh_2_	DCM	1	Trace
3	PrPPh_2_	DCM	1	Trace
4	Me_2_PPh	DCM	1	67
5	PMe_3_	DCM	1	92
6	PBu_3_	DCM	1	40
7	PCy_3_	DCM	1	20
8	PMe_3_	DCE	2	90
9	PMe_3_	Toluene	1	84
10	PMe_3_	THF	1	73
11	PMe_3_	EtOAc	1	61
12	PMe_3_	MeOH	1	19
13	PMe_3_	CH_3_CN	1	NR
14 ^c^	PMe_3_	DCM	5	85
15 ^d^	PMe_3_	DCM	10	75

^a^ Unless otherwise indicated, all reactions were carried out at room temperature using 0.10 mmol of **1a** and 0.12 mmol of **2a** in a solvent containing 20 mol% of the catalyst. ^b^ Isolated yield. ^c^ PMe_3_ (10 mol%). ^d^ PMe_3_ (5 mol%).

## Data Availability

The data presented in this study are available on request from the authors.

## Supplementary Materials

The following supporting information can be downloaded at: https://www.mdpi.com/article/10.3390/molecules29112593/s1, Full experimental procedures, characterization data, NMR spectra for all new compounds, as well as crystallographic data for product **3sa** are available [61,62].
