# Supplementary material for: Phosphine-Catalyzed γ′-Carbon 1,6-Conjugate Addition of α-Succinimide Substituted Allenoates with Para-Quinone Methides: Synthesis of 4-Diarylmethylated 3,4-Disubstituted Maleimides
Source: Molecules. 2024 May 31;29(11):2593. doi: 10.3390/molecules29112593 (PMC11173458; doi:10.3390/molecules29112593)
Supplement: Supplementary file 1 [file molecules-29-02593-s001.zip › molecules-3016968-supplementary.pdf]

Supporting Information

Phosphine-Catalyzed γ'-Carbon 1,6-Conjugate Addition of α-Succinimide Substituted Allenoates with *para*-Quinone Methides: Synthesis of 4-Diarylmethylated 3,4-Disubstituted Maleimides

Zhenzhen Gao [1,*#], Xiaoming Zhou [1,#], Dandan Liu [1], Baoshen Nie [1], Xiaotong Chen [1], Jiahui Wu [1], Lei Li [1,*], XuekunWang [1,*]

[a]State Key Laboratory for Macromolecule Drugs and Large-scale Manufacturing, School of Pharmaceutical Sciences, Liaocheng University, Liaocheng, Shandong 252059, P. R. China.

Email: gaozhenzhen@lcu.edu.cn; leili@lcu.edu.cn; wangxuekun@lcu.edu.cn

Table of Contents

General Information S2

General Procedure for Preparation of aldimine **1** S2

General Procedure for Preparation of α-Succinimide-Substituted Allenoate **2** S2

General Procedure for Preparation of Products **3** S3

^{1}H and ^{13}C NMR Spectra of New Compounds S4-S44

X-Ray Crystallographic Data of **3ke** S45-S51

The ^{1}H NMR and ^{13}C NMR of **3pa** on the different temperature S66-S69

General Information

^{1}H and ^{13}C NMR spectra were recorded at 500 and 126 MHz. Reactions were monitored through thin layer chromatography (TLC) on silica gel-precoated glass plates and visualized under UV light at 254 nm. Flash column chromatography was performed using Qingdao Haiyang flash silica gel (100–200 mesh). The melting point was measured using a digital micromelting point meter X-4. X-ray crystallographic data were collected using Gemini E Rigaku. Mass spectrometry was performed using an Agilent 6520 Q-TOF mass spectrometer system equipped with an electrospray ionization (ESI) source.

All *para*-quinone methides were prepared by following a literature procedure.[1] α-succinimide-substituted allenoates were prepared according to literature procedure.[2]

---

[1] Goswami, P.; Anand, R. V. *Chemistry Select*. **2016**, 1, 2556 – 2559.

[2] Zhao Q Y, Pei C K, Guan X Y, et al. *Adv. Synth. Catal.* **2011**, 353, 1973 – 1979.

General Procedure for 1,6-conjugate addition reaction of α-Succinimide-Substituted Allenoate and *para*-quinone methides

Under argon atmosphere, to a mixture of *para*-quinone methide **1** (0.10 mmol), α-succinimide substituted allenoate **2** (0.12 mmol) and catalyst PMe_3 (20 mol %, 0.02 mmol) in a Schlenk tube, 1 mL of DCM was added at room temperature. The resulting mixture was stirred until the starting material was completely consumed (monitored by TLC) and then was concentrated to dryness. The residue was purified through flash column chromatography (EtOAc/PE) to afford the corresponding cycloaddition products **3**.

Characterization Data of the Products 3

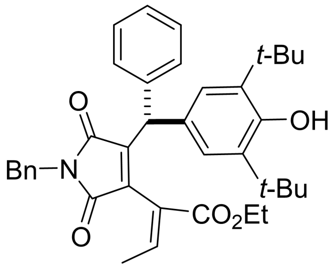

3aa

Yellow solid, 54.6mg, 92% yield. Purified by flash chromatography (12% EtOAc/PE). mp = 70.5- 71.5°C; ^{1}H NMR (500 MHz, DMSO) δ 7.35 – 7.24 (m, 6H), 7.22 (d, *J* = 6.8 Hz, 1H), 7.19 – 7.15 (m, 2H), 7.13 – 6.96 (m, 1H), 6.92 – 6.78 (m, 3H), 5.26 (s, 1H), 4.63 (s, 2H), 4.12 – 3.95 (m, 2H), 1.47 (s, 3H), 1.28 (s, 18H), 1.09 (t, *J* = 7.1 Hz, 3H). ^{13}C NMR (126 MHz, $CDCl_3$) δ 169.3, 168.6, 163.3, 151.8, 144.0, 139.3, 135.5, 135.0, 134.7, 127.7, 127.6, 127.3, 127.0, 126.6, 125.9, 124.9, 122.2, 60.1, 47.7, 40.7, 33.3, 33.3, 29.3, 29.2, 15.2, 12.9. HRMS (ESI) calcd for $C_{38}H_{44}NO_5$, $[M+H]^+$594.3219, found 594.3221.

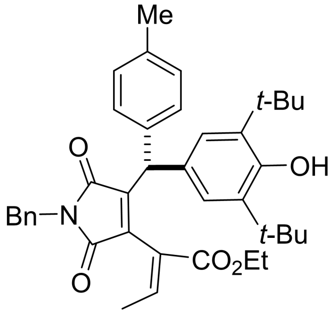

3ba

Yellow solid, 91% yield. Purified by flash chromatography (12% EtOAc/PE). mp = 68.0- 69.0°C; ^{1}H NMR (500 MHz, DMSO) δ 7.34 – 7.23 (m, 3H), 7.17 (d, *J* = 7.1 Hz, 3H), 7.09 – 6.96 (m, 3H), 6.91 – 6.78 (m, 3H), 5.20 (s, 1H), 4.63 (s, 2H), 4.06 (dd, *J* = 14.2, 7.0 Hz, 2H), 2.23 (s, 3H), 1.45 (s, 3H), 1.28 (s, 18H), 1.09 (t, *J* = 7.1 Hz, 3H). ^{13}C NMR (126 MHz, DMSO) δ 163.3, 161.7, 159.8, 151.9, 135.4, 135.1, 127.6, 127.1, 126.7, 124.8, 122.2, 114.2, 114.1, 60.2, 46.9, 42.4, 40.8, 33.3, 29.1, 15.2, 12.9. HRMS (ESI) calcd for $C_{39}H_{45}NO_5Na$, $[M+Na]^+$ 630.3195, found 630.3201.

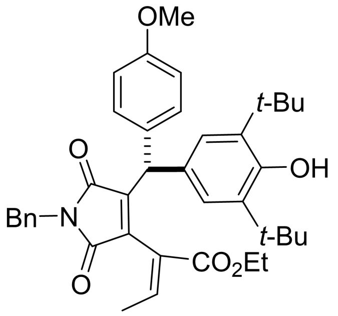

3ca

Yellow solid, 91% yield. Purified by flash chromatography (12% EtOAc/PE). mp = 63.5- 64.5°C; ^{1}H NMR (500 MHz, $CDCl_3$) δ 7.24 – 7.20 (m, 3H), 7.20 – 7.14 (m, 2H), 7.13 – 7.05 (m, 1H), 7.05 – 6.84 (m, 3H), 6.83 – 6.75 (m, 1H), 6.75 – 6.66 (m, 2H), 5.05 (s, 1H), 5.02 (s, 1H), 4.59 (s, 2H), 4.04 (q, J = 13.9, 7.1 Hz, 2H), 3.70 (s, 3H), 1.47 – 1.35 (m, 3H), 1.25 (s, 18H), 1.07 (t, J = 8.7 Hz, 3H). ^{13}C NMR (126 MHz, $CDCl_3$) δ 163.4, 157.4, 151.7, 135.5, 134.7, 128.8, 127.56, 127.1, 126.6, 124.8, 122.3, 112.7, 60.1, 54.2, 46.9, 40.7, 33.3, 29.2, 15.2, 12.9. HRMS (ESI) calcd for $C_{39}H_{45}NO_6Na$, $[M+Na]^+$ 646.3145, found 646.3145.

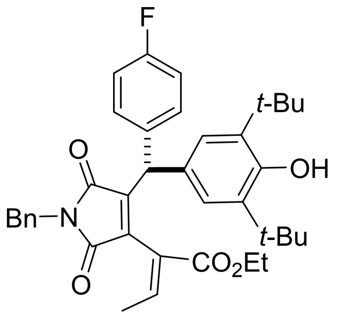

3da

Yellow solid, 84% yield. Purified by flash chromatography (12% EtOAc/PE). mp = 66.0- 67.0°C; ^{1}H NMR (500 MHz, $CDCl_3$) δ 7.25 – 7.15 (m, 6H), 7.15 – 6.98 (m, 2H), 6.92 – 6.82 (m, 3H), 6.81 – 6.67 (m, 1H), 5.07 (s, 1H), 5.05 (s, 1H), 4.60 (s, 2H), 4.10 – 3.98 (m, 2H), 1.39 (s, 3H), 1.25 (s, 18H), 1.11 – 1.03 (m, 3H). ^{13}C NMR (126 MHz, $CDCl_3$) δ 163.3, 161.7, 159.8, 151.85, 135.4, 135.1, 127.6, 127.1, 126.7, 124.8, 122.2, 114.2, 114.1, 60.2, 46.9, 42.4, 40.8, 33.3, 29.1, 15.2, 12.95. HRMS (ESI) calcd for $C_{38}H_{42}NO_5NaF$, $[M+Na]^+$ 634.2945, found 634.2940.

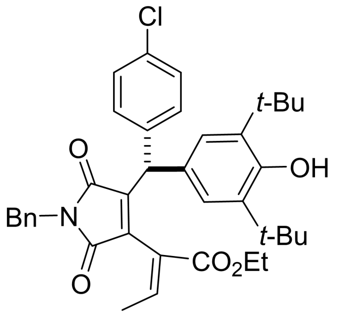

3ea

Yellow solid, 86% yield. Purified by flash chromatography (12% EtOAc/PE). mp = 63.0- 64.0°C; ^{1}H NMR (500 MHz, $CDCl_3$) δ 7.22 (s, 1H), 7.22 (s, 2H), 7.20 – 7.13 (m, 4H), 7.12 – 6.90 (m, 3H), 6.90 – 6.66 (m, 2H), 5.12 – 5.00 (m, 2H), 4.60 (s, 2H), 4.10 – 3.96 (m, 2H), 1.41 (d, J = 3.9 Hz, 3H), 1.25 (s, 18H), 1.08 (s, 3H). ^{13}C NMR (126 MHz, $CDCl_3$) δ 163.3, 151.9, 135.4, 135.2, 131.8, 129.1, 127.6, 127.5, 127.1, 126.7, 124.85, 122.1, 60.2, 47.1, 40.8, 33.3, 29.15, 25.9, 15.2, 13.0. HRMS (ESI) calcd for $C_{38}H_{43}ClNO_5$, $[M+H]^+$ 628.2830, found 628.2827.

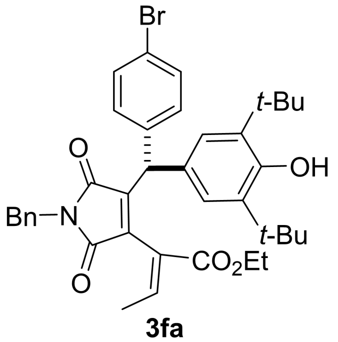

3fa

Yellow solid, 80% yield. Purified by flash chromatography (12% EtOAc/PE). mp = 54.0- 55.0°C; ^{1}H NMR (500 MHz, DMSO) δ 7.54 – 7.41 (m, 2H), 7.35 – 7.24 (m, 3H), 7.24 – 7.12 (m, 3H), 7.11 – 6.95 (m, 1H), 6.94 – 6.86 (m, 2H), 6.86 – 6.78 (m, 1H), 5.26 (s, 1H), 4.63 (s, 2H), 4.07 – 3.93 (m, 2H), 1.50 (d, J = 7.2 Hz, 3H), 1.28 (s, 18H), 1.15 – 0.99 (m, 3H). ^{13}C NMR (126 MHz, DMSO) δ 164.2, 153.3, 137.1, 136.2, 130.1, 129.0, 127.9, 127.1, 125.75, 122.9, 61.2, 47.8, 41.3, 35.0, 30.7, 16.4, 14.3. HRMS (ESI) calcd for $C_{38}H_{43}BrNO_5$, $[M+H]^+$ 672.2325, found 672.2320.

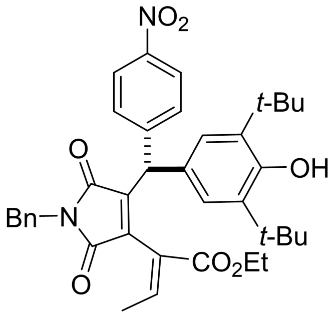

3ga

Yellow solid, 78% yield. Purified by flash chromatography (12% EtOAc/PE). mp = 82.0- 83.0°C; ^{1}H NMR (500 MHz, $CDCl_3$) δ 8.06 (d, J = 8.3 Hz, 2H), 7.40 – 7.25 (m, 2H), 7.24 – 7.21 (m, 4H), 7.21 – 7.19 (m, 1H), 7.19 – 7.17 (m, 1H), 6.98 – 6.71 (m, 2H), 5.27 – 5.04 (m, 2H), 4.60 (s, 2H), 4.13 – 3.97 (m, 2H), 1.41 (d, J = 7.1 Hz, 3H), 1.26 (s, 18H), 1.15 – 1.05 (m, 3H). ^{13}C NMR (126 MHz, $CDCl_3$) δ 163.2, 152.2, 145.9, 135.8, 135.2, 128.5, 127.7, 127.1, 126.8, 124.9, 122.5, 122.0, 60.3, 47.5, 40.9, 33.3, 29.1, 25.9, 15.2, 13.0. HRMS (ESI) calcd for $C_{32}H_{32}N_2O_6NaS$, $[M+Na]^+$ 661.2890, found 661.2885.

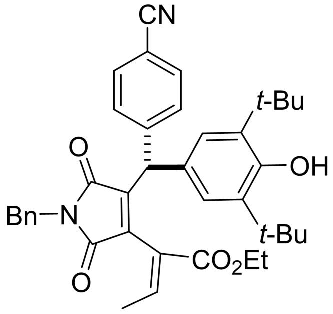

3ha

Yellow solid, 79% yield. Purified by flash chromatography (12% EtOAc/PE). mp = 46.0- 47.0°C; ^{1}H NMR (500 MHz, DMSO) δ 8.31 (s, 1H), 7.86 – 7.68 (m, 2H), 7.49 – 7.37 (m, 1H), 7.36 – 7.24 (m, 4H), 7.22 – 7.11 (m, 2H), 6.93 – 6.77 (m, 3H), 5.40 (s, 1H), 4.63 (s, 2H), 4.09 – 3.92 (m, 2H), 1.51 (s, 3H), 1.28 (s, 18H), 1.15 – 0.98 (m, 3H).^{13}C NMR (126 MHz, DMSO) δ 164.1, 153.4, 137.1, 136.6, 132.6, 130.0, 129.0, 127.9, 127.3, 125.8, 122.8, 119.2, 110.1, 61.2, 48.1, 41.4, 34.9, 30.6, 16.4, 14.2. HRMS (ESI) calcd for $C_{39}H_{43}N_2O_5$, $[M+Na]^+$ 619.3172, found 619.3171.

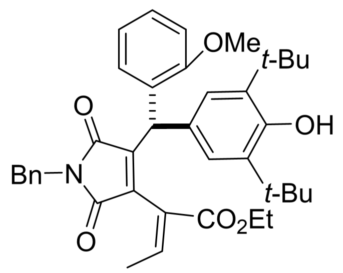

3ia

Yellow solid, 84% yield. Purified by flash chromatography (12% EtOAc/PE). mp = 58.0- 59.0°C; ^{1}H NMR (500 MHz, $CDCl_3$) δ 7.26 – 7.16 (m, 5H), 7.15 – 7.03 (m, 2H), 6.94 – 6.85 (m, 2H), 6.84 – 6.65 (m, 3H), 5.44 (s, 1H), 4.99 (s, 1H), 4.59 (s, 2H), 4.11 – 3.75 (m, 2H), 3.62 (s, 3H), 1.46 – 1.35 (m, 3H), 1.25 (s, 18H), 1.09 – 0.96 (m, 3H). ^{13}C NMR (126 MHz, $CDCl_3$) δ 169.5, 163.4, 155.8, 151.5, 135.7, 127.9, 127.6, 127.1, 126.6, 124.8, 122.4, 119.2, 59.8, 41.0, 40.6, 33.3, 29.2, 25.9, 16.0, 13.0. HRMS (ESI) calcd for $C_{39}H_{45}NO_6Na$, $[M+Na]^+$ 646.3145, found 646.3148.

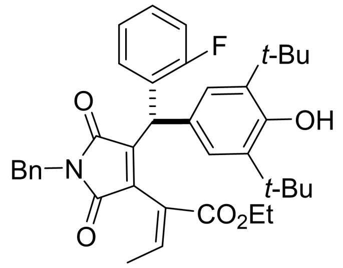

3ja

Yellow solid, 92% yield. Purified by flash chromatography (12% EtOAc/PE). mp = 60.0- 61.0°C; ^{1}H NMR (500 MHz, $CDCl_3$) δ 7.25 – 7.20 (m, 4H), 7.20 – 7.17 (m, 2H), 7.17 – 7.10 (m, 1H), 7.01 – 6.86 (m, 4H), 6.84 – 6.76 (m, 1H), 5.37 (s, 1H), 5.04 (s, 1H), 4.61 (s, 2H), 4.00 (q, J = 24.0, 12.0, 5.7 Hz, 2H), 1.45 (s, 3H), 1.26 (s, 18H), 1.09 – 0.93 (m, 3H). ^{13}C NMR (126 MHz, $CDCl_3$) δ 168.8, 163.3, 151.8, 135.0, 134.8, 134.7, 133.3, 129.4, 127.7, 127.3, 126.8, 126.5, 125.9, 125.1, 124.9, 122.3, 60.1, 47.7, 38.5, 33.3, 29.2, 18.3, 15.2, 13.0. HRMS (ESI) calcd for $C_{38}H_{42}FNO_5Na$, $[M+Na]^+$ 634.2945, found 634.2949.

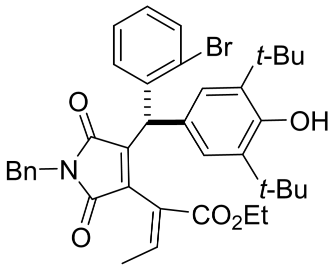

3ka

Yellow solid, 87% yield. Purified by flash chromatography (12% EtOAc/PE). mp = 67.0- 68.0°C; ^{1}H NMR (500 MHz, $CDCl_3$) δ 7.50 – 7.37 (m, 1H), 7.26 – 7.21 (m, 3H), 7.21 – 7.11 (m, 3H), 7.10 – 6.96 (m, 2H), 6.89 (t, *J* = 21.1 Hz, 1H), 6.85 – 6.78 (m, 1H), 6.74 (s, 1H), 5.58 (s, 1H), 5.03 (s, 1H), 4.63 (s, 2H), 4.09 – 3.86 (m, 2H), 1.43 (s, 3H), 1.25 (s, 18H), 1.14 – 1.00 (m, 3H). ^{13}C NMR (126 MHz, $CDCl_3$) δ 164.3, 152.9, 144.6, 136.5, 136.5, 128.7, 128.1, 127.7, 127.2, 126.0, 48.2, 41.9, 34.3, 30.1, 26.9, 16.34, 14.0. HRMS (ESI) calcd for $C_{38}H_{42}NO_5BrK$, $[M+K]^+$ 710.1883, found 710.1873.

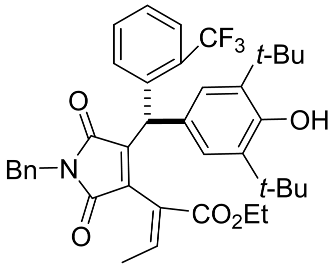

3la

Yellow solid, 88% yield. Purified by flash chromatography (12% EtOAc/PE). mp = 69.0- 70.0°C; ^{1}H NMR (500 MHz, $CDCl_3$) δ 7.61 – 7.33 (m, 2H), 7.32 – 7.20 (m, 5H), 7.20 – 7.07 (m, 2H), 6.87 (s, 1H), 6.79 – 6.62 (m, 2H), 5.59 (d, *J* = 13.4 Hz, 1H), 5.02 (d, *J* = 16.8 Hz, 1H), 4.62 (s, 2H), 4.11 – 3.90 (m, 2H), 1.40 – 1.29 (m, 3H), 1.27 – 1.19 (m, 18H), 1.15 – 1.06 (m, 3H). ^{13}C NMR (126 MHz, $CDCl_3$) δ 162.9, 151.8, 143.3, 135.4, 134.4, 130.5, 130.1, 127.6, 127.0, 126.7, 124.5, 122.0, 60.0, 43.6, 43.2, 40.9, 33.2, 33.2, 29.2, 29.0, 14.8, 13.1, 12.9. HRMS (ESI) calcd for $C_{39}H_{42}NO_5F_3Na$, $[M+Na]^+$ 684.2913, found 684.2909.

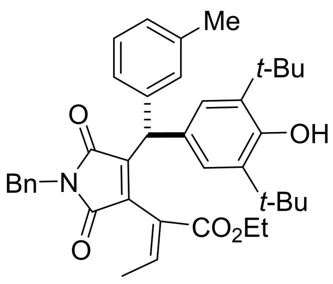

3ma

Yellow solid, 87% yield. Purified by flash chromatography (12% EtOAc/PE). mp = 66.0- 67.0°C; ^{1}H NMR (500 MHz, $CDCl_3$) δ 7.22 (m, 3H), 7.20 – 7.14 (m, 2H), 7.06 (m, 1H), 7.03 – 6.70 (m, 6H), 5.06 (s, 1H), 5.03 (s, 1H), 4.60 (s, 2H), 4.04 (q, *J* = 10.2, 6.8 Hz, 2H), 2.20 (s, 3H), 1.40 (s, 3H), 1.26 (s, 18H), 1.06 (t, *J* = 7.1 Hz, 3H). ^{13}C NMR (126 MHz, $CDCl_3$) δ 163.3, 151.7, 135.5, 127.6, 127.1, 127.0, 126.6, 124.9, 124.8, 122.2, 60.1, 47.6, 40.7, 33.3, 29.2, 20.4, 15.2, 13.0. HRMS (ESI) calcd for $C_{39}H_{45}NO_5Na$, $[M+Na]^+$ 630.3195, found 630.3198.

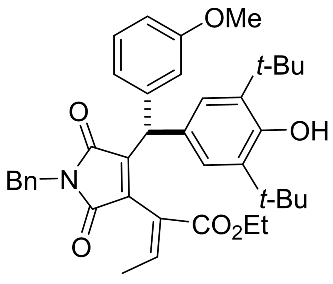

3na

Yellow solid, 89% yield. Purified by flash chromatography (12% EtOAc/PE). mp = 65.0- 66.0°C; ^{1}H NMR (500 MHz, $CDCl_3$) δ 7.24 – 7.20 (m, 2H), 7.20 (s, 1H), 7.19 – 7.15 (m, 1H), 7.15 – 7.01 (m, 2H), 7.00 – 6.70 (m, 4H), 6.70 – 6.53 (m, 2H), 5.06 (s, 1H), 5.03 (s, 1H), 4.60 (s, 2H), 4.03 (s, 2H), 3.64 (s, 3H), 1.41 (s, 3H), 1.25 (s, 18H), 1.06 (t, *J* = 6.4 Hz, 3H). ^{13}C NMR (126 MHz, $CDCl_3$) δ 163.3, 158.6, 151.8, 135.5, 135.0, 134.6, 128.2, 127.6, 127.0, 126.6, 124.9, 122.2, 120.2, 113.8, 111.2, 60.1, 54.1, 47.7, 40.7, 33.3, 29.3, 29.2, 25.9, 15.2, 12.9. HRMS (ESI) calcd for $C_{39}H_{46}NO_6$, $[M+Na]^+$ 624.3325, found 624.3317.

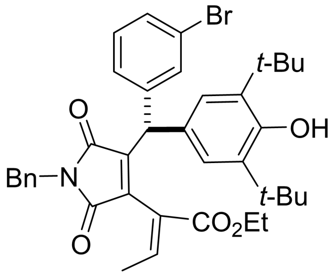

3oa

Yellow solid, 68% yield. Purified by flash chromatography (12% EtOAc/PE). mp = 67.5- 68.5°C; ^{1}H NMR (500 MHz, DMSO) δ 7.52 – 7.37 (m, 1H), 7.35 – 7.22 (m, 4H), 7.22 – 7.13 (m, 2H), 7.11 – 6.89 (m, 3H), 6.89 – 6.75 (m, 2H), 5.32 (s, 1H), 4.64 (s, 2H), 4.12 – 3.95 (m, 2H), 1.49 (d, J = 5.8 Hz, 3H), 1.28 (s, 18H), 1.10 (dd, J = 7.0, 3.6 Hz, 3H). ^{13}C NMR (126 MHz, $CDCl_3$) δ 163.3, 151.7, 135.5, 134.9, 134.6, 127.6, 127.1, 127.0, 126.6, 126.6, 124.9, 124.8, 122.3, 60.1, 47.6, 40.7, 33.3, 29.2, 20.4, 15.2, 13.0. HRMS (ESI) calcd for $C_{38}H_{42}NO_5BrNa$, $[M+Na]^+$ 694.2144, found 694.2138.

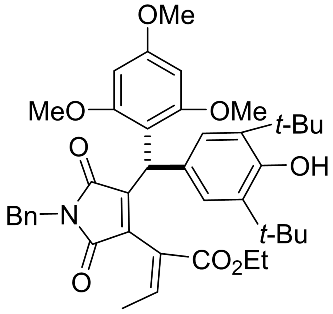

3pa

Yellow solid, 82% yield. Purified by flash chromatography (12% EtOAc/PE). mp = 76.0- 77.0°C; ^{1}H NMR (500 MHz, $CDCl_3$) δ 7.25 – 7.22 (m, 2H), 7.22 – 7.20 (m, 1H), 7.20 – 7.15 (m, 2H), 7.08 – 6.74 (m, 3H), 6.59 – 6.21 (m, 2H), 5.05 (s, 2H), 4.61 (s, 2H), 4.03 (s, 2H), 3.75 (s, 3H), 3.66 (d, J = 4.4 Hz, 6H), 1.49 – 1.35 (m, 3H), 1.33 – 1.20 (m, 18H), 1.07 (t, J = 7.1 Hz, 3H). ^{13}C NMR (126 MHz, $CDCl_3$) δ 163.4, 152.0, 151.8, 135.9, 135.5, 127.6, 127.0, 126.7, 124.8, 122.3, 60.1, 59.8, 55.1, 47.8, 40.7, 33.3, 29.2, 15.3, 12.9. HRMS (ESI) calcd for $C_{41}H_{49}NO_8Na$, $[M+Na]^+$ 706.3356, found 706.3365.

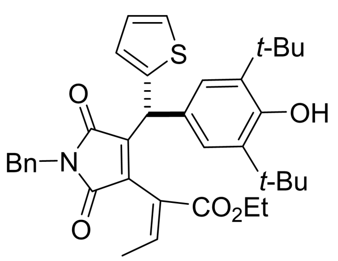

3qa

Yellow solid, 68% yield. Purified by flash chromatography (12% EtOAc/PE). mp = 63.0- 64.0°C; ^{1}H NMR (500 MHz, $CDCl_3$) δ 7.26 – 7.20 (m, 3H), 7.20 – 7.15 (m, 2H), 7.14 – 6.87 (m, 4H), 6.86 – 6.69 (m, 2H), 5.26 (s, 1H), 5.06 (s, 1H), 4.62 (s, 2H), 4.14 – 3.91 (m, 2H), 1.49 (d, *J* = 8.6 Hz, 3H), 1.28 (s, 18H), 1.10 – 0.98 (m, 3H). ^{13}C NMR (126 MHz, $CDCl_3$) δ 163.3, 152.1, 144.4, 135.4, 134.7, 134.4, 127.6, 127.0, 126.6, 125.7, 122.0, 60.2, 40.7, 33.3, 29.2, 25.9, 15.3, 12.9. HRMS (ESI) calcd for $C_{36}H_{41}NO_5SK$, $[M+K]^+$ 638.2343, found 638.2346.

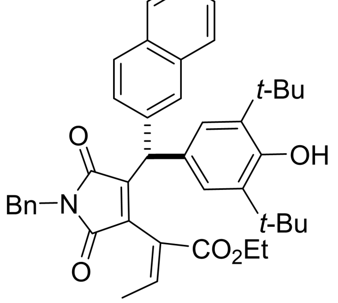

3ra

Yellow solid, 95% yield. Purified by flash chromatography (12% EtOAc/PE). mp = 73.5- 74.5°C; ^{1}H NMR (500 MHz, $CDCl_3$) δ 7.75 – 7.62 (m, 3H), 7.59 – 7.41 (m, 1H), 7.41 – 7.32 (m, 2H), 7.28 – 7.20 (m, 4H), 7.20 – 7.15 (m, 2H), 7.08 – 6.74 (m, 3H), 5.28 (s, 1H), 5.05 (s, 1H), 4.61 (s, 2H), 4.03 (dd, *J* = 31.1, 6.6 Hz, 2H), 1.46 – 1.34 (m, 3H), 1.26 (d, *J* = 6.7 Hz, 18H), 1.03 (t, *J* = 7.1 Hz, 3H). ^{13}C NMR (126 MHz, $CDCl_3$) δ 163.3, 151.8, 135.5, 135.2, 132.3, 131.4, 127.6, 127.1, 126.8, 126.6, 126.6, 125.0, 124.75, 122.2, 60.1, 47.7, 40.8, 33.3, 29.2, 25.9, 15.2, 12.95. HRMS (ESI) calcd for $C_{42}H_{45}NO_5K$, $[M+K]^+$ 682.2935, found 682.2938.

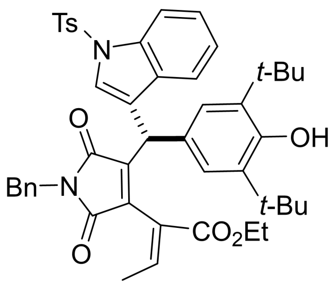

3sa

Yellow solid, 88% yield. Purified by flash chromatography (12% EtOAc/PE). mp = 103.0- 104.0°C; ^{1}H NMR (500 MHz, $CDCl_3$) δ 7.88 (d, *J* = 8.3 Hz, 1H), 7.57 (d, *J* = 8.2 Hz, 2H), 7.30 – 7.22 (m, 3H), 7.21 – 7.15 (m, 3H), 7.10 – 7.05 (m, 2H), 7.05 – 6.99 (m, 1H), 6.79 (s, 1H), 5.22 (s, 1H), 5.04 (s, 1H), 4.64 (s, 2H), 4.20 – 3.80 (m, 2H), 2.25 (s, 3H), 1.50 (s, 3H), 1.22 (s, 18H), 1.10 – 0.93 (m, 3H). ^{13}C NMR (126 MHz, $CDCl_3$) δ 164.3, 155.6, 153.0, 144.7, 136.5, 135.35, 134.3, 129.8, 128.7, 127.85, 127.7, 126.7, 125.5, 124.8, 123.1, 119.8, 113.8, 61.3, 41.8, 34.3, 30.2, 26.9, 21.5, 13.9. HRMS (ESI) calcd for $C_{47}H_{50}N_2O_7NaS$, $[M+Na]^+$ 809.3236, found 809.3225.

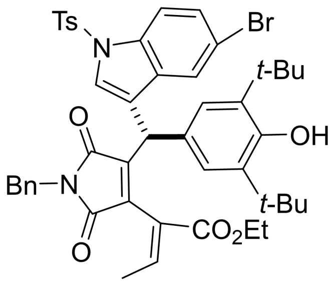

3ta

Yellow solid, 84% yield. Purified by flash chromatography (12% EtOAc/PE). mp = 104- 105°C; ^{1}H NMR (500 MHz, $CDCl_3$) δ 7.75 (d, *J* = 8.8 Hz, 1H), 7.56 (d, *J* = 8.0 Hz, 2H), 7.32 – 7.26 (m, 1H), 7.27 – 7.19 (m, 5H), 7.13 – 7.06 (m, 2H), 6.78 (s, 1H), 5.23 – 5.11 (m, 1H), 5.08 (s, 1H), 4.64 (s, 2H), 4.17 – 3.90 (m, 2H), 2.27 (s, 3H), 1.30 (d, *J* = 7.1 Hz, 3H), 1.23 (s, 18H), 1.05 (t, *J* = 7.0 Hz, 3H). ^{13}C NMR (126 MHz, $CDCl_3$) δ 164.3, 153.2, 145.68, 145.1, 136.4, 134.9, 134.0, 129.9, 128.8, 127.8, 127.75, 126.7, 125.5, 122.9, 122.5, 116.8, 115.3, 61.4, 41.8, 34.3, 30.2, 30.2, 21.6, 16.2, 14.0. HRMS (ESI) calcd for $C_{47}H_{49}BrN_2O_7NaS$, $[M+Na]^+$ 887.2342, found 887.2351.

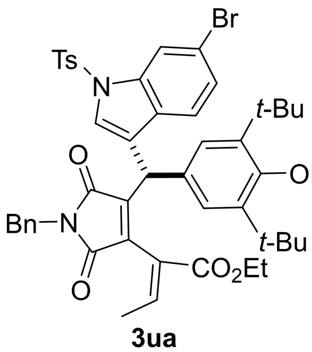

3ua

Yellow solid, 85% yield. Purified by flash chromatography (12% EtOAc/PE). mp = 117.5- 118.5°C; ^{1}H NMR (500 MHz, $CDCl_3$) δ 8.06 (s, 1H), 7.57 (d, *J* = 8.2 Hz, 2H), 7.26 – 7.19 (m, 4H), 7.15 – 7.08 (m, 3H), 7.00 – 6.86 (m, 2H), 5.21 (d, *J* = 16.6 Hz, 1H), 5.06 (s, 1H), 4.63 (s, 2H), 4.17 – 3.93 (m, 2H), 2.27 (s, 3H), 1.25 (s, 3H), 1.21 (s, 18H), 1.07 (t, *J* = 7.2 Hz, 3H). ^{13}C NMR (126 MHz, $CDCl_3$) δ 163.2, 152.1, 144.1, 135.4, 135.0, 134.0, 129.0, 128.0, 127.7, 126.8, 126.7, 125.7, 125.5, 124.9, 124.4, 121.9, 120.0, 117.6, 115.8, 60.35, 40.8, 33.2, 29.2, 29.1, 28.7, 20.6, 12.9. HRMS (ESI) calcd for $C_{47}H_{49}BrN_2O_7NaS$, $[M+Na]^+$ 887.2342, found 887.2345.

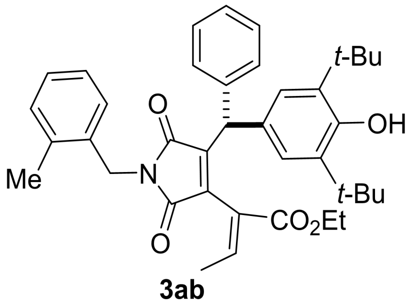

3ab

Yellow solid, 74% yield. Purified by flash chromatography (12% EtOAc/PE). mp = 62- 63°C; ^{1}H NMR (500 MHz, DMSO) δ 7.37 – 7.19 (m, 4H), 7.18 – 7.03 (m, 4H), 6.94 (d, *J* = 7.5 Hz, 1H), 6.92 – 6.83 (m, 2H), 6.83 – 6.76 (m, 1H), 5.25 (s, 1H), 4.61 (s, 2H), 4.11 – 3.97 (m, 2H), 2.29 (s, 3H), 1.48 (d, *J* = 5.6 Hz, 3H), 1.33 – 1.23 (m, 18H), 1.09 (t, *J* = 7.1 Hz, 3H). ^{13}C NMR (126 MHz, $CDCl_3$) δ 168.8, 163.3, 151.8, 135.0, 134.8, 134.7, 133.3, 129.4, 127.7, 127.3, 126.8, 126.5, 125.7, 125.1, 124.9, 122.3, 60.1, 47.7, 38.5, 33.3, 29.2, 18.3, 15.2, 13.0. HRMS (ESI) calcd for $C_{39}H_{46}NO_5$, $[M+H]^+$ 608.3376, found 608.3377.

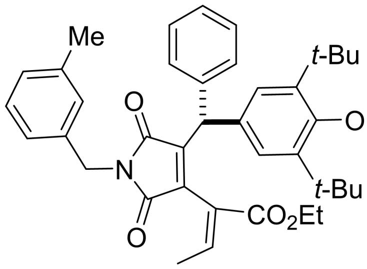

3ac

Yellow solid, 86% yield. Purified by flash chromatography (12% EtOAc/PE). mp = 63- 64°C; ^{1}H NMR (500 MHz, $CDCl_3$) δ 7.22 – 7.15 (m, 2H), 7.14 – 7.09 (m, 4H), 7.09 – 7.03 (m, 1H), 7.00 (d, J = 7.9 Hz, 2H), 6.98 – 6.82 (m, 2H), 6.82 – 6.69 (m, 1H), 5.09 (s, 1H), 5.03 (s, 1H), 4.55 (s, 2H), 4.02 (d, J = 5.0 Hz, 2H), 2.22 (s, 3H), 1.40 (s, 3H), 1.24 (s, 18H), 1.06 (t, J = 7.1 Hz, 3H). ^{13}C NMR (126 MHz, DMSO) δ 164.2, 153.4, 138.2, 137.1, 129.0, 128.9, 127.8, 127.2, 125.8, 124.3, 61.1, 56.5, 48.4, 40.5, 40.4, 34.9, 30.6, 21.4, 19.0, 16.3. HRMS (ESI) calcd for $C_{39}H_{45}NO_5Na$, $[M+Na]^+$ 630.3195, found 630.3201.

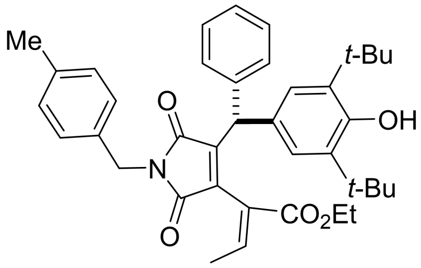

3ad

Yellow solid, 88% yield. Purified by flash chromatography (12% EtOAc/PE). mp = 74- 75°C; ^{1}H NMR (500 MHz, $CDCl_3$) δ 7.25 – 7.16 (m, 3H), 7.14 (d, J = 9.8 Hz, 2H), 7.12 – 7.05 (m, 2H), 7.05 – 6.96 (m, 3H), 6.95 – 6.85 (m, 1H), 6.85 – 6.70 (m, 1H), 5.10 (s, 1H), 5.03 (s, 1H), 4.56 (s, 2H), 4.03 (d, J = 6.2 Hz, 2H), 2.23 (s, 3H), 1.40 (s, 3H), 1.25 (s, 18H), 1.06 (t, J = 7.1 Hz, 3H). ^{13}C NMR (126 MHz, $CDCl_3$) δ 168.7, 163.3, 151.7, 136.3, 135.0, 134.6, 132.5, 128.25, 127.7, 127.3, 127.1, 125.85, 124.9, 122.2, 60.1, 47.7, 40.5, 33.3, 29.1, 28.7, 20.1, 15.2, 12.9. HRMS (ESI) calcd for $C_{39}H_{45}NO_5Na$, $[M+Na]^+$ 630.3195, found 630.3201.

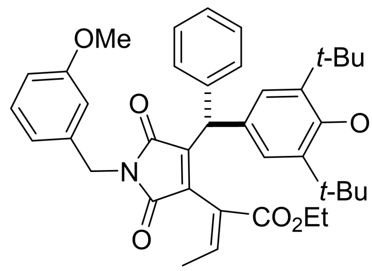

3ae

Yellow solid, 82% yield. Purified by flash chromatography (12% EtOAc/PE). mp = 68- 69°C; ^{1}H NMR (500 MHz, $CDCl_3$) δ 7.24 – 7.20 (m, 2H), 7.20 (s, 1H), 7.19 – 7.15 (m, 1H), 7.15 – 7.01 (m, 2H), 7.00 – 6.70 (m, 4H), 6.70 – 6.53 (m, 2H), 5.06 (s, 1H), 5.03 (s, 1H), 4.60 (s, 2H), 4.03 (s, 2H), 3.64 (s, 3H), 1.41 (s, 3H), 1.25 (s, 18H), 1.06 (t, *J* = 6.4 Hz, 3H). ^{13}C NMR (126 MHz, $CDCl_3$) δ 163.3, 158.6, 151.8, 135.5, 135.0, 134.64, 128.2, 127.6, 127.0, 126.6, 124.9, 122.2, 120.2, 113.8, 111.2, 60.1, 54.1, 47.7, 40.7, 33.3, 29.3, 29.2, 25.9, 15.2, 12.9. HRMS (ESI) calcd for $C_{39}H_{45}NO_6Na$, $[M+Na]^+$ 646.3145, found 646.3146.

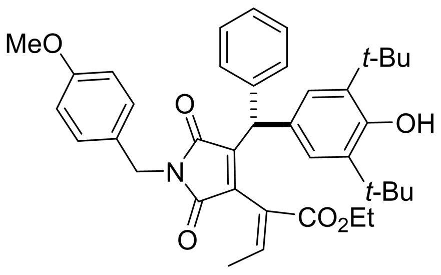

3af

Yellow solid, 81 % yield. Purified by flash chromatography (12% EtOAc/PE). mp = 64- 65°C; ^{1}H NMR (500 MHz, DMSO) δ 7.33 – 7.16 (m, 4H), 7.11 (d, *J* = 8.6 Hz, 2H), 7.08 – 6.92 (m, 1H), 6.90 – 6.76 (m, 5H), 5.23 (s, 1H), 4.55 (s, 2H), 4.03 (d, *J* = 5.7 Hz, 2H), 3.72 (s, 3H), 1.44 (s, 3H), 1.26 (s, 18H), 1.08 (t, *J* = 7.1 Hz, 3H). ^{13}C NMR (126 MHz, DMSO) δ 164.2, 159.1, 153.1, 141.0, 129.1, 129.0, 128.9, 128.7, 127.2, 125.8, 114.4, 79.6, 61.1, 55.5, 48.4, 34.9, 30.6, 16.4, 14.3. HRMS (ESI) calcd for $C_{39}H_{46}NO_6$, $[M+Na]^+$ 624.3325, found 624.3322.

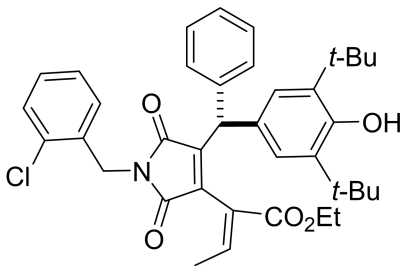

3ag

Yellow solid, 55% yield. Purified by flash chromatography (12% EtOAc/PE). mp = 60.5- 61.5°C; ^{1}H NMR (500 MHz, DMSO) δ 7.46 (dd, *J* = 7.8, 1.3 Hz, 1H), 7.36 – 7.19 (m, 6H), 7.11 (d, *J* = 7.1 Hz, 1H), 7.01 (s, 1H), 6.88 (dd, *J* = 14.2, 6.7 Hz, 3H), 5.26 (s, 1H), 4.70 (s, 2H), 4.04 (s, 2H), 1.48 (s, 3H), 1.27 (s, 18H), 1.10 (t, *J* = 7.1 Hz, 3H). ^{13}C NMR (126 MHz, $CDCl_3$) δ 163.3, 161.7, 159.8, 151.9, 135.4, 135.1, 127.6, 127.1, 126.7, 124.8, 122.2, 114.2, 114.1, 60.2, 46.9, 42.4, 40.8, 33.3, 29.1, 15.2, 13.0. HRMS (ESI) calcd for $C_{38}H_{43}ClNO_5$, $[M+H]^+$ 628.2830, found 628.2827.

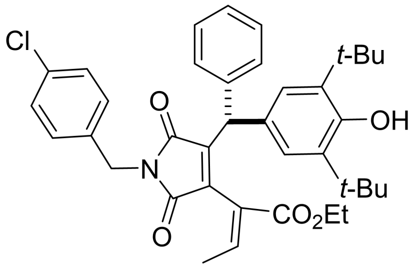

3ah

Yellow solid, 34% yield. Purified by flash chromatography (12% EtOAc/PE). mp = 60.0- 61.0°C; ^{1}H NMR (500 MHz, $CDCl_3$) δ 7.36 – 7.26 (m, 2H), 7.23 – 7.20 (m, 3H), 7.20 – 7.18 (m, 1H), 7.18 – 7.14 (m, 1H), 7.12 – 6.84 (m, 4H), 6.84 – 6.66 (m, 1H), 5.07 (s, 1H), 5.01 (s, 1H), 4.59 (s, 2H), 4.13 – 3.95 (m, 2H), 1.40 (d, *J* = 5.0 Hz, 3H), 1.25 (s, 18H), 1.07 (s, 3H). ^{13}C NMR (126 MHz, $CDCl_3$) δ 169.4, 163.2, 151.9, 135.4, 135.3, 134.8, 130.4, 129.5, 127.6, 127.1, 126.7, 124.9, 122.1, 119.9, 60.2, 47.1, 40.8, 33.3, 29.1, 15.2, 13.0. HRMS (ESI) calcd for $C_{38}H_{43}ClNO_5$, $[M+H]^+$ 628.2830, found 628.2832.

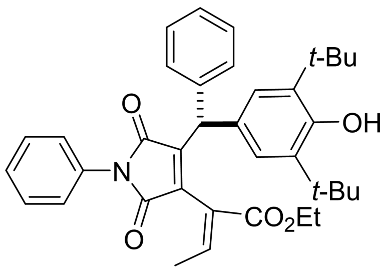

3ai

Yellow solid, 84% yield. Purified by flash chromatography (12% EtOAc/PE). mp = 52.0- 53.0°C; ^{1}H NMR (500 MHz, DMSO) δ 7.53 – 7.44 (m, 2H), 7.43 – 7.36 (m, 1H), 7.36 – 7.26 (m, 5H), 7.26 – 7.11 (m, 2H), 6.94 – 6.76 (m, 3H), 5.33 (s, 1H), 4.15 – 3.99 (m, 2H), 1.48 (s, 3H), 1.30 (s, 18H), 1.21 – 1.08 (m, 3H). ^{13}C NMR (126 MHz, $CDCl_3$) δ 167.7, 163.3, 151.9, 146.1, 144.2, 139.2, 134.9, 134.6, 130.8, 128.8, 127.9, 127.5, 126.4, 125.9, 125.1, 124.9, 124.6, 122.2, 60.2, 47.9, 33.3, 29.2, 29.2, 28.7, 15.3, 13.1. HRMS (ESI) calcd for $C_{37}H_{41}NO_5Na$, $[M+Na]^+$ 602.2882, found 602.2885.

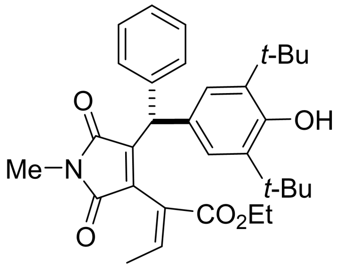

3aj

Liquid, 74% yield. ^{1}H NMR (500 MHz, DMSO) δ 7.39 – 7.25 (m, 3H), 7.24 – 7.19 (m, 1H), 7.05 – 6.91 (m, 1H), 6.89 – 6.73 (m, 3H), 5.22 (s, 1H), 4.06 (d, J = 6.7 Hz, 2H), 2.89 (s, 3H), 1.57 – 1.35 (m, 3H), 1.26 (s, 18H), 1.19 – 1.06 (m, 3H). ^{13}C NMR (126 MHz, DMSO) δ 164.4, 153.2, 145.1, 141.2, 129.0, 128.7, 127.1, 126.1, 79.6, 61.1, 48.5, 34.9, 30.6, 24.4, 14.4. HRMS (ESI) calcd for $C_{32}H_{40}NO_5$, $[M+H]^+$ 517.2828, found 517.2826.

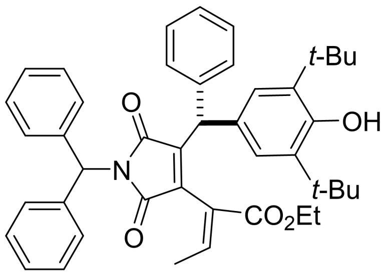

3ak

Yellow solid, 57% yield. Purified by flash chromatography (12% EtOAc/PE). mp = 86.0- 87.0°C; ^{1}H NMR (500 MHz, $CDCl_3$) δ 7.25 – 7.20 (m, 8H), 7.19 – 7.11 (m, 7H), 7.03 (s, 1H), 6.97 – 6.86 (m, 1H), 6.86 – 6.73 (m, 1H), 6.44 (s, 1H), 5.13 (d, *J* = 20.6 Hz, 1H), 5.06 – 4.98 (m, 1H), 4.11 – 3.91 (m, 2H), 1.39 (s, 3H), 1.26 (s, 18H), 1.08 (t, *J* = 7.1 Hz, 3H). ^{13}C NMR (126 MHz, $CDCl_3$) δ 167.4, 163.2, 151.9, 144.4, 139.1, 135.1, 134.7, 132.0, 129.3, 128.0, 127.7, 127.4, 126.0, 125.7, 125.2, 122.1, 60.2, 47.9, 33.3, 29.2, 28.7, 15.3, 13.1. HRMS (ESI) calcd for $C_{44}H_{47}NO_5Na$, $[M+Na]^+$ 692.3352, found 692.3356.

^{1}H NMRand ^{13}C NMR Spectra

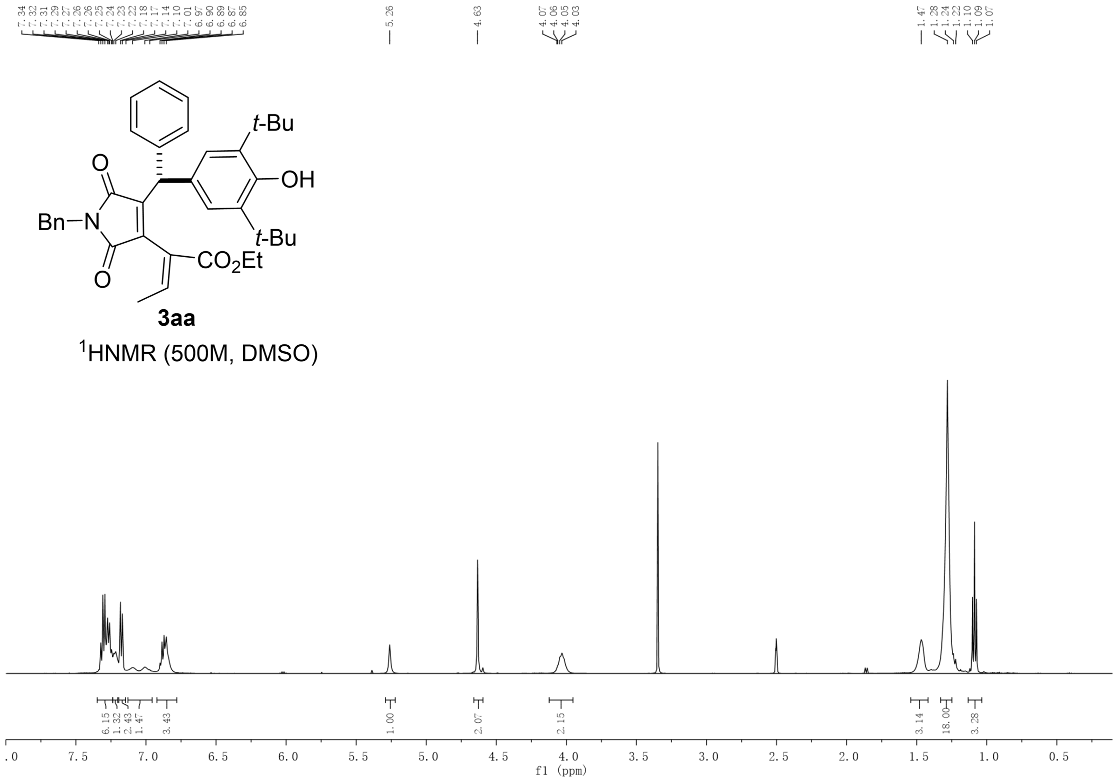

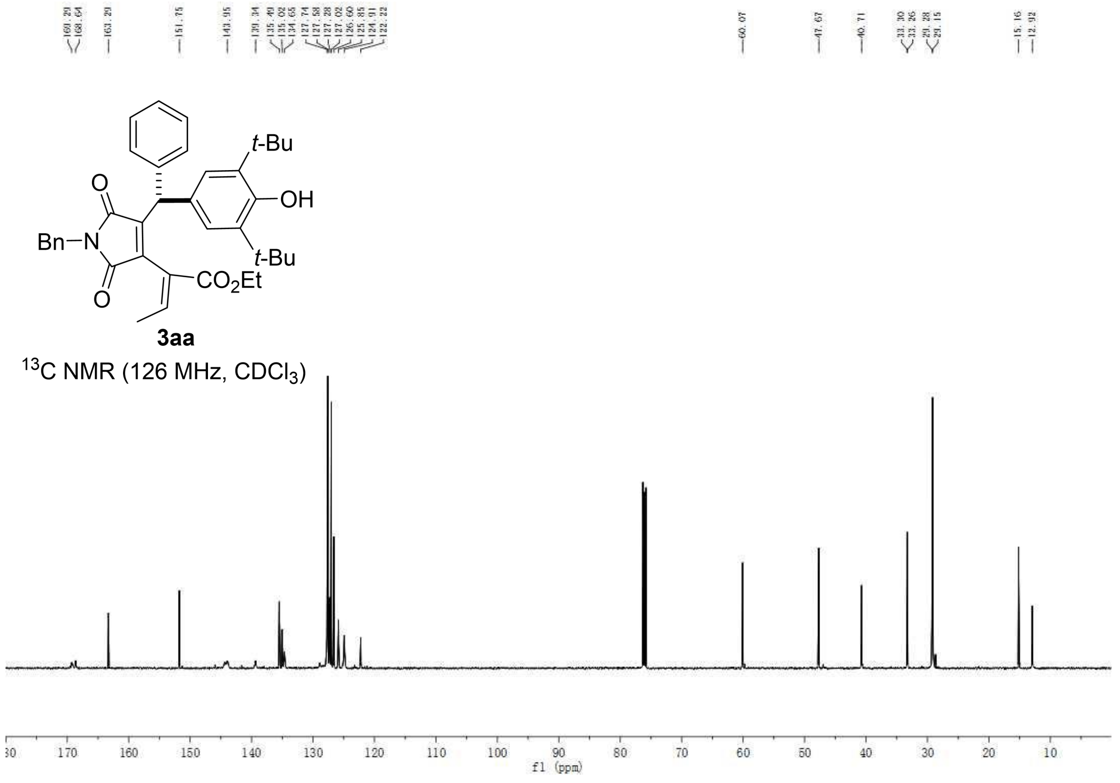

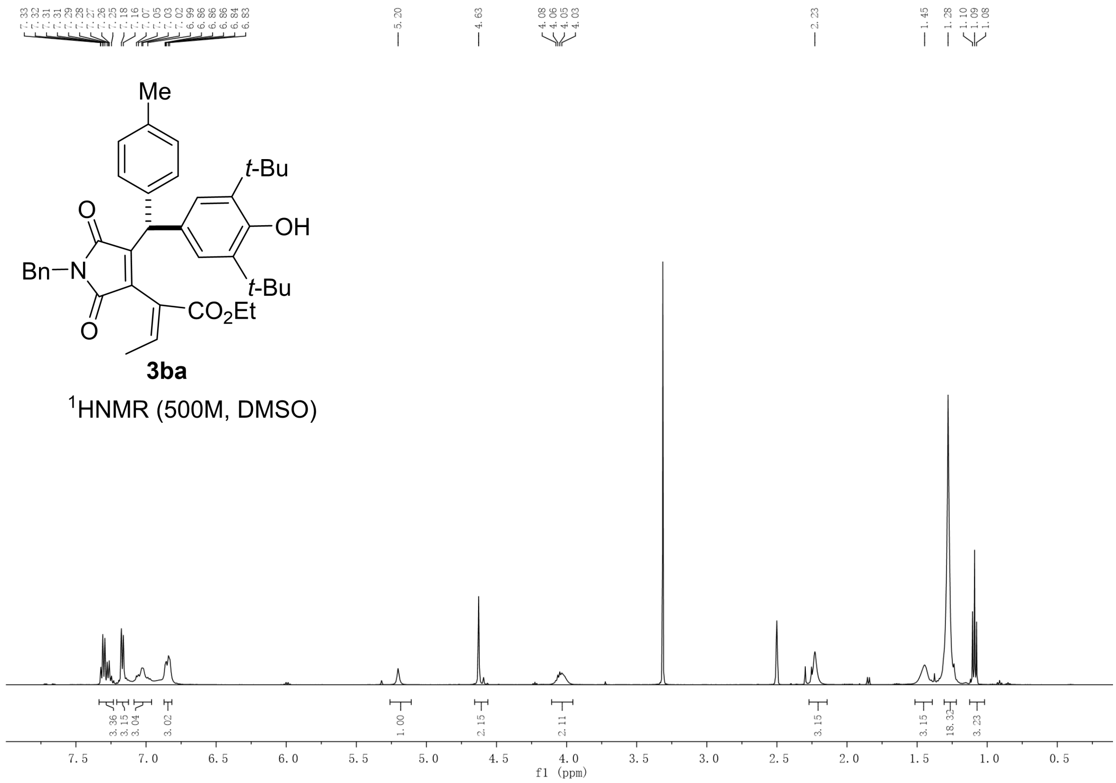

3ba

^{1}HNMR (500M, DMSO)

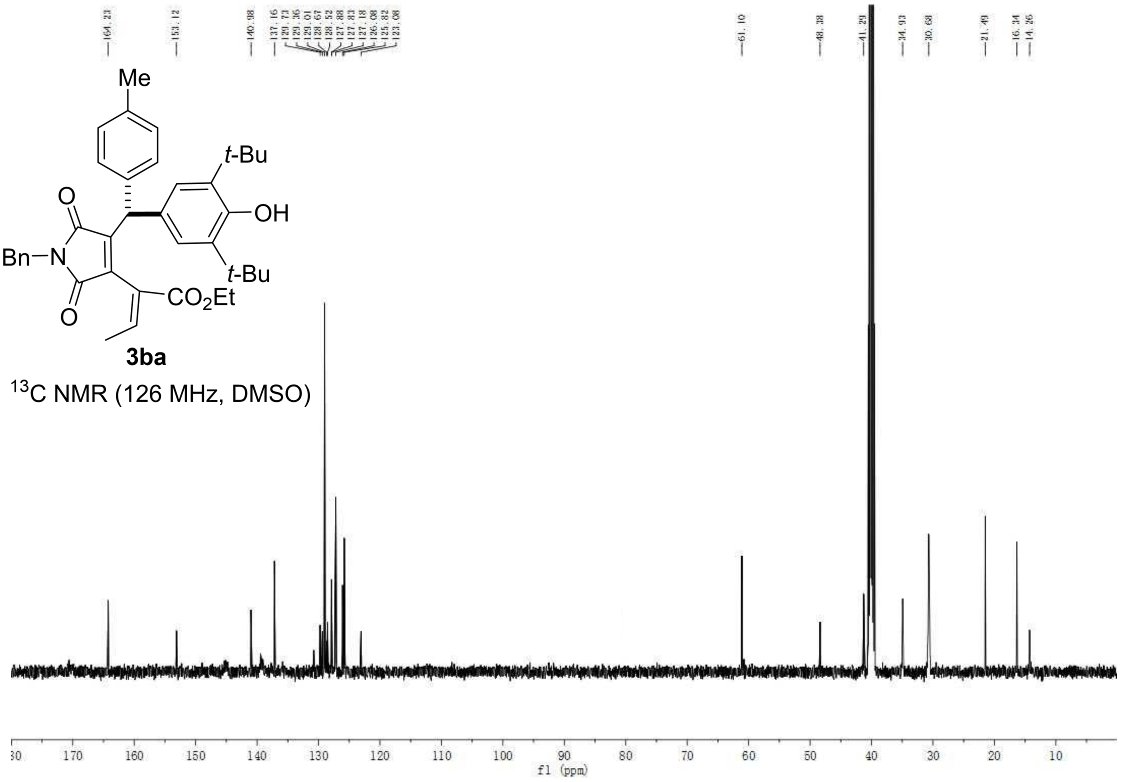

3ba

^{13}C NMR (126 MHz, DMSO)

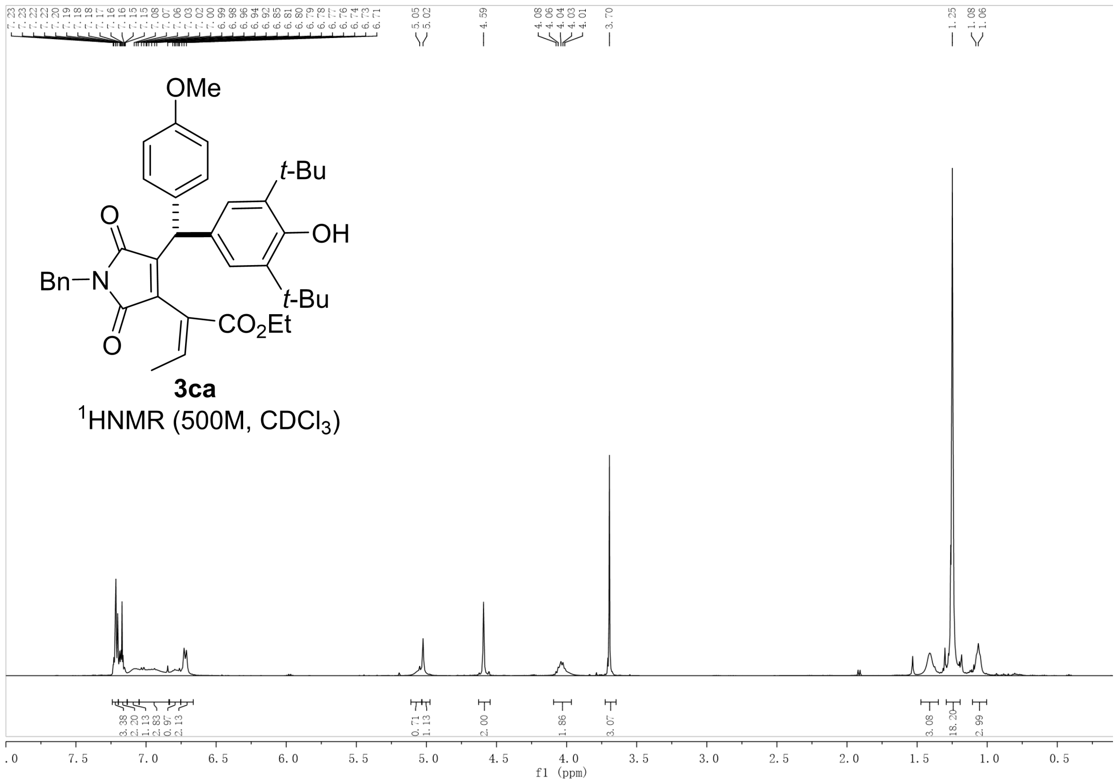
3ca
^{1}HNMR (500M, CDCl$_3$)
f1 (ppm)

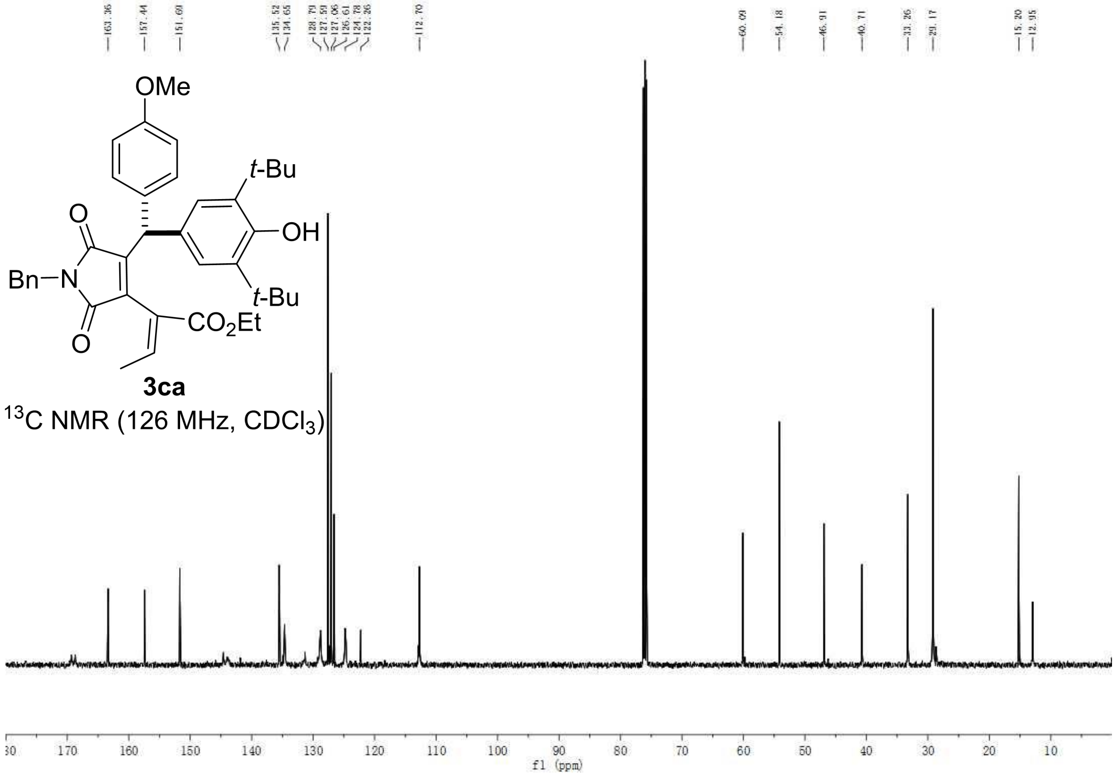
3ca
^{13}C NMR (126 MHz, CDCl$_3$)
f1 (ppm)

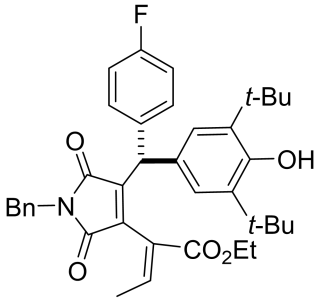

3da

^{1}HNMR (500M, $CDCl_3$)

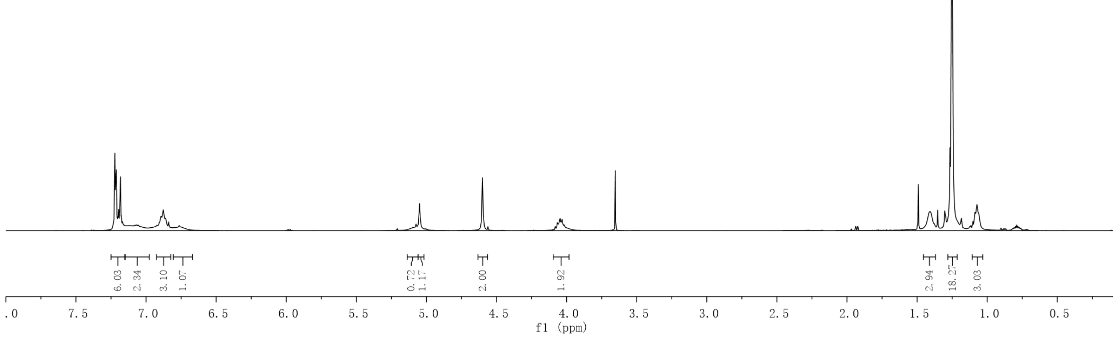

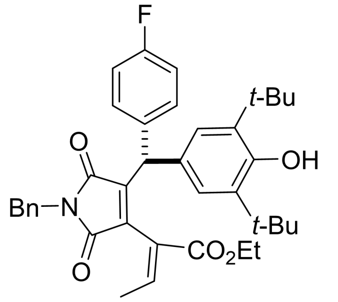

3da

^{13}C NMR (126 MHz, $CDCl_3$)

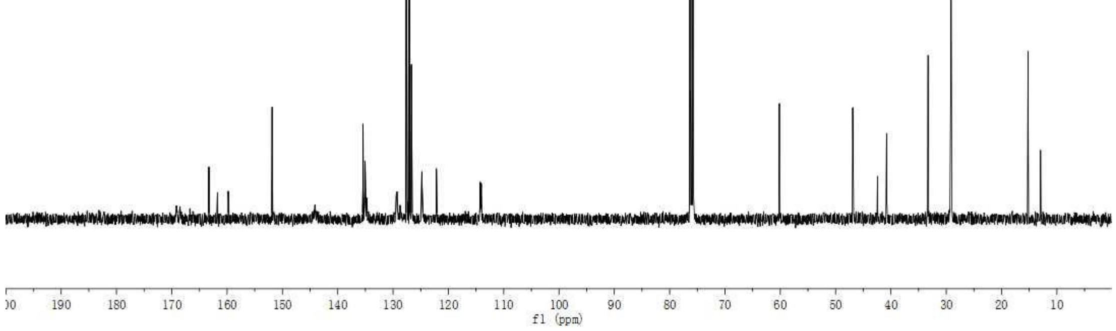

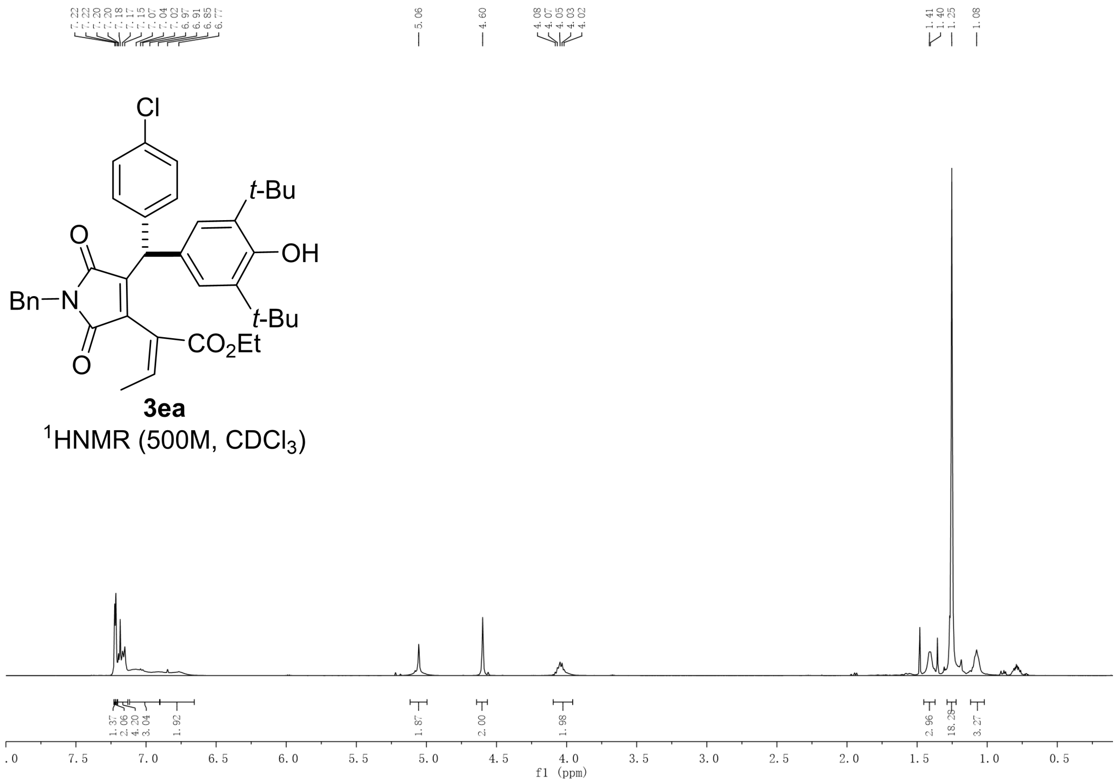
7.22 7.22 7.20 7.20 7.18 7.17 7.15 7.07 7.04 7.02 6.97 6.91 6.85 6.77
5.06
4.60
4.08 4.07 4.05 4.03 4.02
1.41 1.40 1.25
1.08
Cl
t-Bu
OH
t-Bu
O
Bn−N
O
CO2Et
3ea
1HNMR (500M, CDCl3)
1.37 2.06 4.20 3.04 1.92
1.87
2.00
1.98
2.96
18.28
3.27
f1 (ppm)

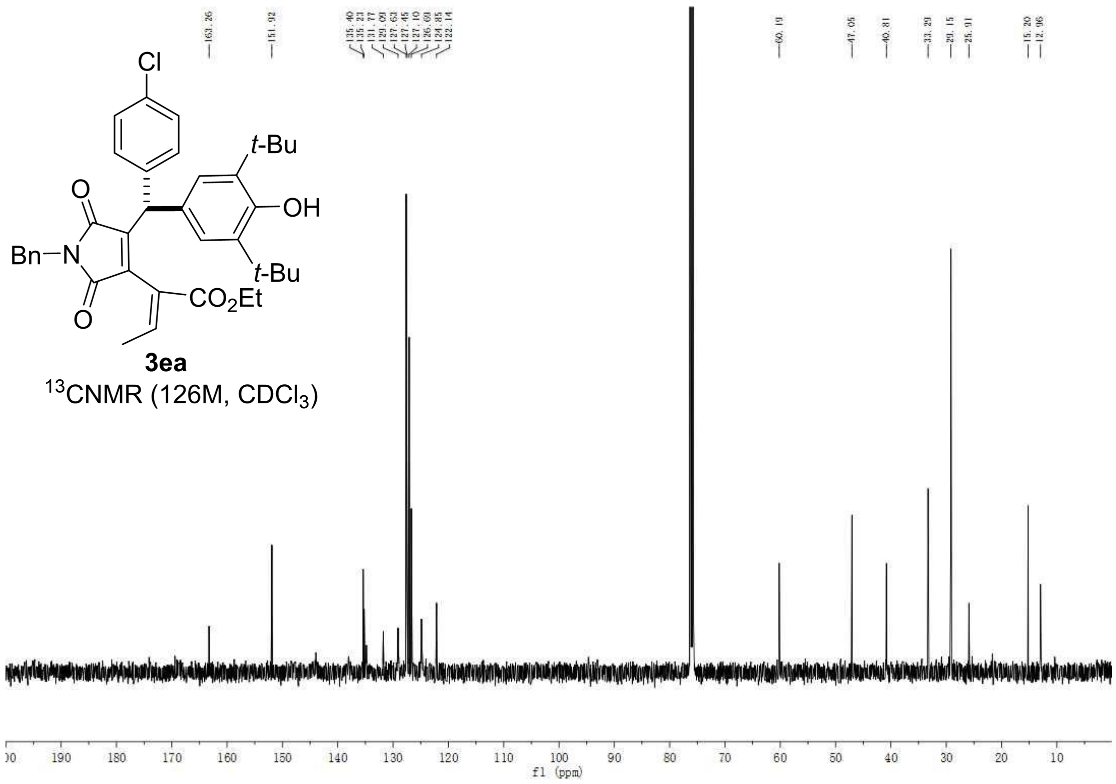
163.26
151.92
135.40 135.23 131.77 129.09 127.63 127.45 127.10 126.69 124.85 122.14
60.19
47.05
40.81
33.29
29.15
25.91
15.20
12.96
Cl
t-Bu
OH
t-Bu
O
Bn−N
O
CO2Et
3ea
13CNMR (126M, CDCl3)
f1 (ppm)

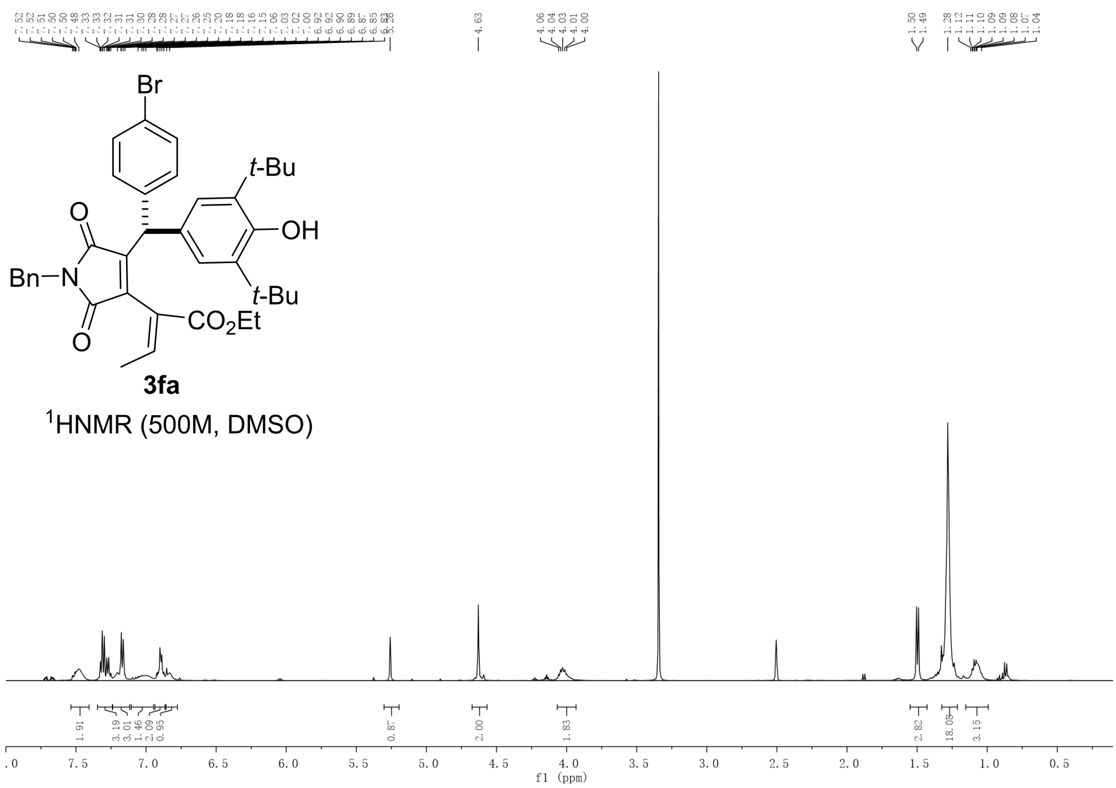
Br
t-Bu
OH
O
Bn−N
t-Bu
O
CO2Et
3fa
1HNMR (500M, DMSO)
f1 (ppm)
7.5 7.0 6.5 6.0 5.5 5.0 4.5 4.0 3.5 3.0 2.5 2.0 1.5 1.0 0.5

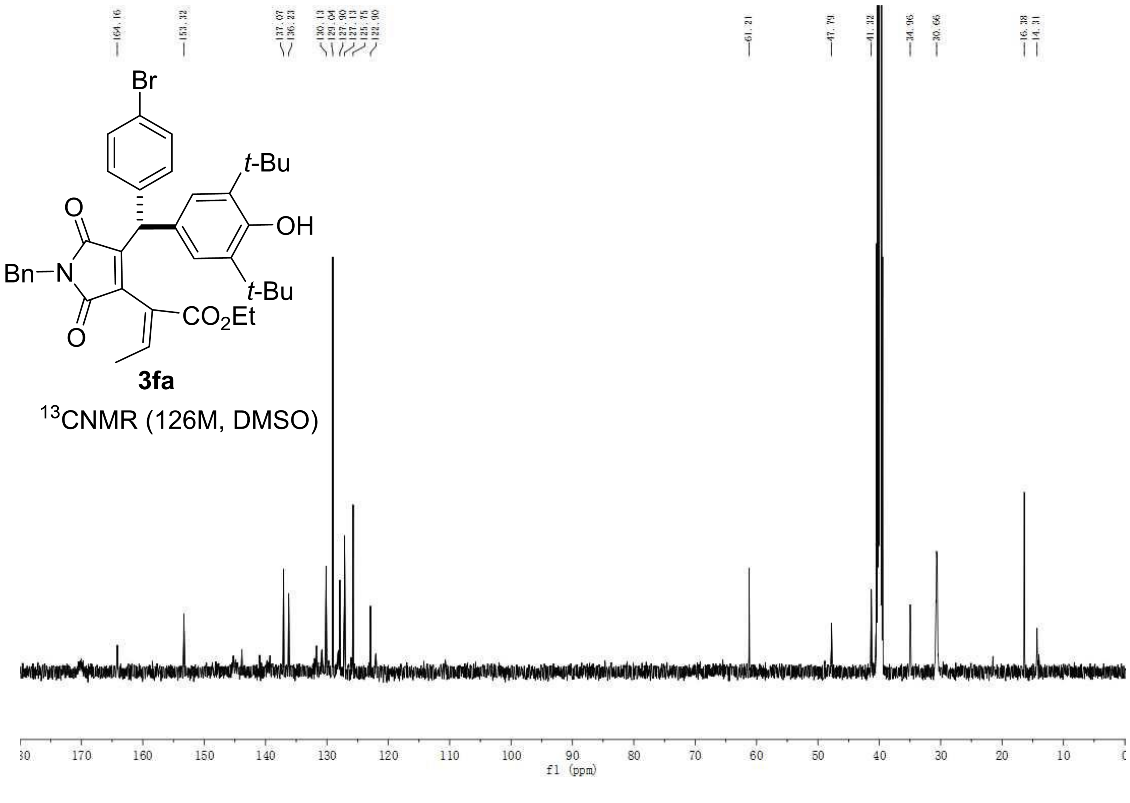
164.16
153.32
137.07
136.23
130.13
129.04
127.90
127.13
125.75
122.90
61.21
47.79
41.32
34.96
30.66
16.38
14.31
Br
t-Bu
OH
O
Bn−N
t-Bu
O
CO2Et
3fa
13CNMR (126M, DMSO)
f1 (ppm)
170 160 150 140 130 120 110 100 90 80 70 60 50 40 30 20 10

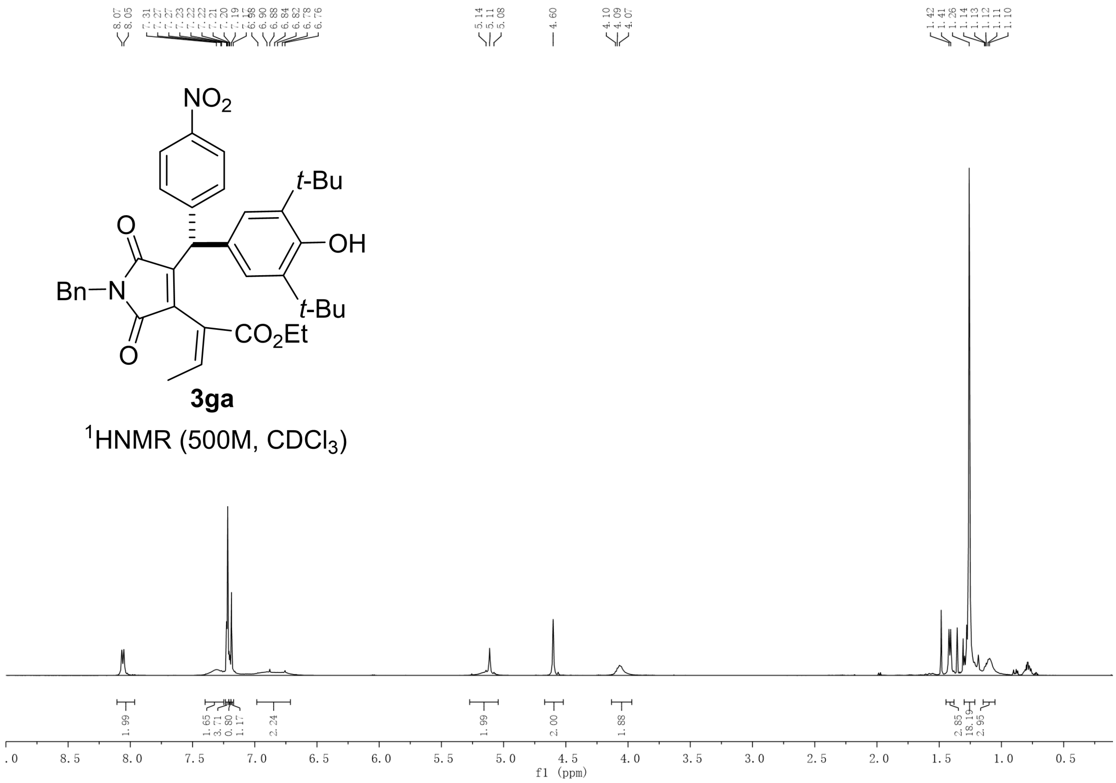

3ga

^{1}HNMR (500M, $CDCl_3$)

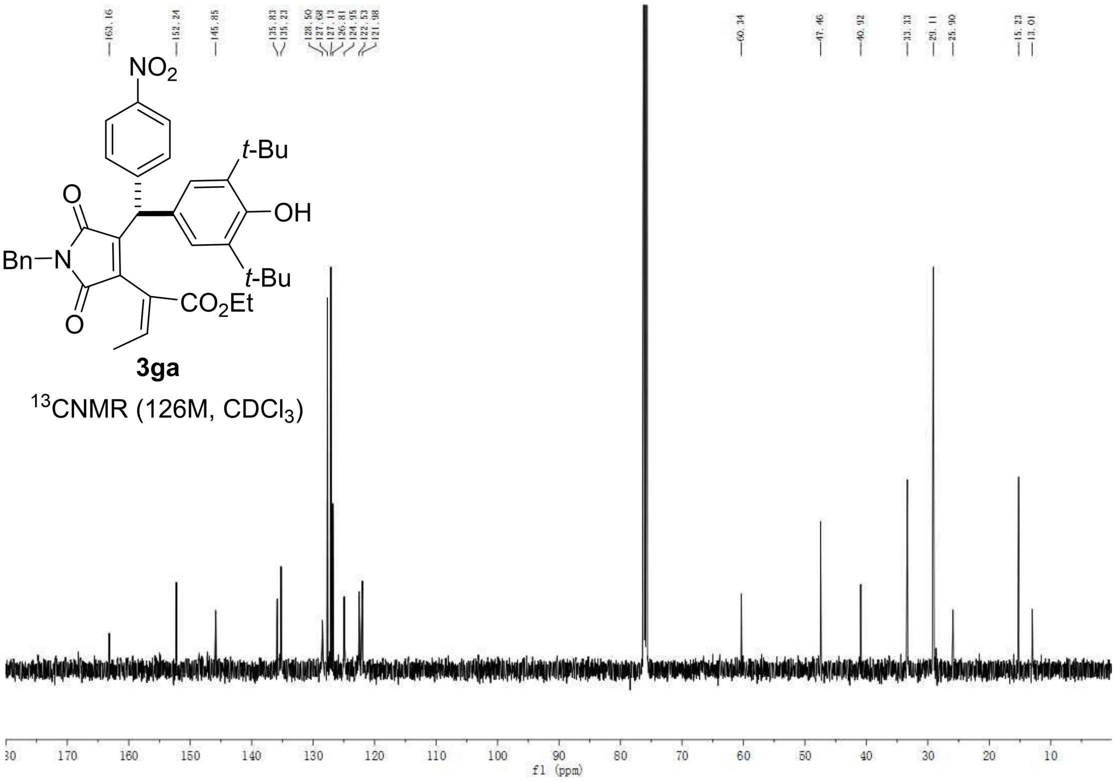

3ga

^{13}CNMR (126M, $CDCl_3$)

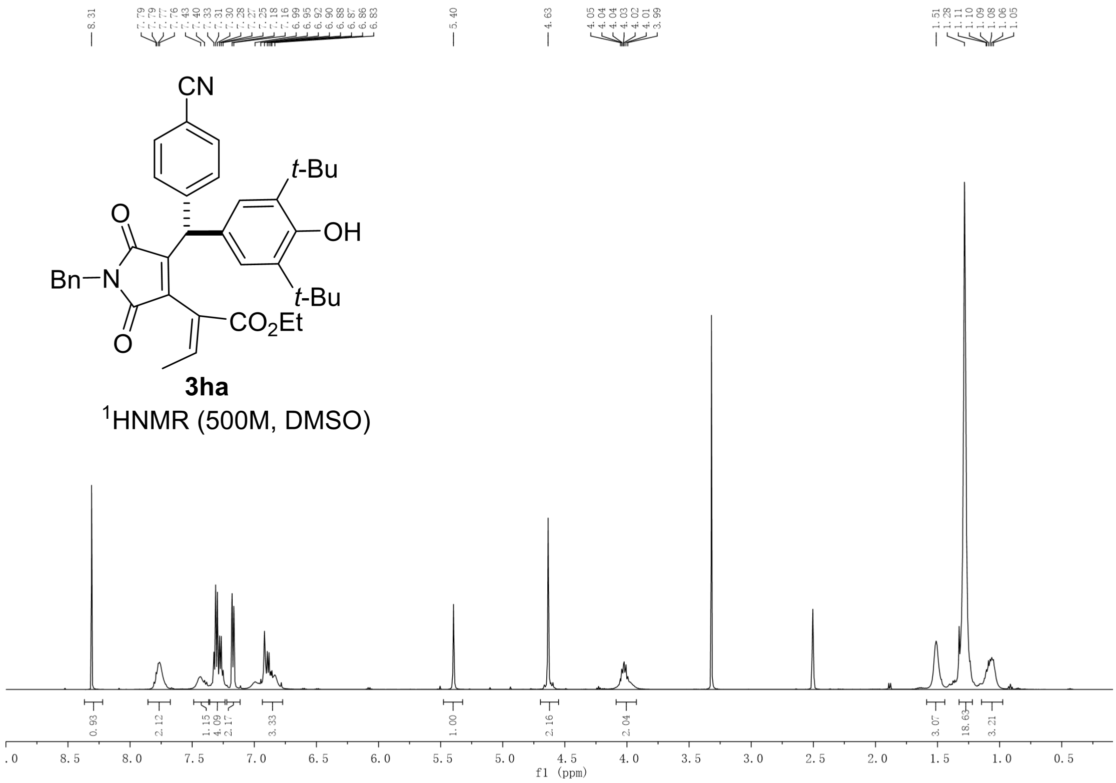

3ha

^{1}HNMR (500M, DMSO)

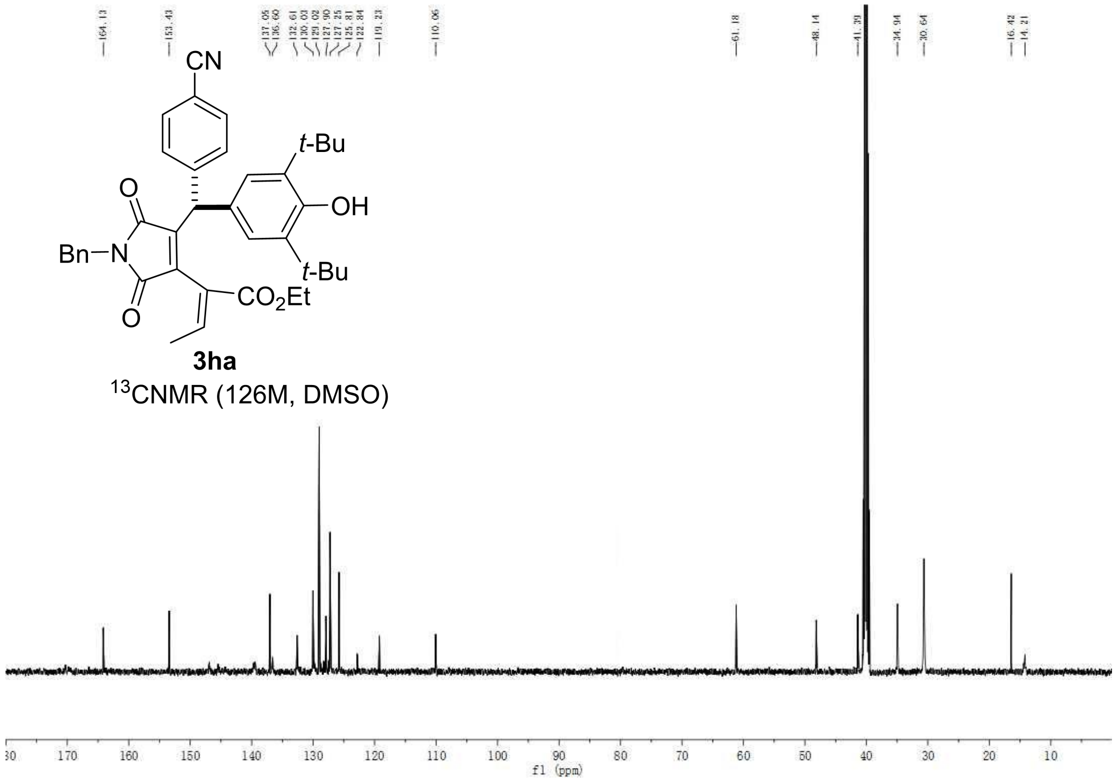

3ha

^{13}CNMR (126M, DMSO)

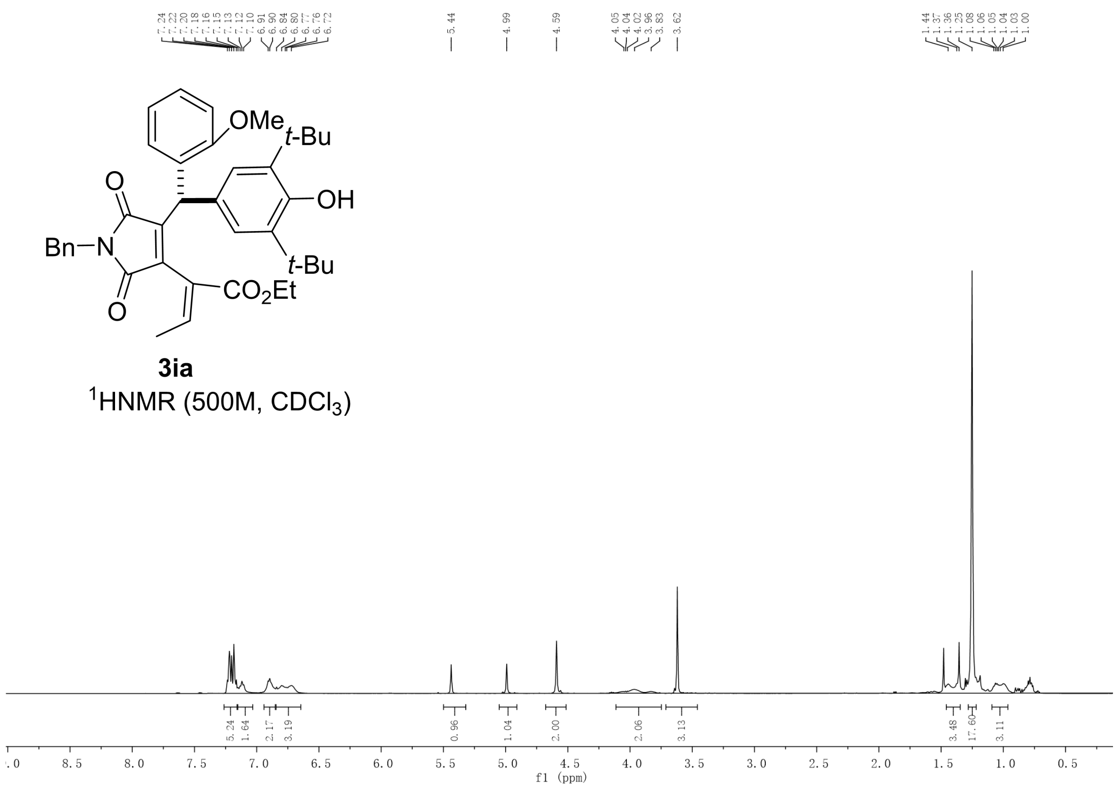

OMe
t-Bu
OH
Bn–N
t-Bu
CO2Et
3ia
1HNMR (500M, CDCl3)
f1 (ppm)

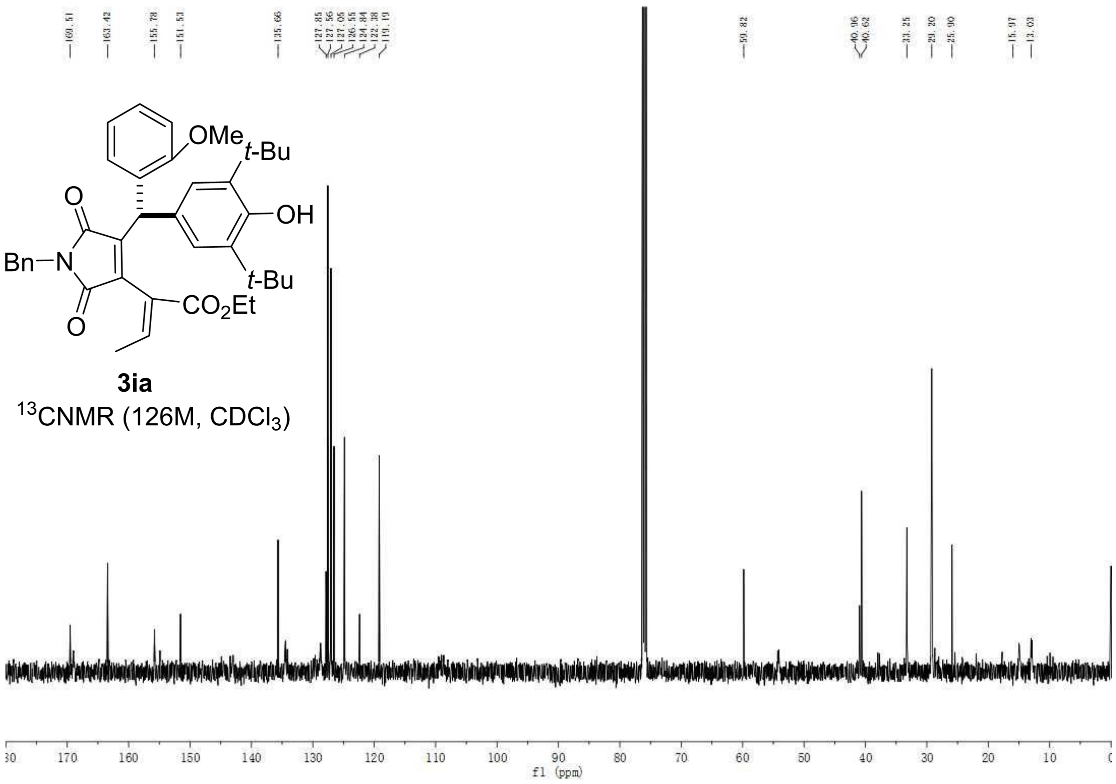

OMe
t-Bu
OH
Bn–N
t-Bu
CO2Et
3ia
13CNMR (126M, CDCl3)
f1 (ppm)

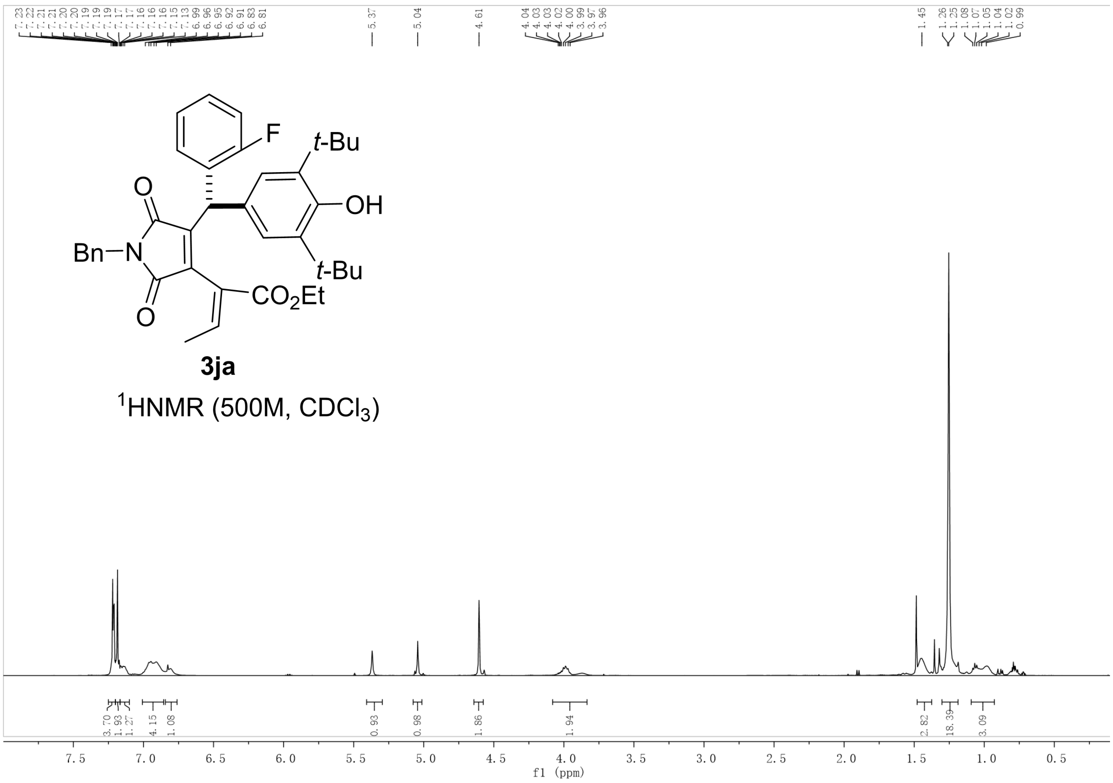

F
t-Bu
O
OH
Bn−N
t-Bu
CO_2Et
O
3ja
^{1}HNMR (500M, $CDCl_3$)
f1 (ppm)

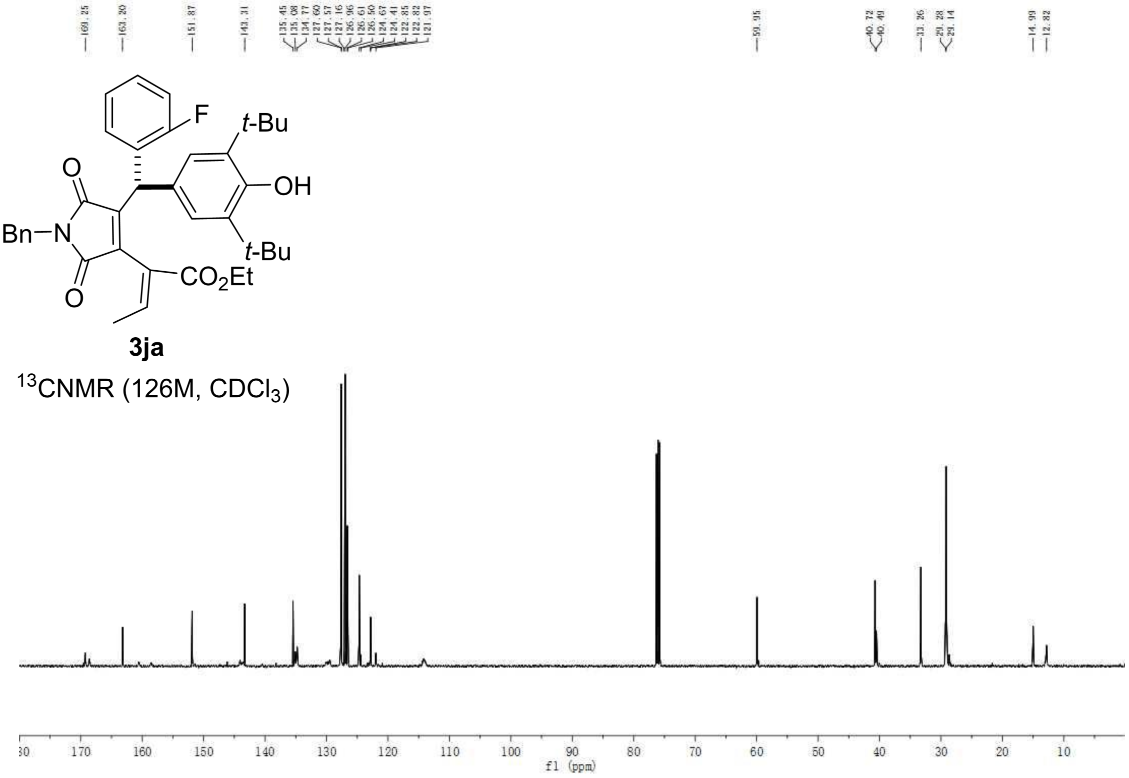

F
t-Bu
O
OH
Bn−N
t-Bu
CO_2Et
O
3ja
^{13}CNMR (126M, $CDCl_3$)
f1 (ppm)

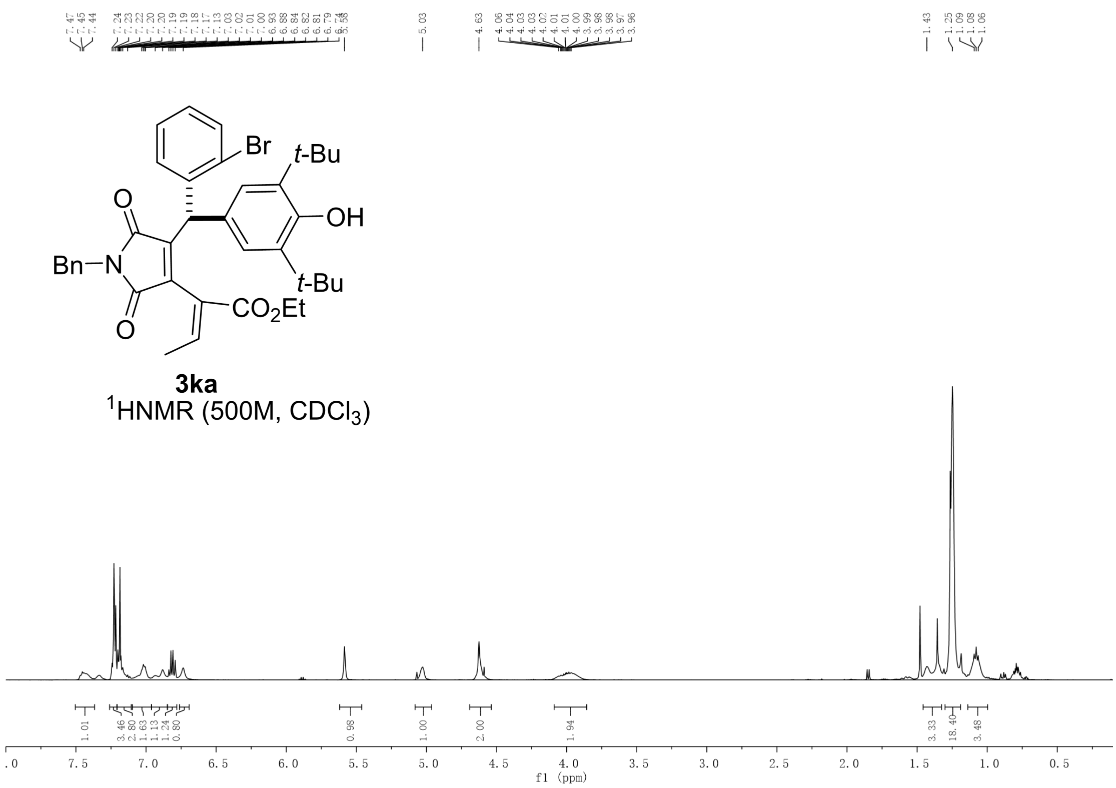

Br
t-Bu
OH
t-Bu
O
Bn−N
O
CO2Et
3ka
1HNMR (500M, CDCl3)
f1 (ppm)

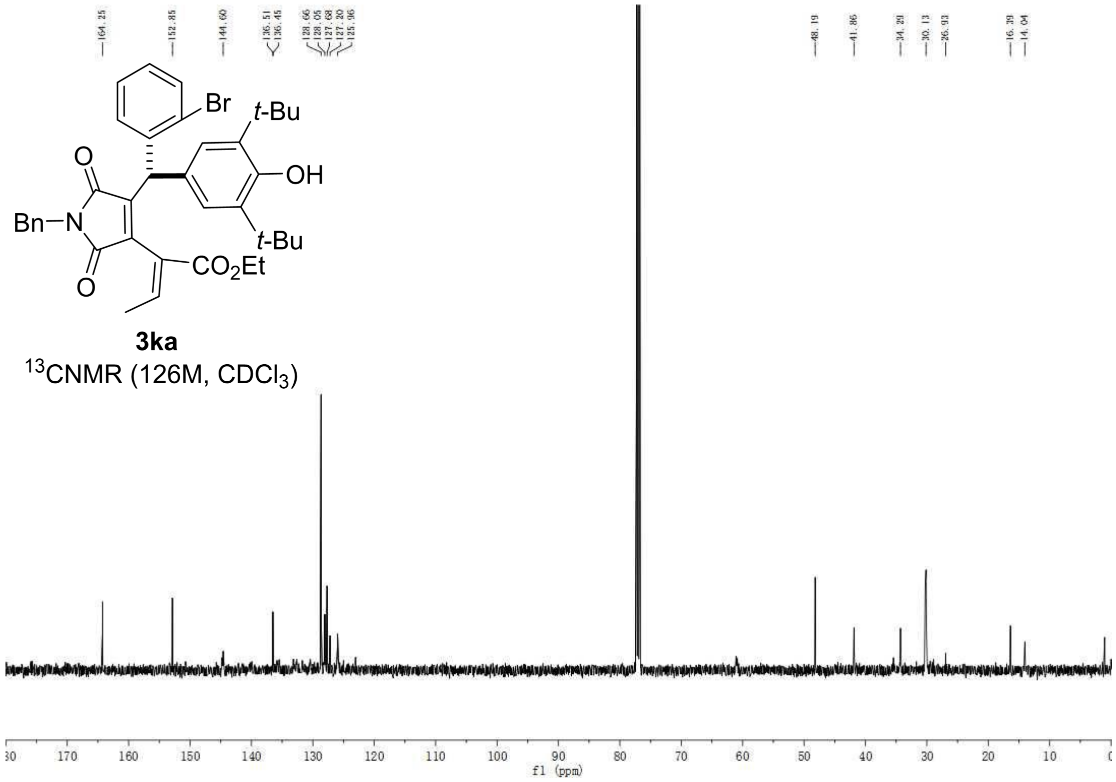

Br
t-Bu
OH
t-Bu
O
Bn−N
O
CO2Et
3ka
13CNMR (126M, CDCl3)
f1 (ppm)

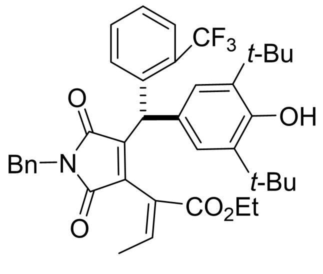

3la

^{1}HNMR (500M, $CDCl_3$)

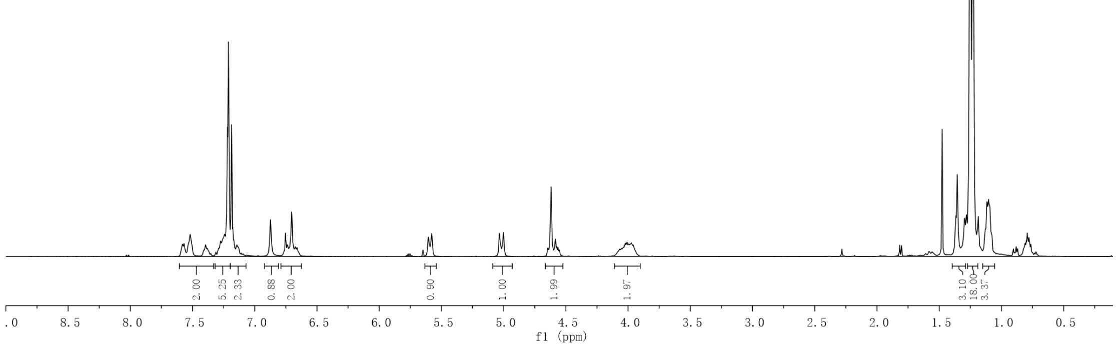

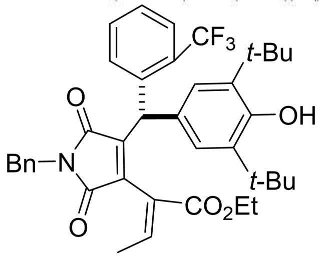

3la

^{13}CNMR (126M, $CDCl_3$)

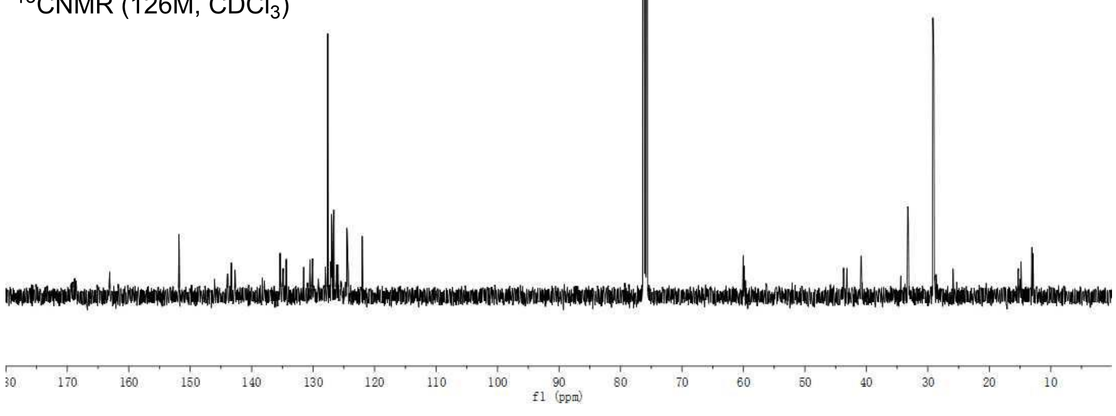

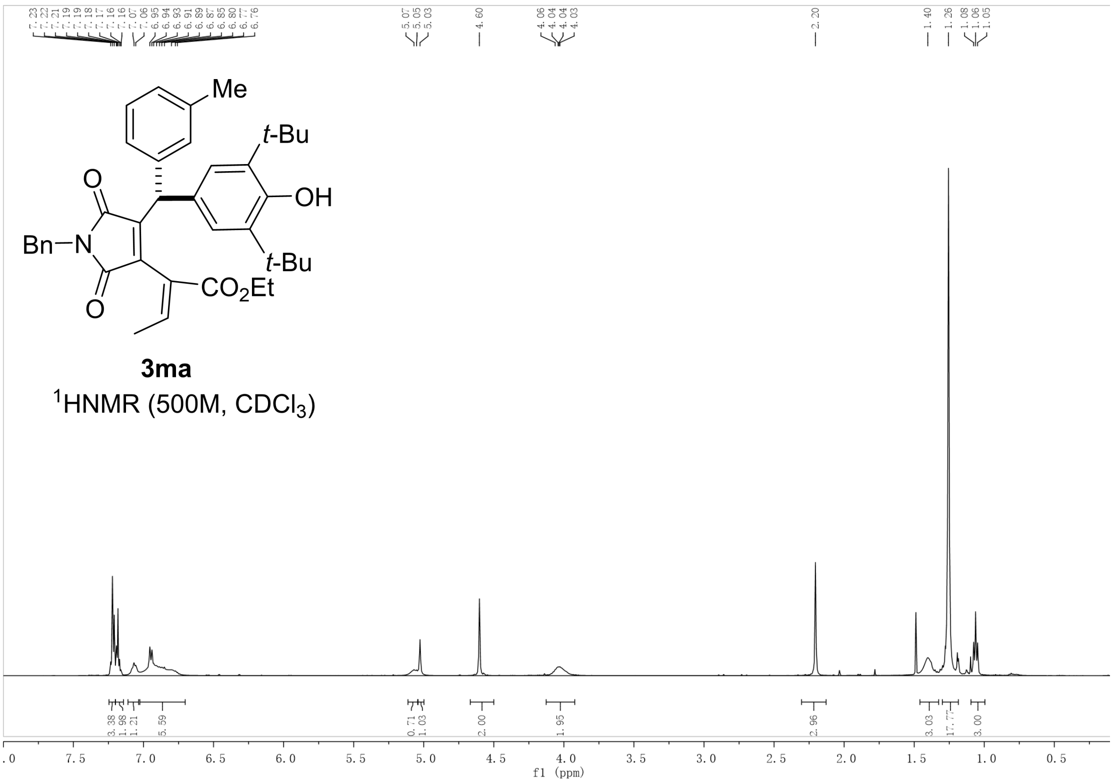
3ma
1HNMR (500M, CDCl3)
f1 (ppm)

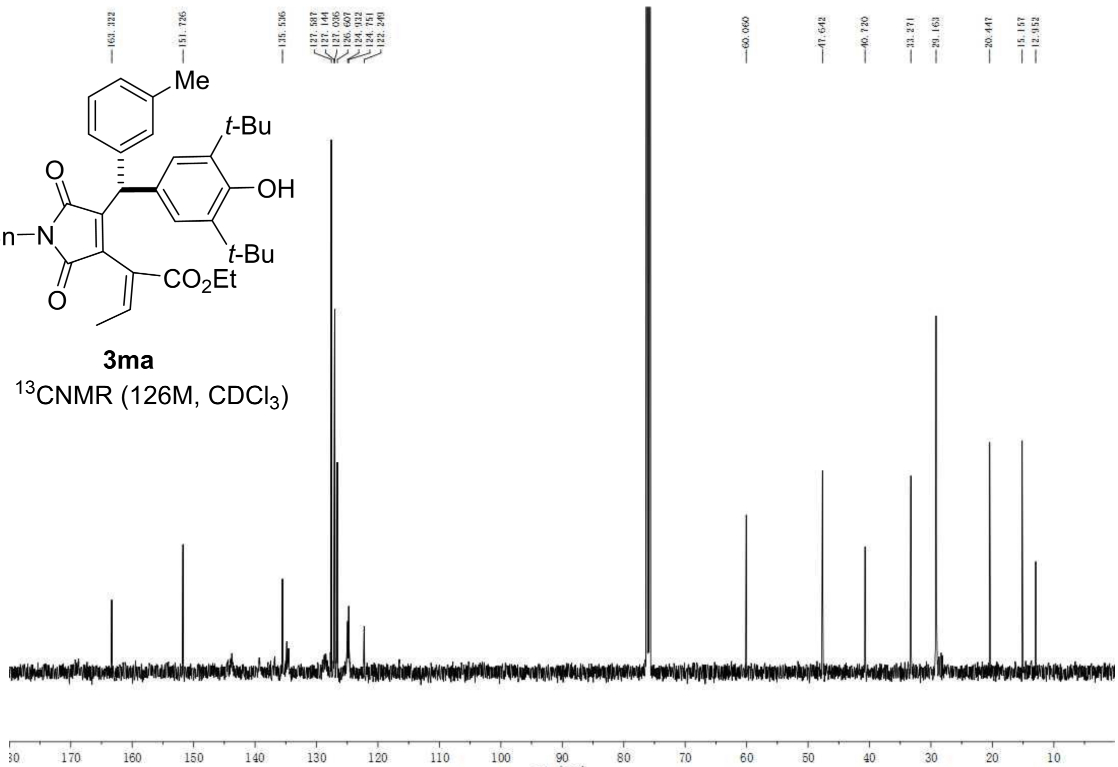
3ma
13CNMR (126M, CDCl3)
f1 (ppm)

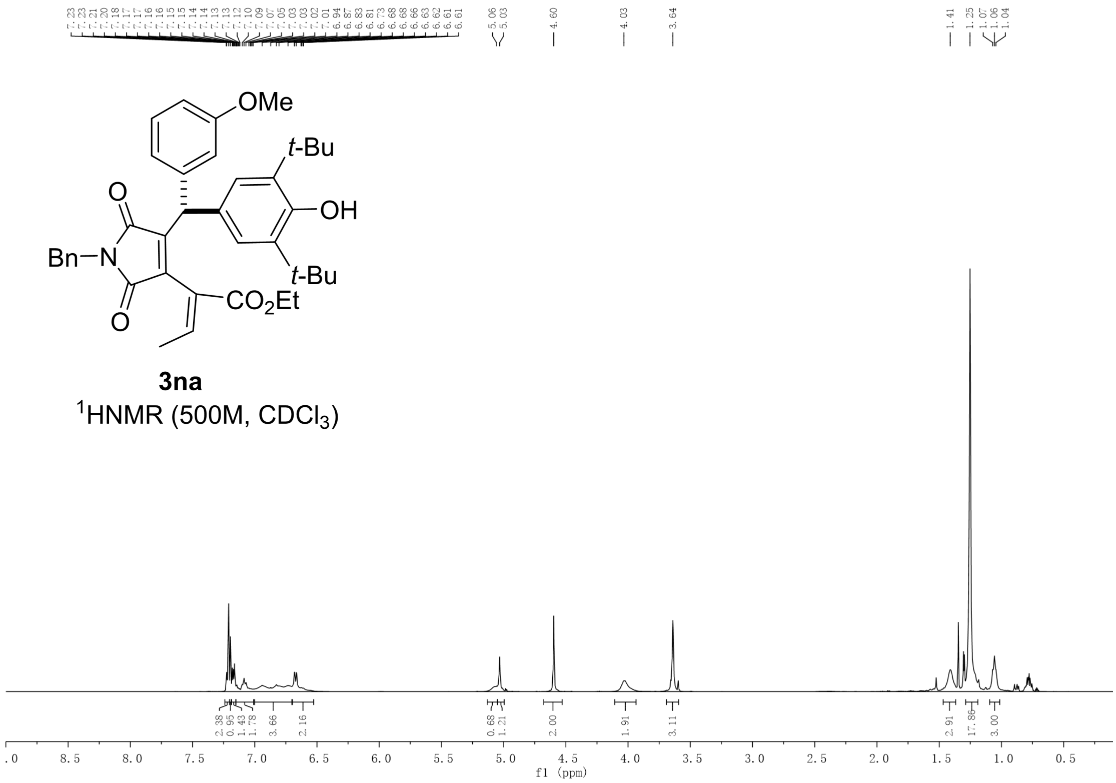
OMe
t-Bu
OH
t-Bu
Bn—N
CO2Et
3na
1HNMR (500M, CDCl3)
f1 (ppm)

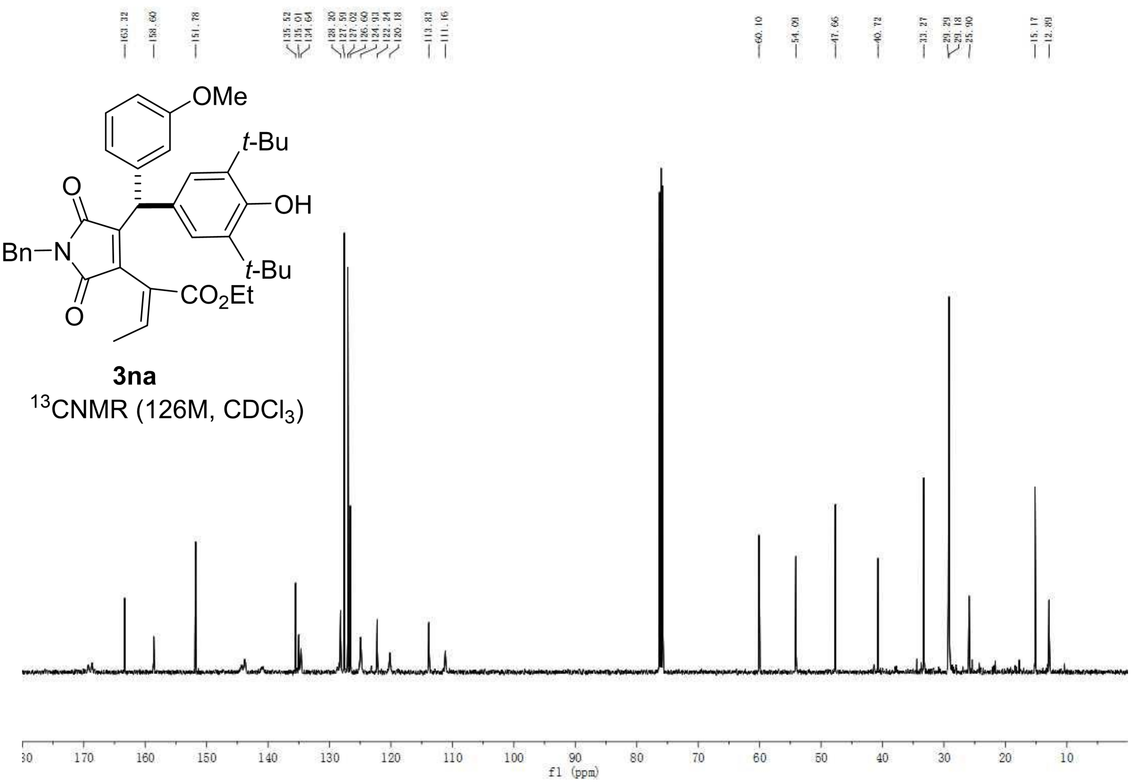
OMe
t-Bu
OH
t-Bu
Bn—N
CO2Et
3na
13CNMR (126M, CDCl3)
f1 (ppm)

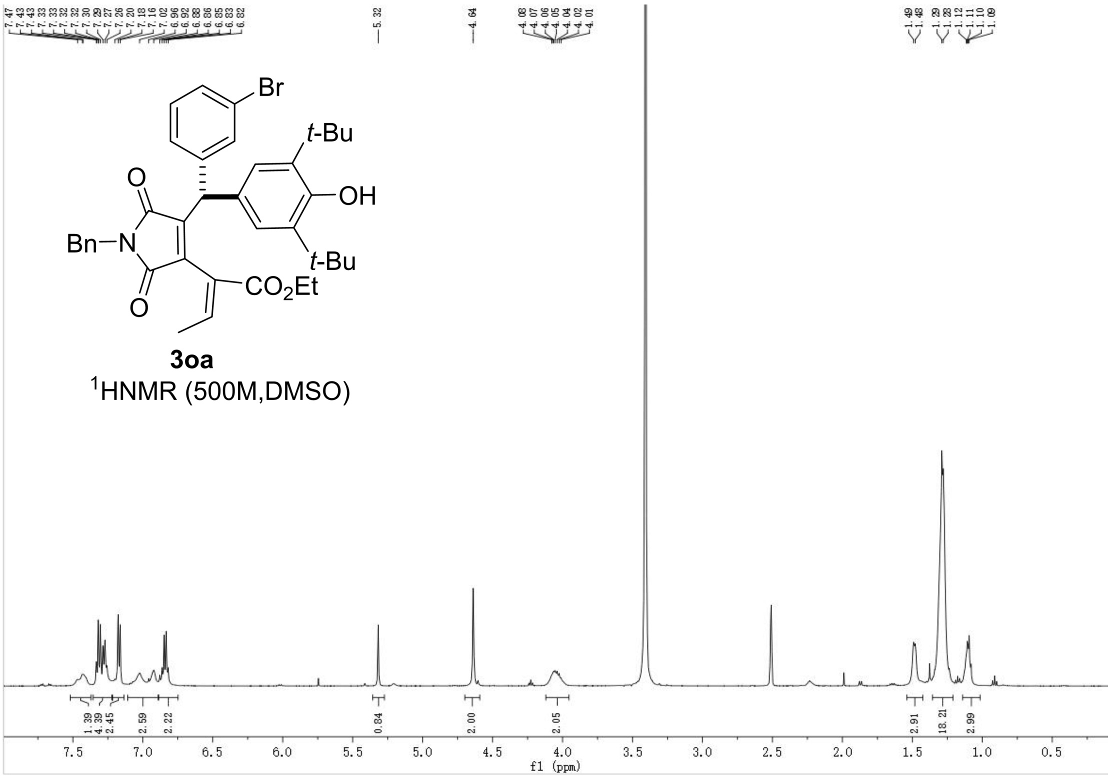

3oa

^{1}HNMR (500M,DMSO)

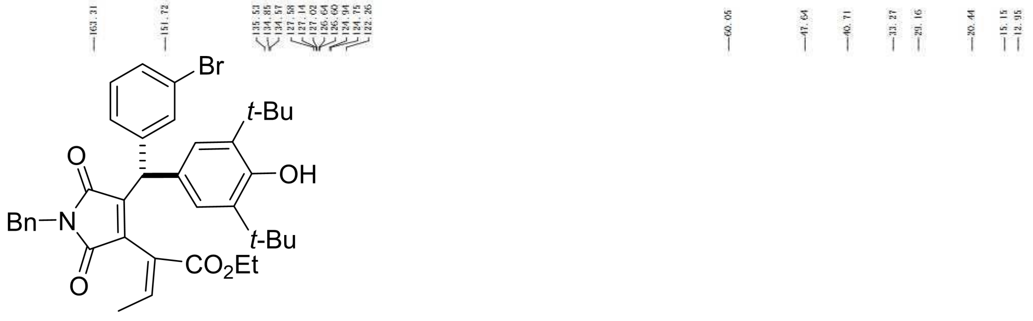

3oa

^{13}CNMR (500M, $CDCl_3$)

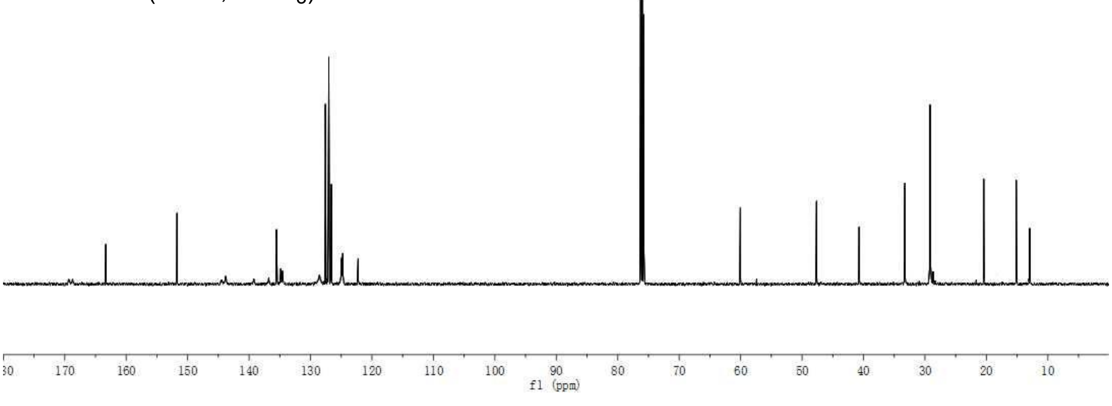

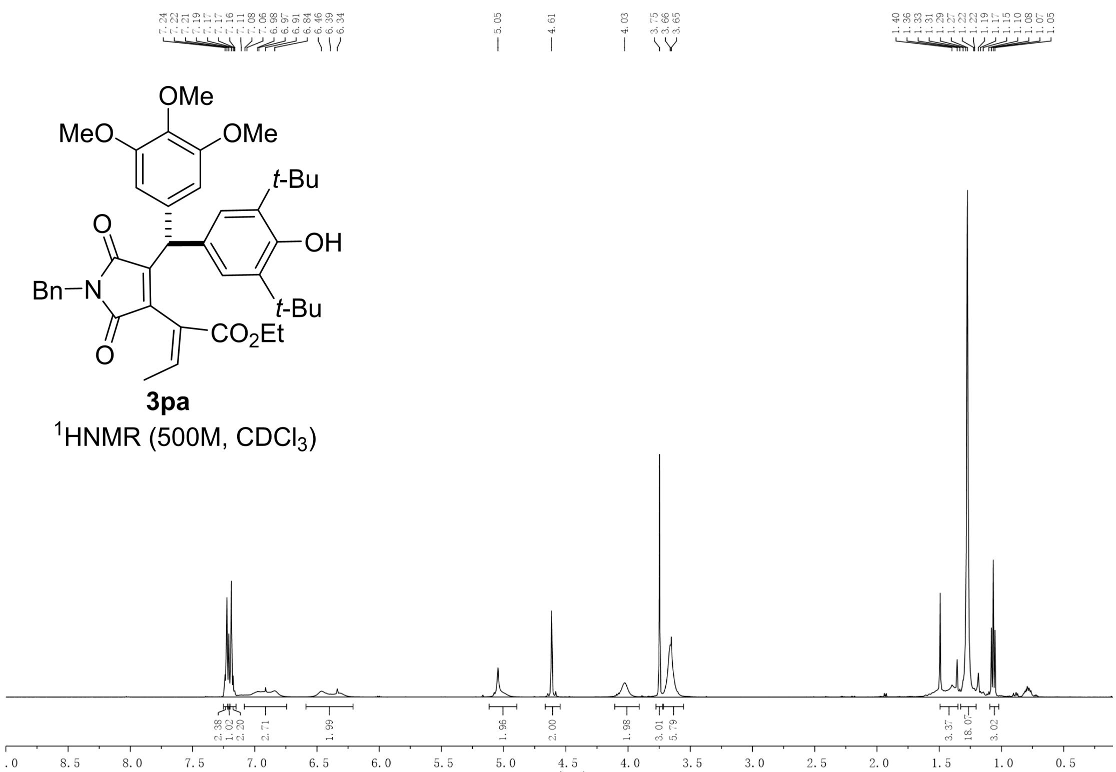
OMe
MeO
OMe
t-Bu
OH
Bn−N
t-Bu
CO2Et
3pa
1HNMR (500M, CDCl3)
f1 (ppm)

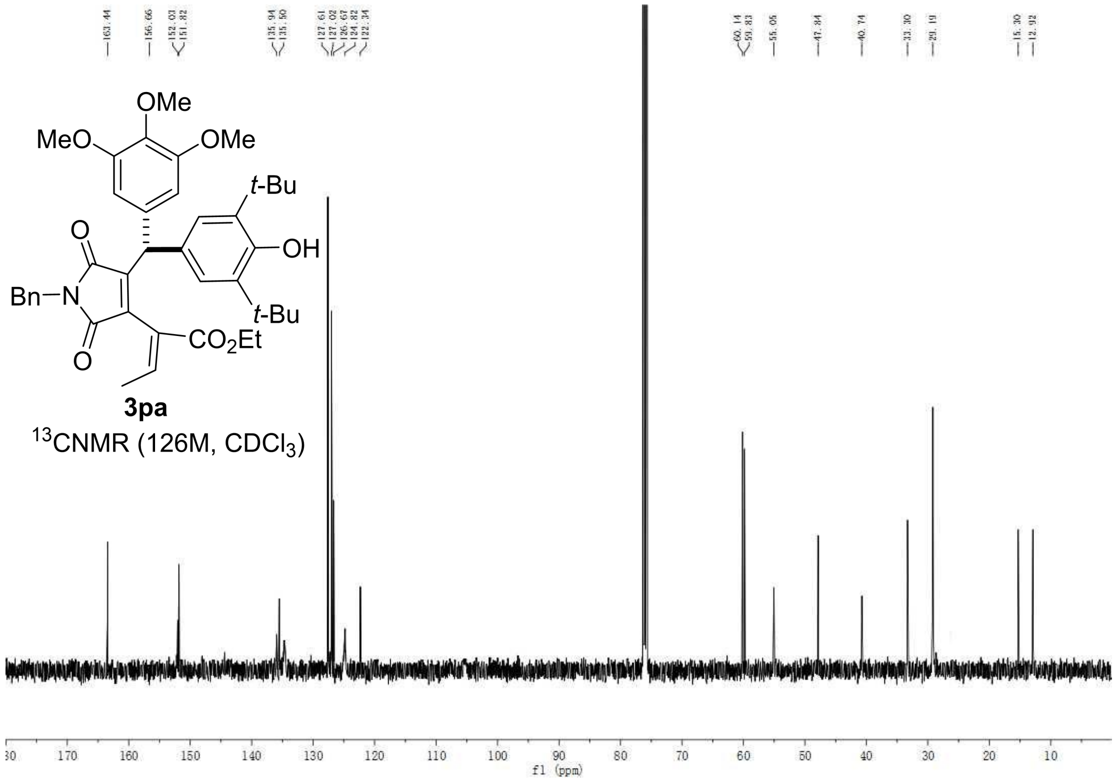
OMe
MeO
OMe
t-Bu
OH
Bn−N
t-Bu
CO2Et
3pa
13CNMR (126M, CDCl3)
f1 (ppm)

3qa

^{1}HNMR (500M, $CDCl_3$)

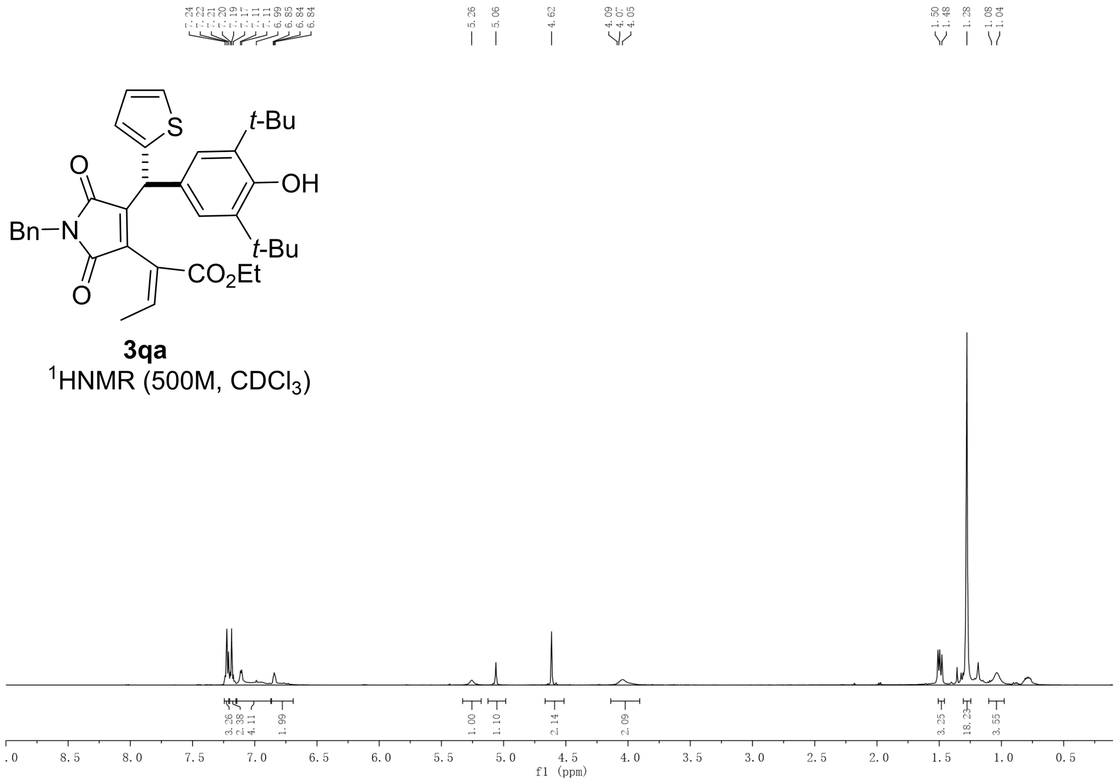

3qa

^{13}CNMR (126M, $CDCl_3$)

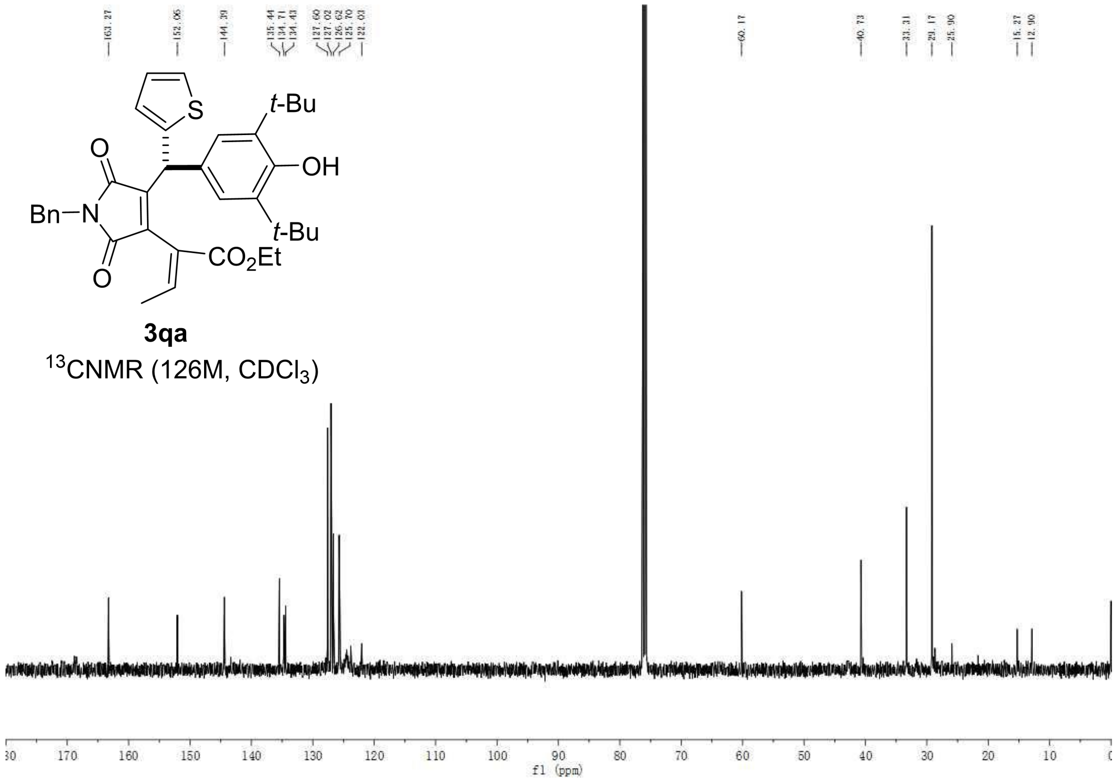

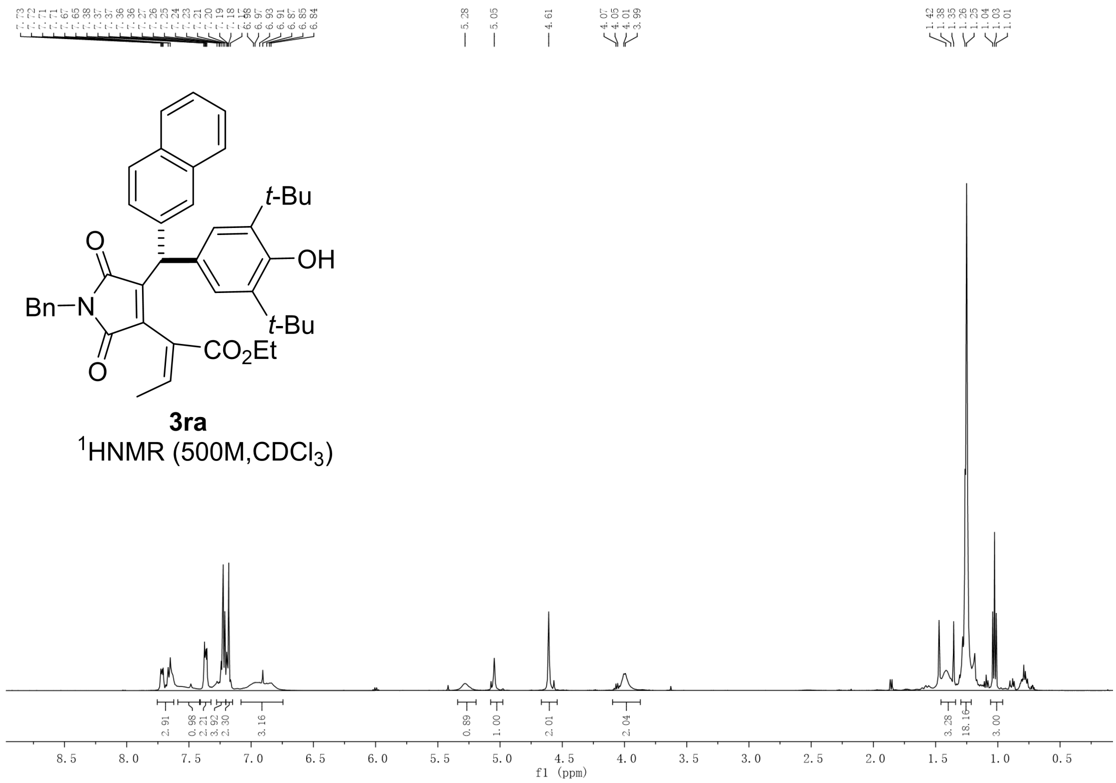
7.73 7.72 7.71 7.71 7.67 7.65 7.38 7.37 7.37 7.36 7.36 7.27 7.26 7.25 7.24 7.23 7.21 7.20 7.19 7.18 7.17 6.98 6.97 6.93 6.91 6.87 6.85 6.84
5.28
5.05
4.61
4.07 4.05 4.01 3.99
1.42 1.38 1.35 1.26 1.25 1.04 1.03 1.01
t-Bu
OH
t-Bu
Bn–N
O
O
CO_2Et
3ra
^{1}HNMR (500M,CDCl$_3$)
2.91 0.98 2.21 3.92 2.30 3.16 0.89 1.00 2.01 2.04 3.28 18.16 3.00
8.5 8.0 7.5 7.0 6.5 6.0 5.5 5.0 4.5 4.0 3.5 3.0 2.5 2.0 1.5 1.0 0.5
f1 (ppm)

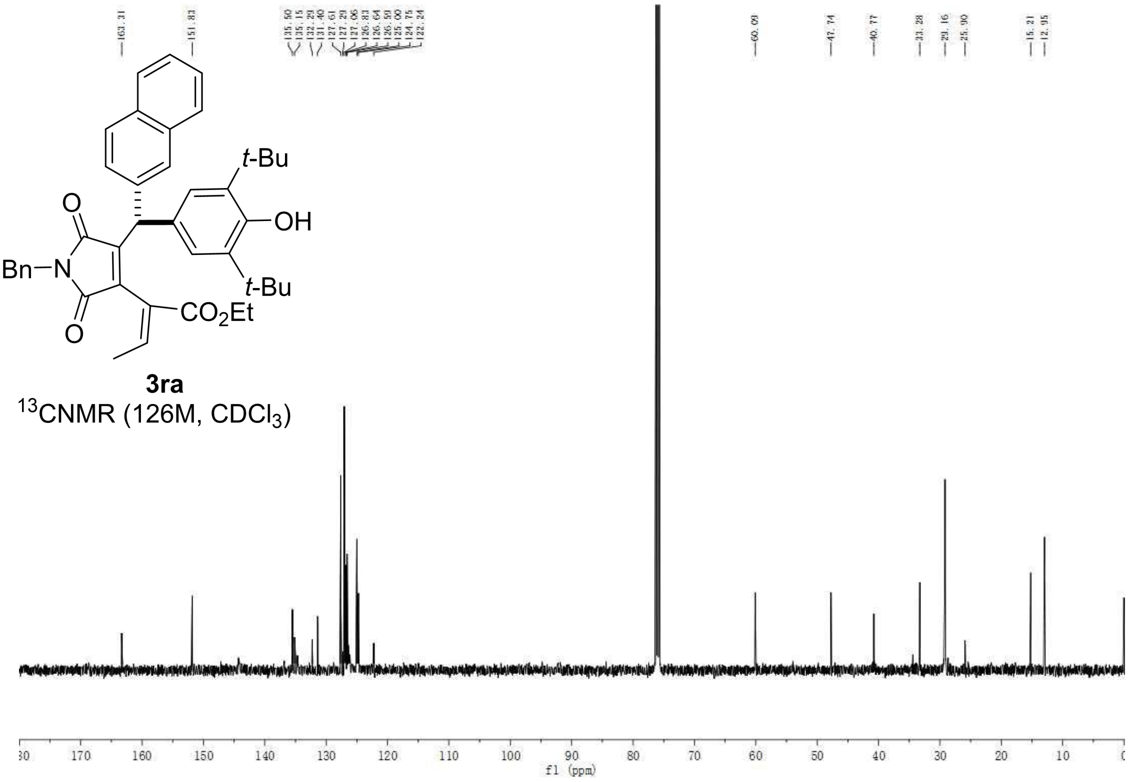
163.31
151.83
135.50 135.15 132.29 131.40 127.61 127.29 127.06 126.83 126.64 126.59 125.00 124.75 122.24
60.09
47.74
40.77
33.28
29.16
25.90
15.21
12.95
t-Bu
OH
t-Bu
Bn–N
O
O
CO_2Et
3ra
^{13}CNMR (126M, CDCl$_3$)
170 160 150 140 130 120 110 100 90 80 70 60 50 40 30 20 10
f1 (ppm)

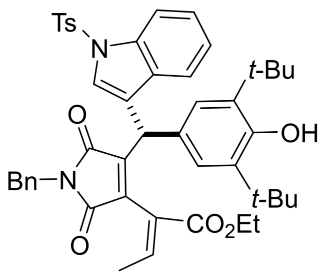

3sa

^{1}HNMR (500M, $CDCl_3$)

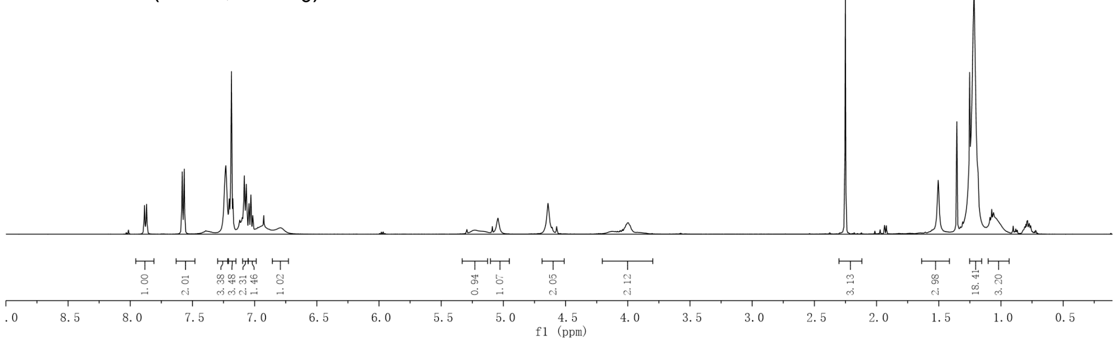

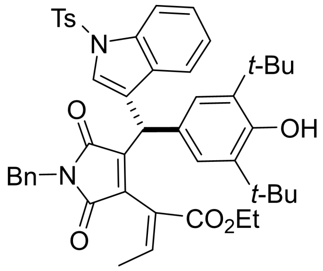

3sa

^{13}CNMR (126M, $CDCl_3$)

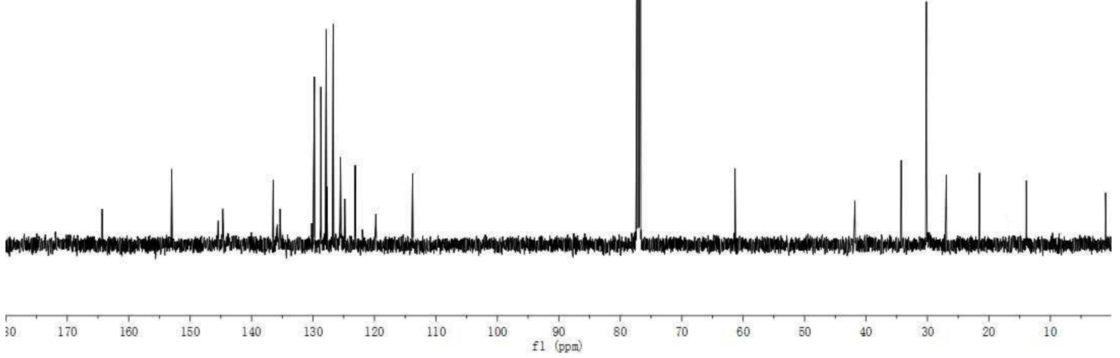

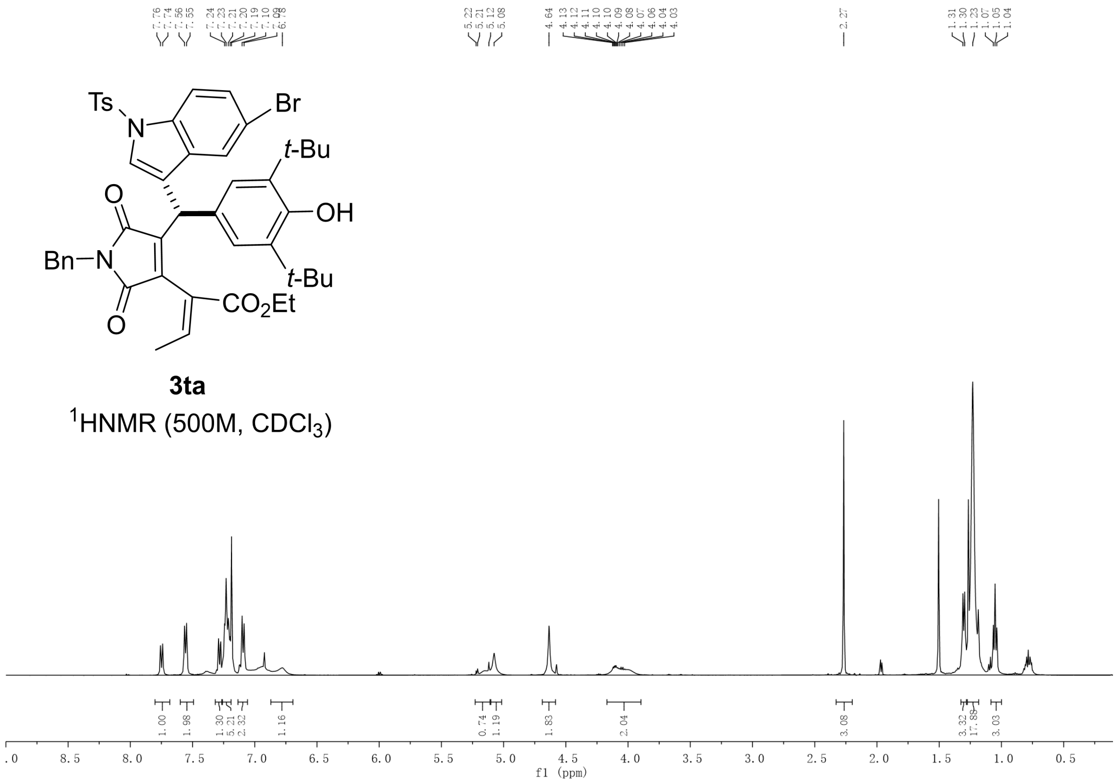
Ts
N
Br
t-Bu
OH
t-Bu
O
Bn–N
O
CO2Et
3ta
1HNMR (500M, CDCl3)
f1 (ppm)

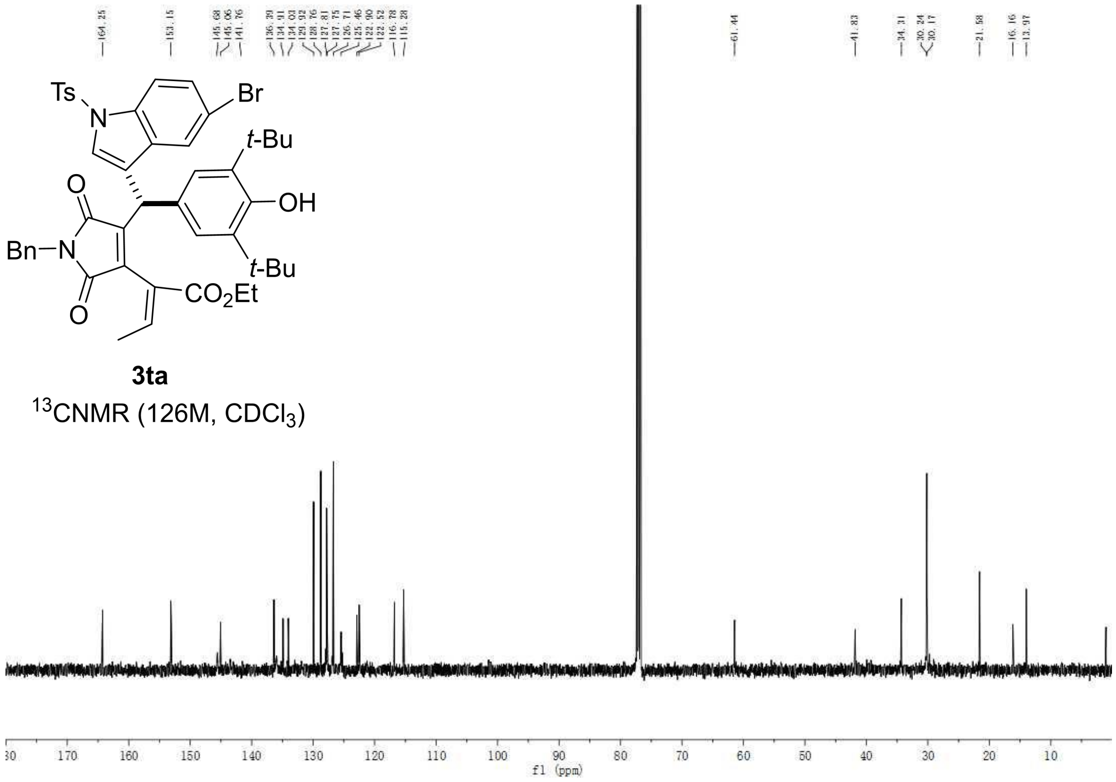
Ts
N
Br
t-Bu
OH
t-Bu
O
Bn–N
O
CO2Et
3ta
13CNMR (126M, CDCl3)
f1 (ppm)

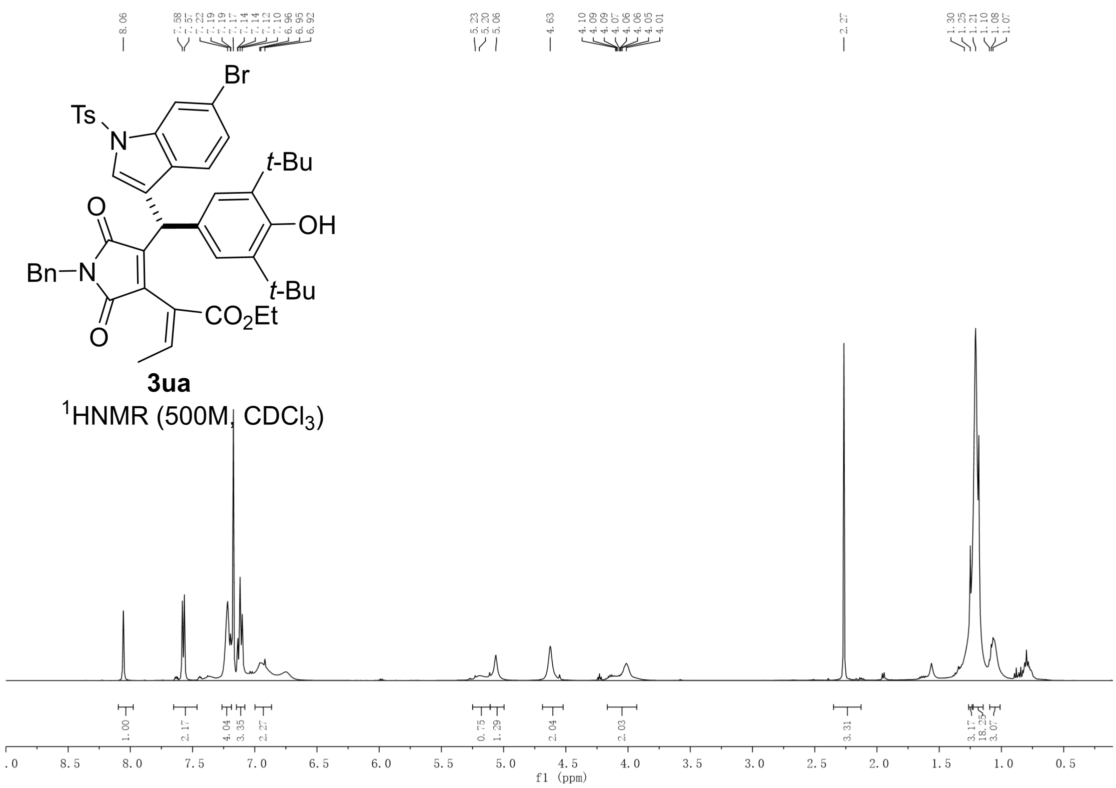

Br
Ts
N
t-Bu
O
OH
Bn−N
t-Bu
CO2Et
O
3ua
1HNMR (500M, CDCl3)
f1 (ppm)

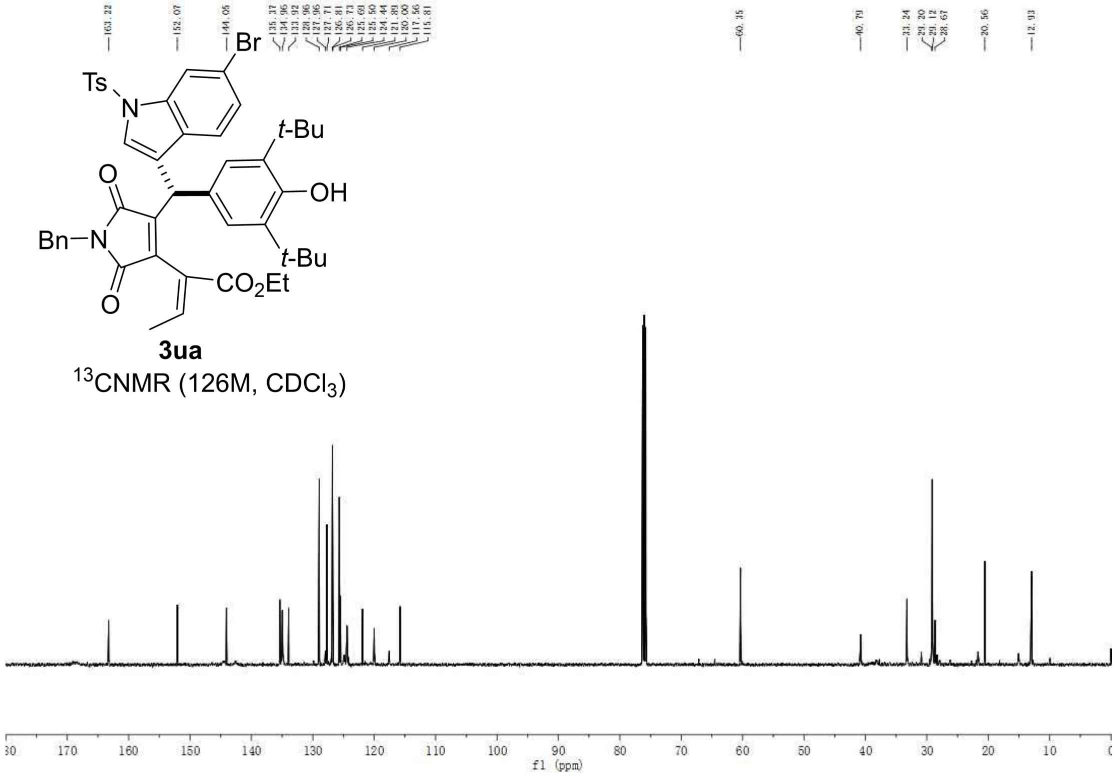

Br
Ts
N
t-Bu
O
OH
Bn−N
t-Bu
CO2Et
O
3ua
13CNMR (126M, CDCl3)
f1 (ppm)

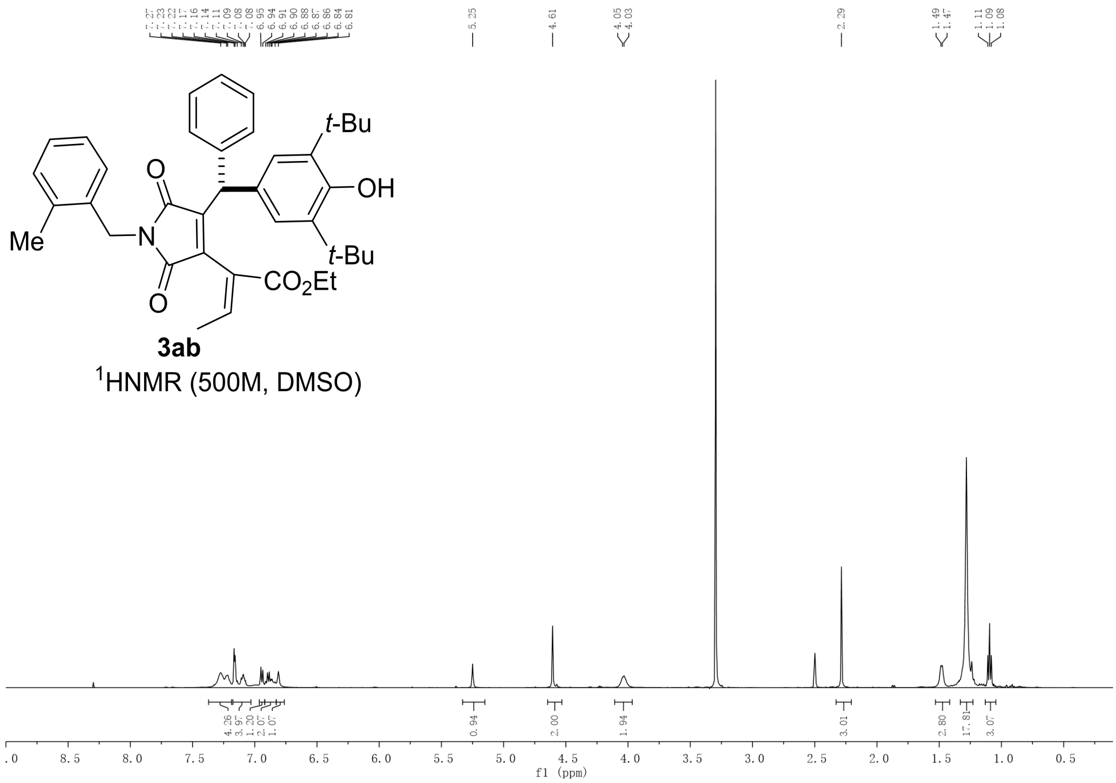

3ab

^{1}HNMR (500M, DMSO)

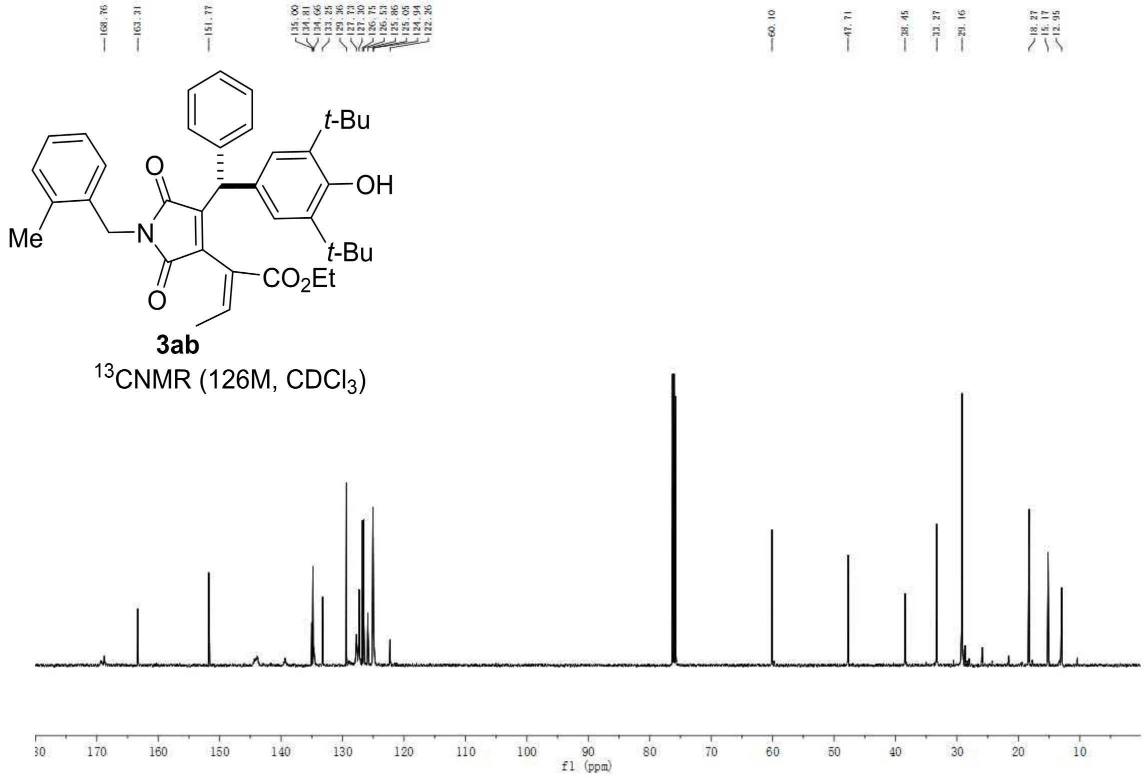

3ab

^{13}CNMR (126M, $CDCl_3$)

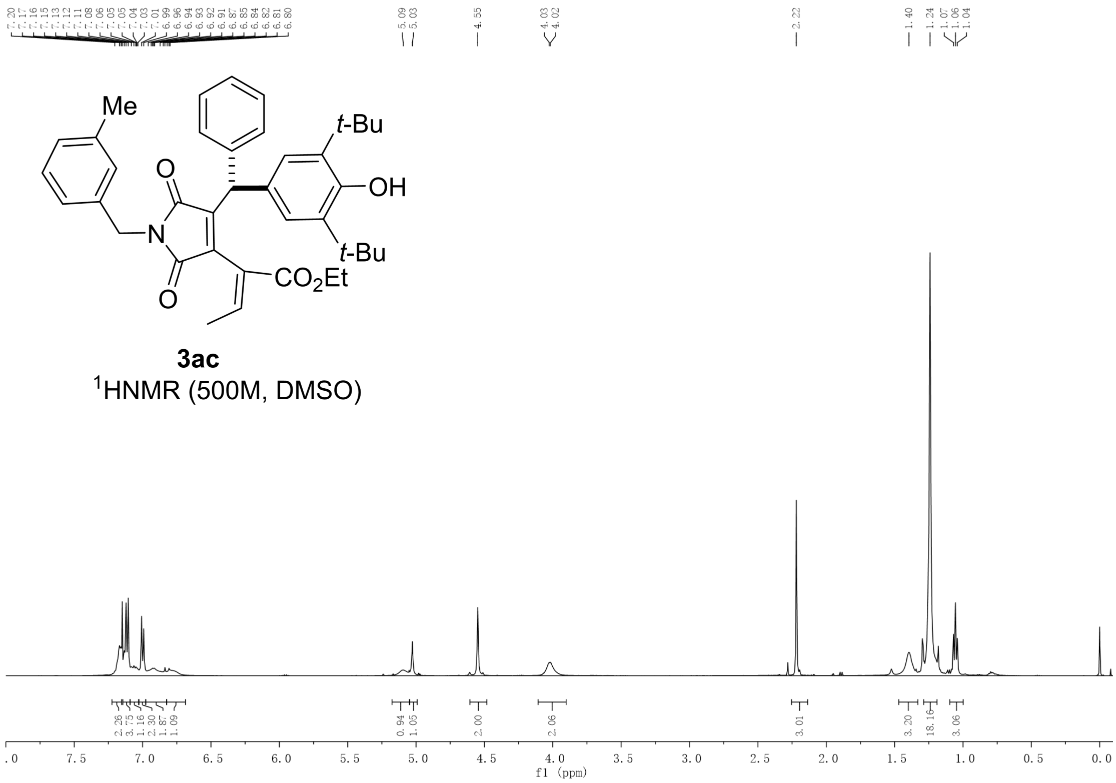
Me
O
N
O
t-Bu
OH
t-Bu
CO2Et
3ac
1HNMR (500M, DMSO)
f1 (ppm)

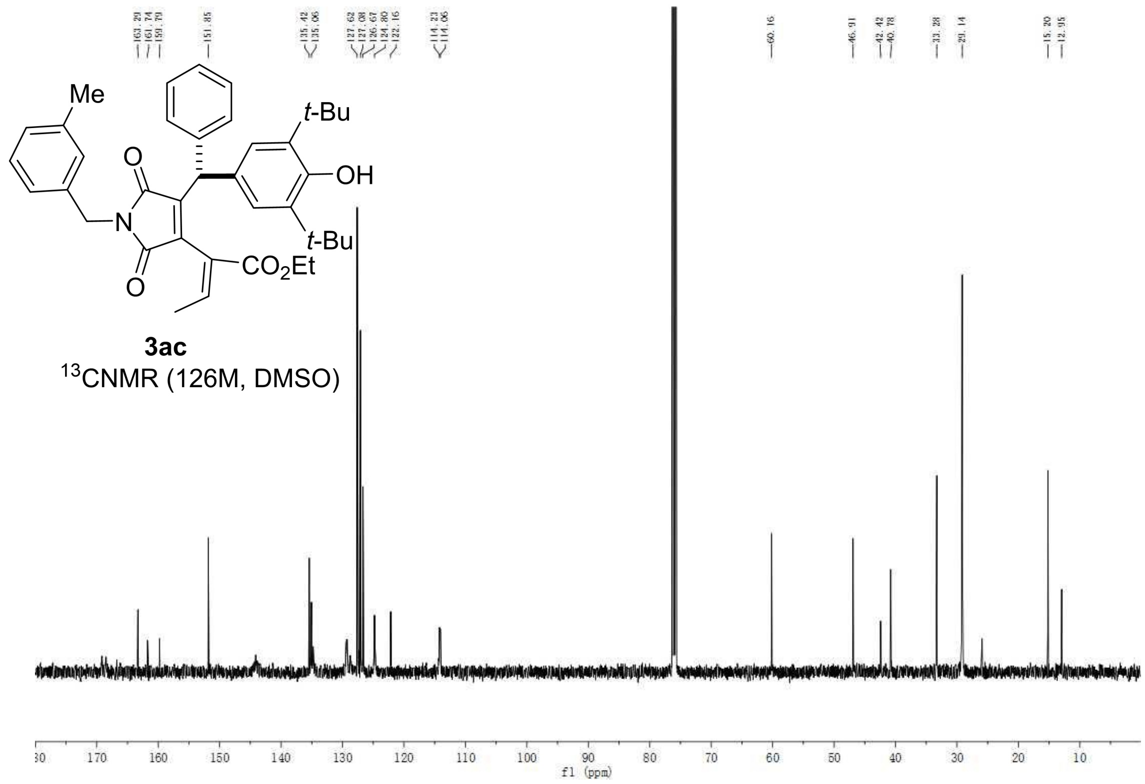
Me
O
N
O
t-Bu
OH
t-Bu
CO2Et
3ac
13CNMR (126M, DMSO)
f1 (ppm)

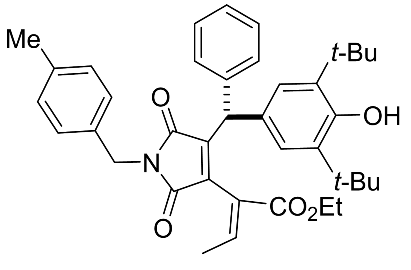

3ad

^{1}HNMR (500M, $CDCl_3$)

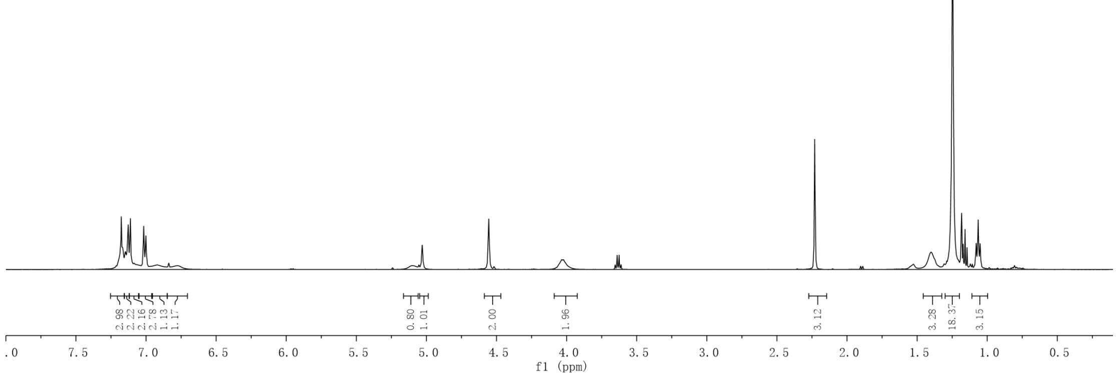

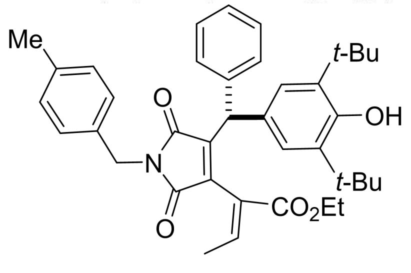

3ad

^{13}CNMR (126M, $CDCl_3$)

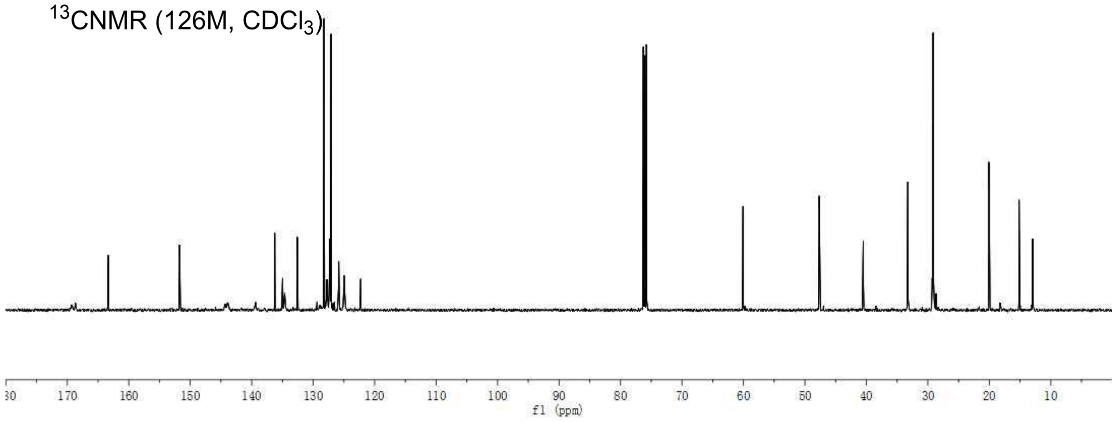

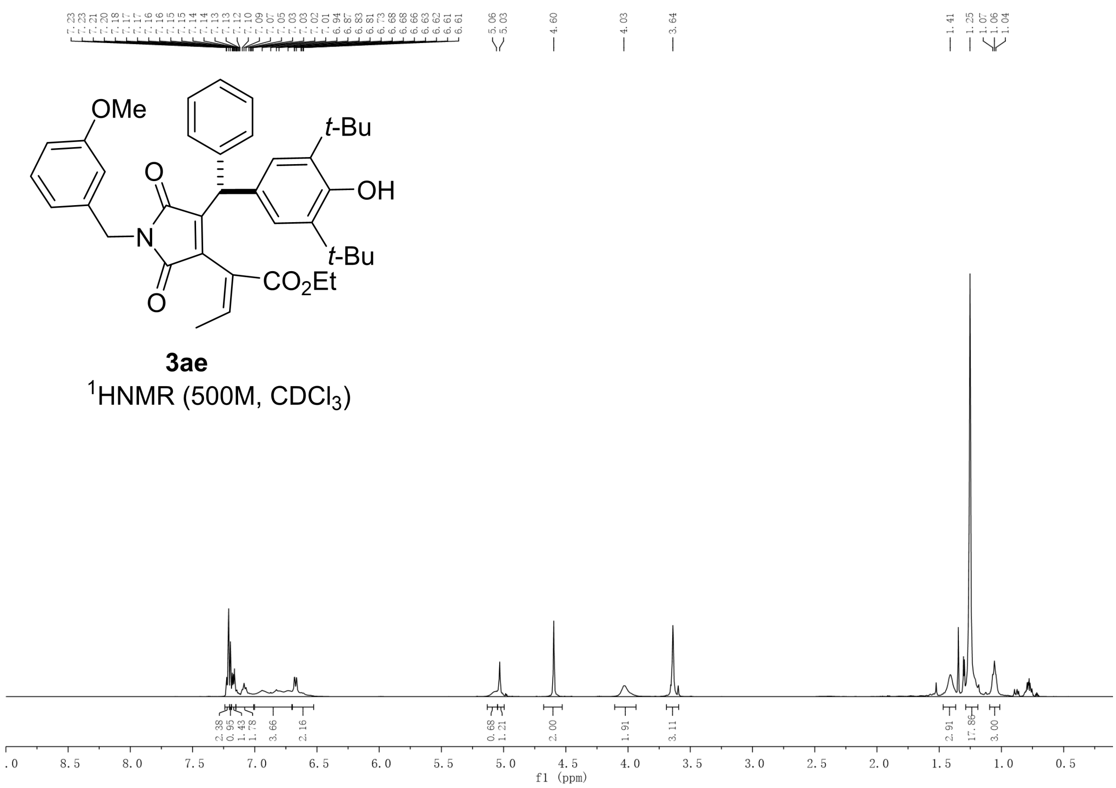

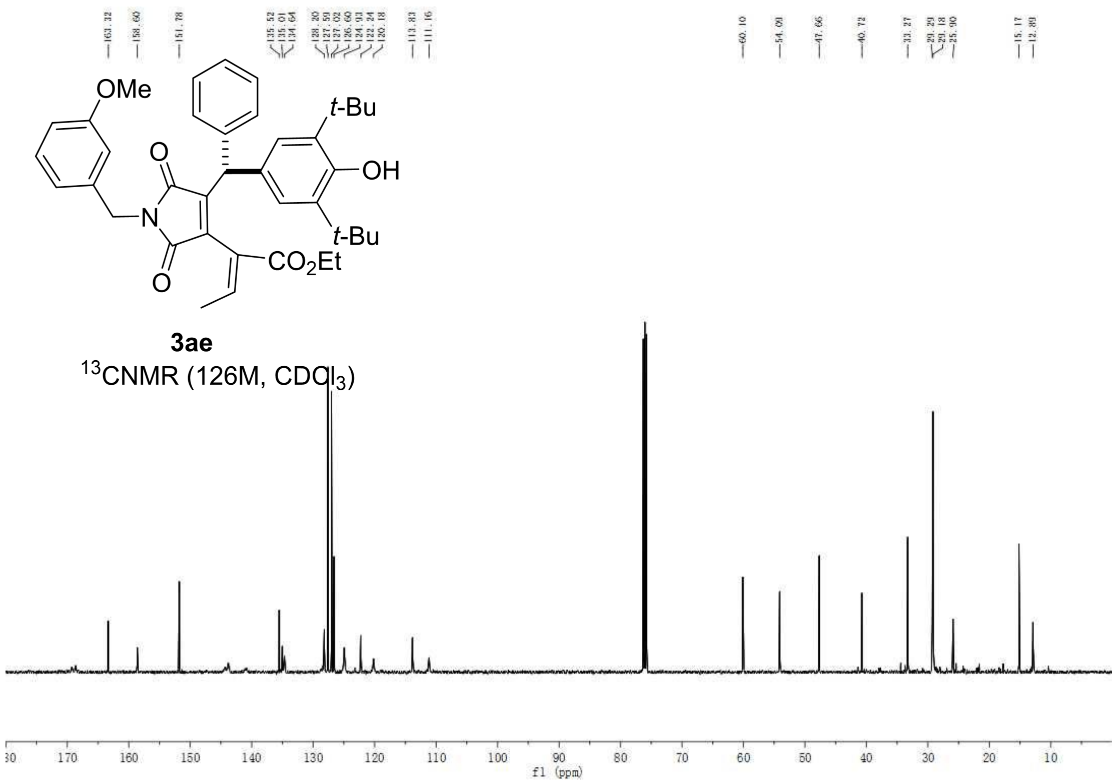

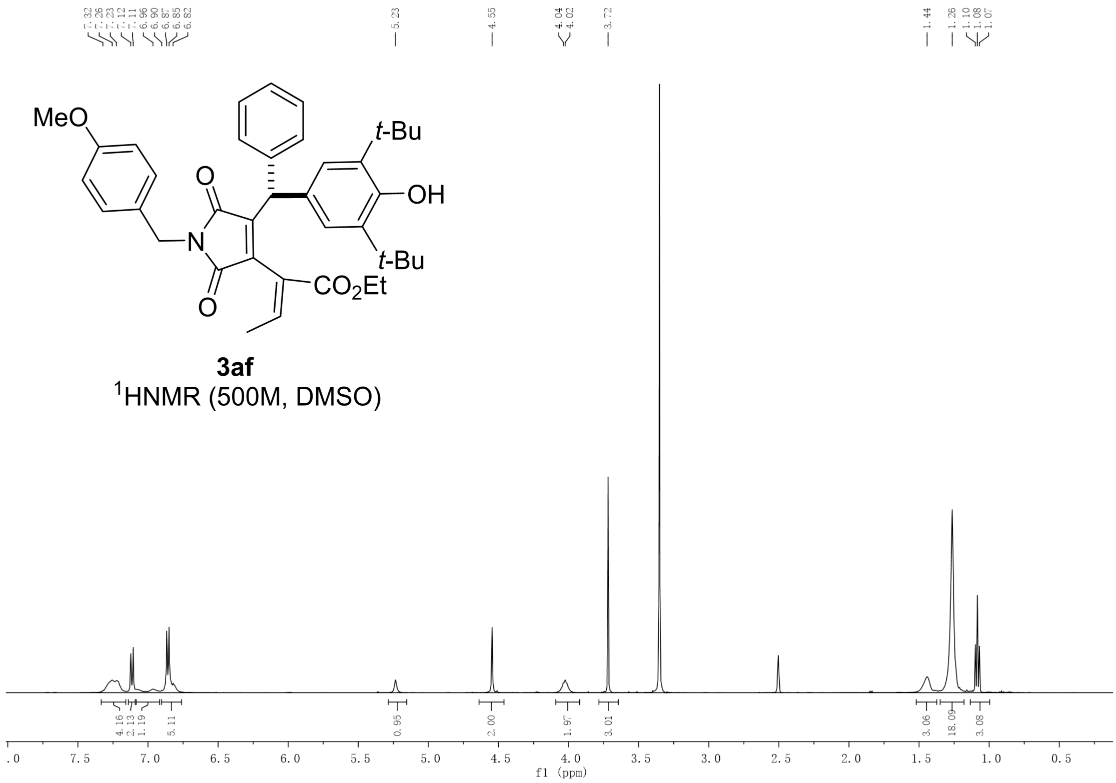
MeO
t-Bu
OH
t-Bu
CO2Et
N
O
O
3af
1HNMR (500M, DMSO)
f1 (ppm)

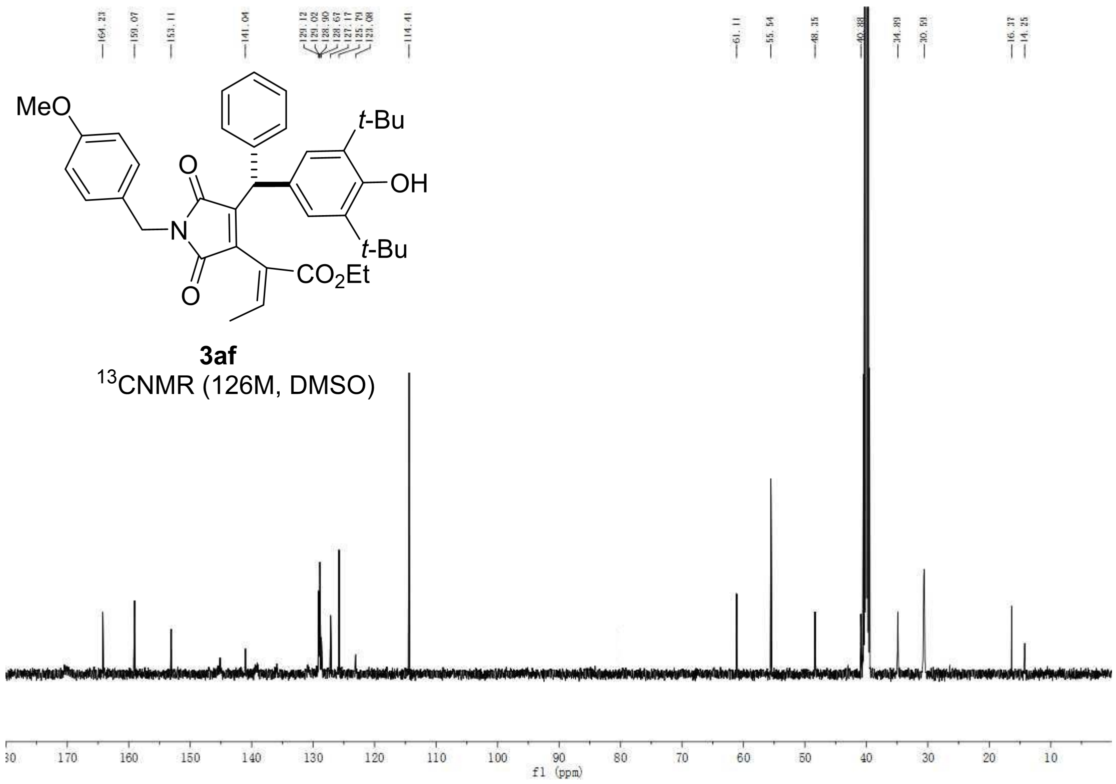
MeO
t-Bu
OH
t-Bu
CO2Et
N
O
O
3af
13CNMR (126M, DMSO)
f1 (ppm)

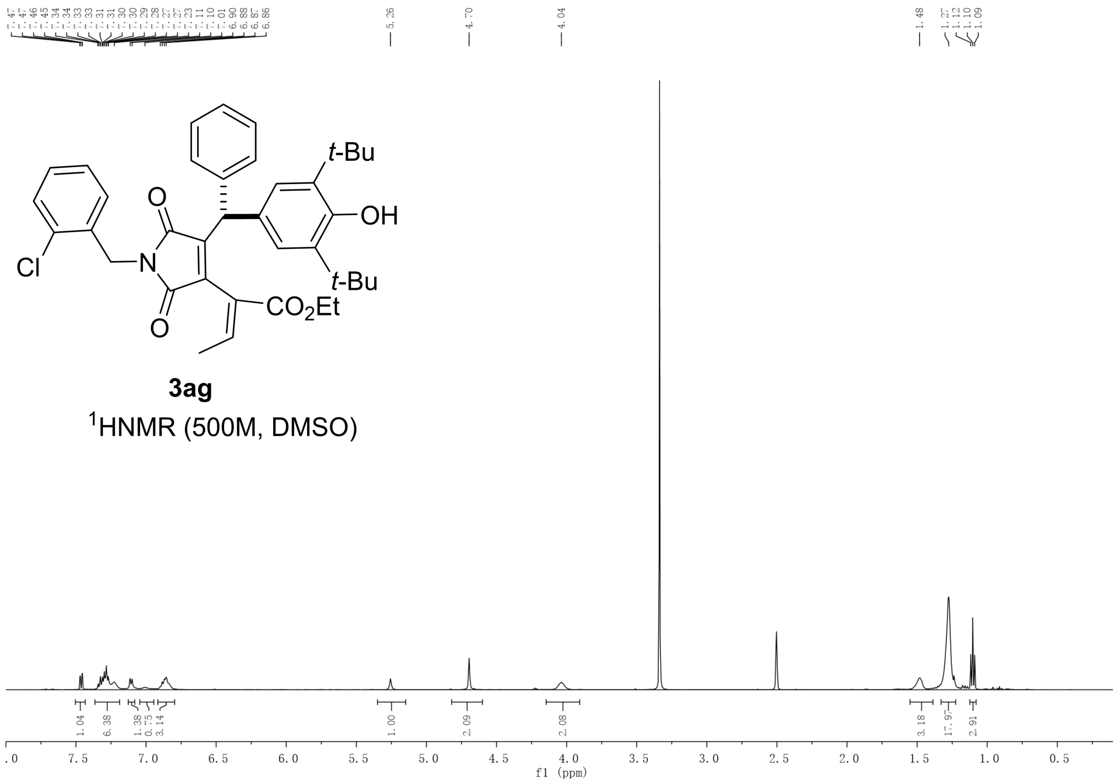
t-Bu
OH
t-Bu
CO2Et
Cl
N
O
O
3ag
1HNMR (500M, DMSO)
f1 (ppm)

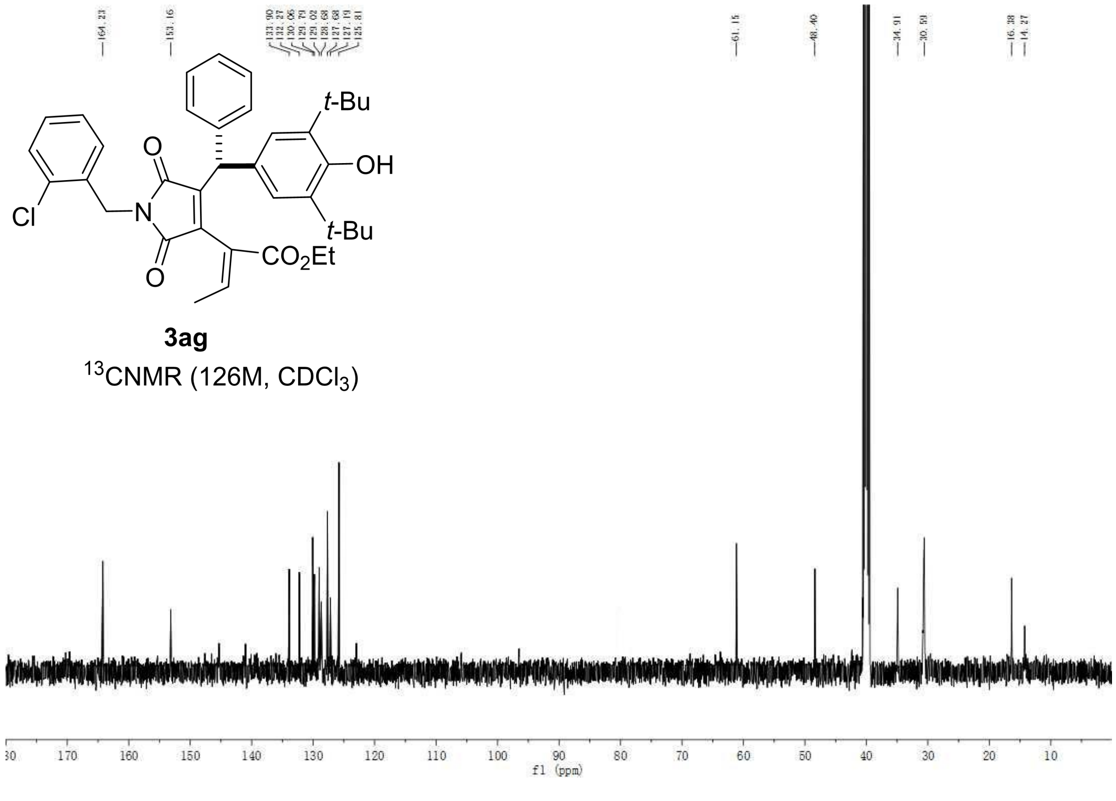
t-Bu
OH
t-Bu
CO2Et
Cl
N
O
O
3ag
13CNMR (126M, CDCl3)
f1 (ppm)

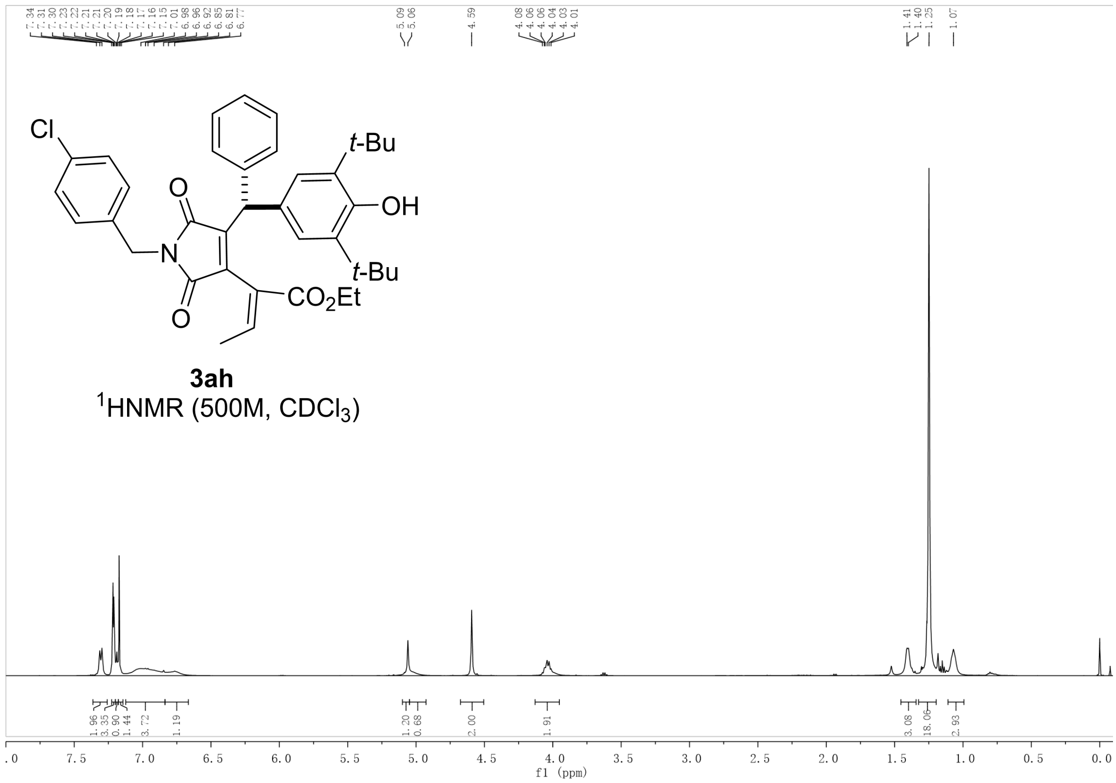
Cl
O
N
O
t-Bu
OH
t-Bu
CO2Et
3ah
1HNMR (500M, CDCl3)
f1 (ppm)

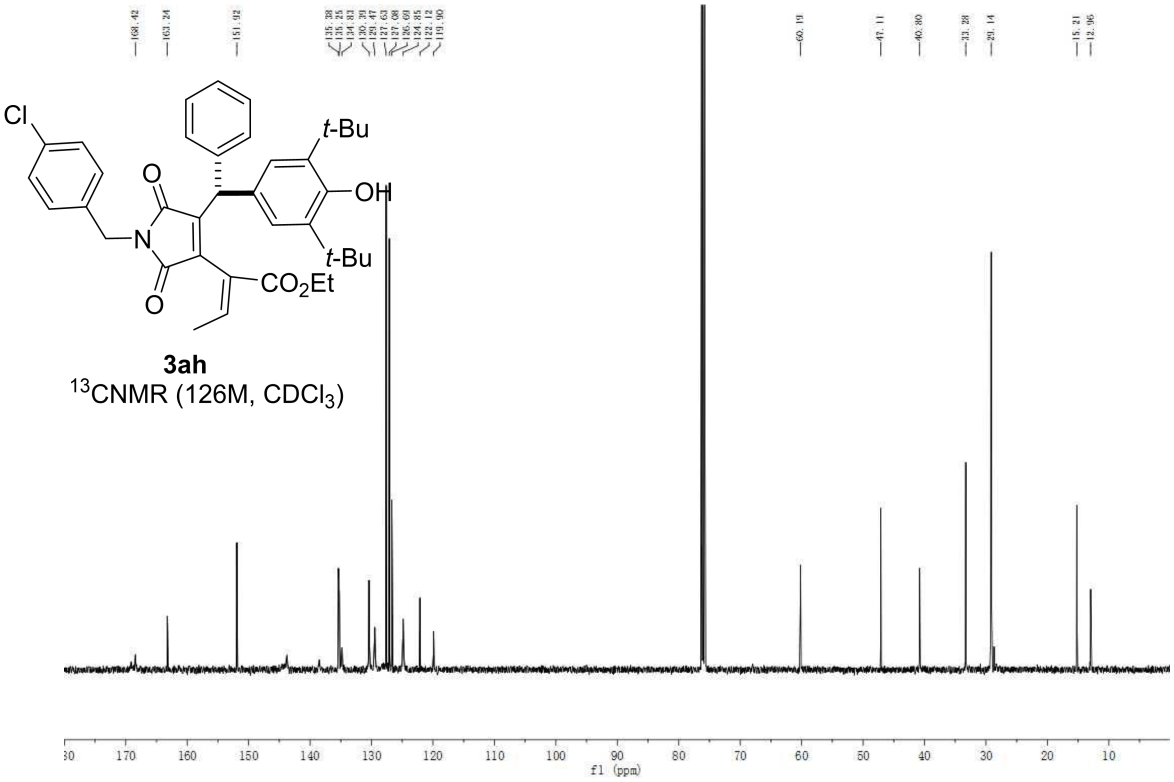
Cl
O
N
O
t-Bu
OH
t-Bu
CO2Et
3ah
13CNMR (126M, CDCl3)
f1 (ppm)

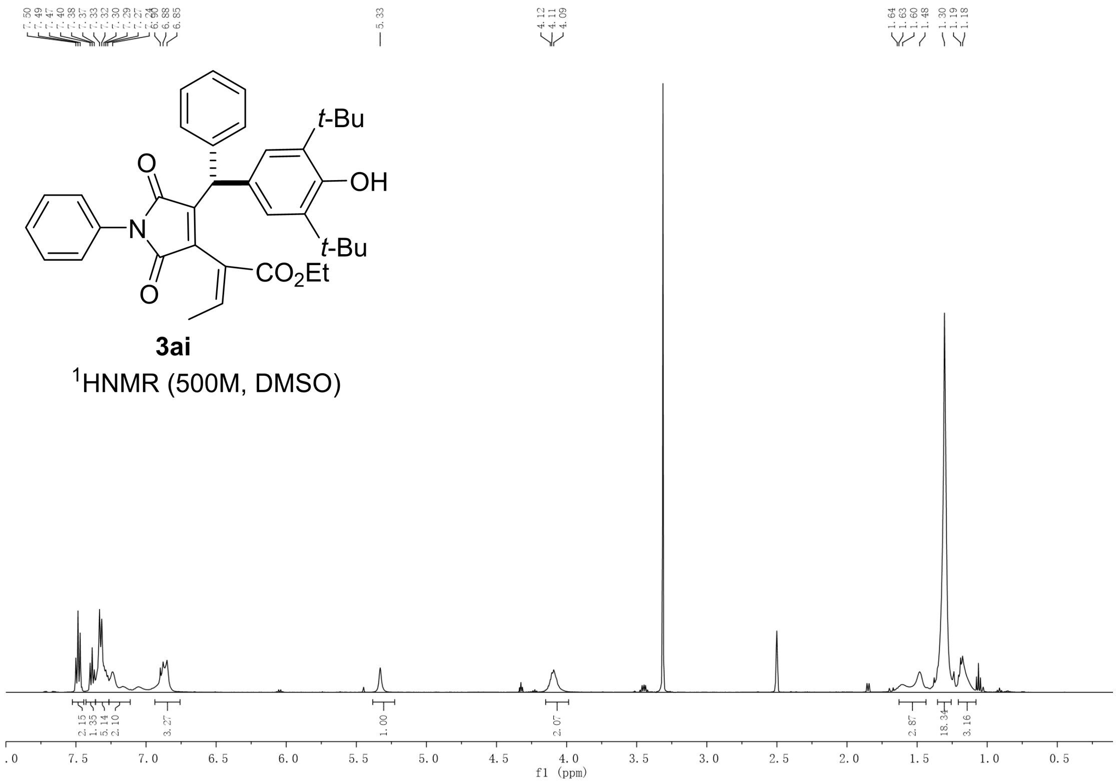
t-Bu
OH
t-Bu
CO_2Et
N
O
O
3ai
^{1}HNMR (500M, DMSO)
f1 (ppm)

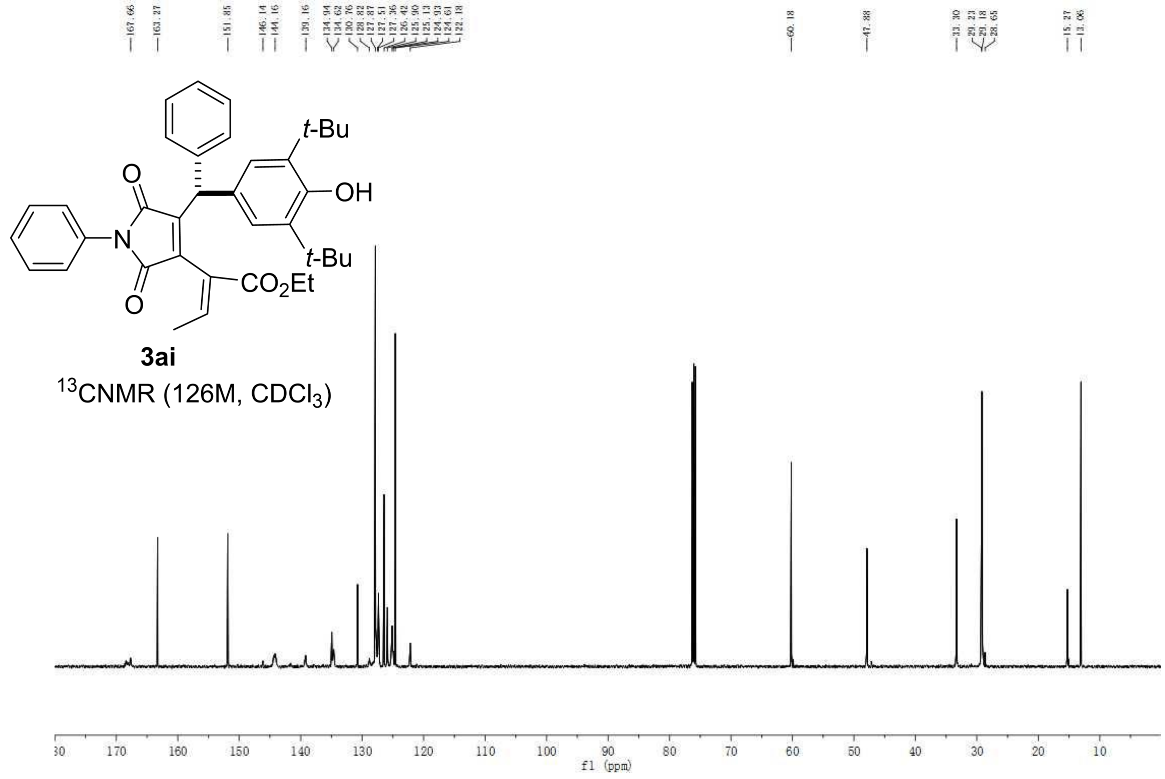
t-Bu
OH
t-Bu
CO_2Et
N
O
O
3ai
^{13}CNMR (126M, $CDCl_3$)
f1 (ppm)

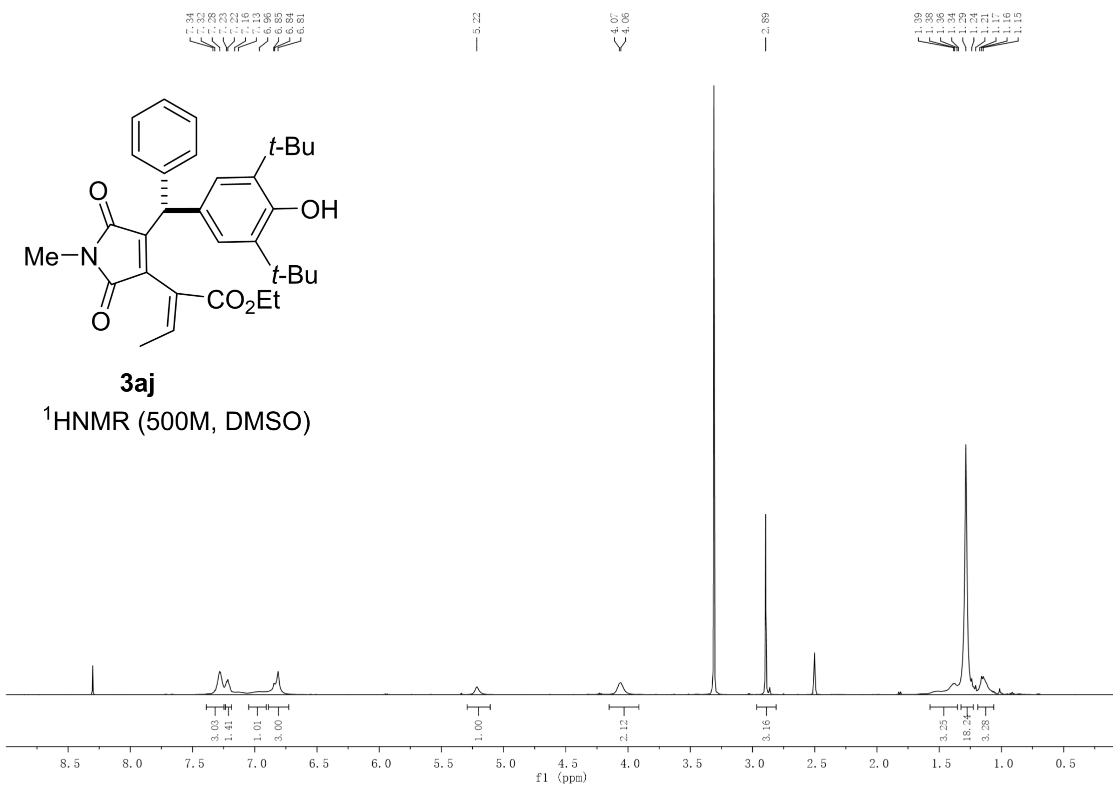

3aj

^{1}HNMR (500M, DMSO)

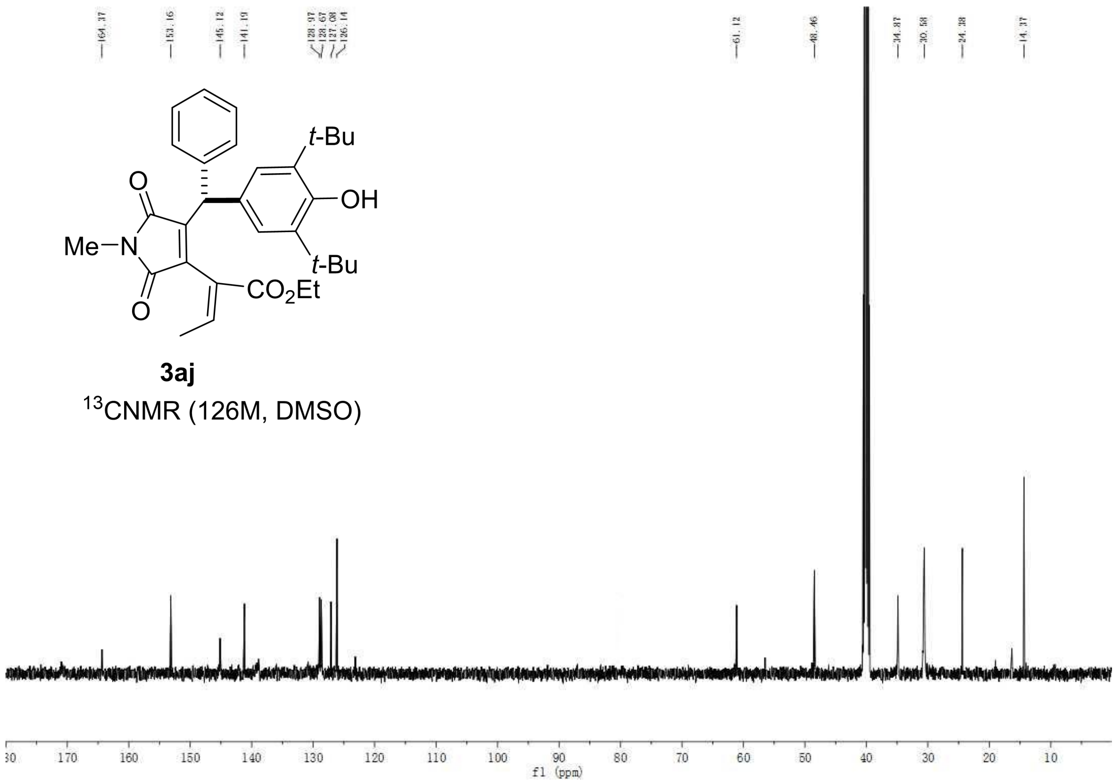

3aj

^{13}CNMR (126M, DMSO)

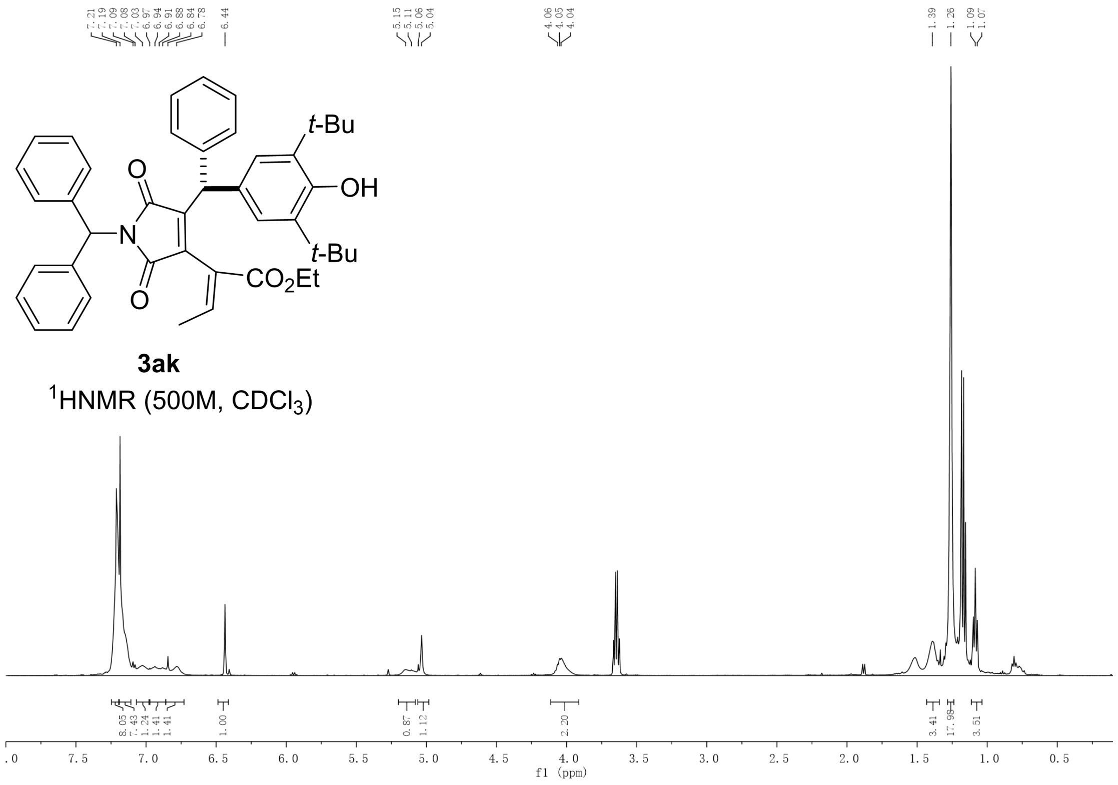
t-Bu
OH
t-Bu
CO2Et
N
O
O
3ak
1HNMR (500M, CDCl3)
f1 (ppm)

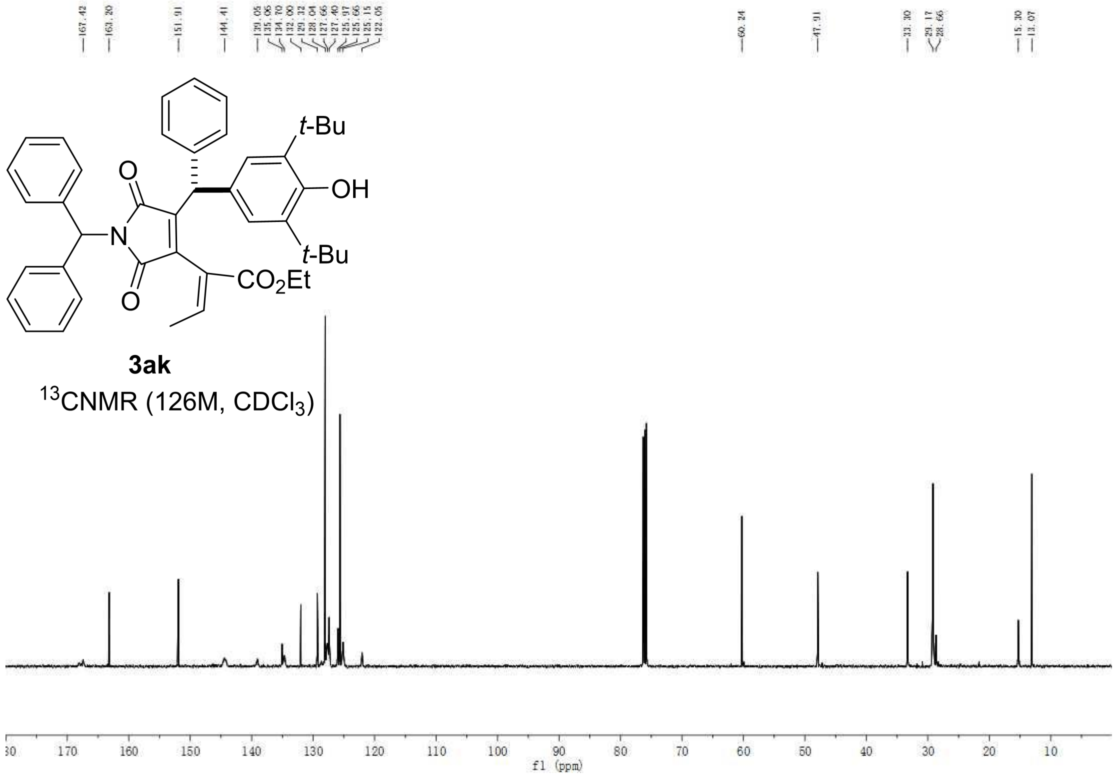
t-Bu
OH
t-Bu
CO2Et
N
O
O
3ak
13CNMR (126M, CDCl3)
f1 (ppm)

X-Ray Crystallographic Data of 3sa

Sample preparation: Single crystal suitable for X-ray diffraction was obtained by slow evaporation of a saturated solution of compound **3sa** (Hexane/ethyl acetate) in a loosely capped vial.

Crystal measurement: A crystal of **3sa** was picked up with paratone oil and mounted on a Bruker Smart Apexii diffractometer equipped with a Mo Kα (λ = 1.54184 Å) radiation source at 293 K.

Crystallographic data for **3sa** have been deposited with the Cam-bridge Crystallographic Data Centre as deposition number CCDC 2287621.

Thermal ellipsoids are drawn at 50% probability level.

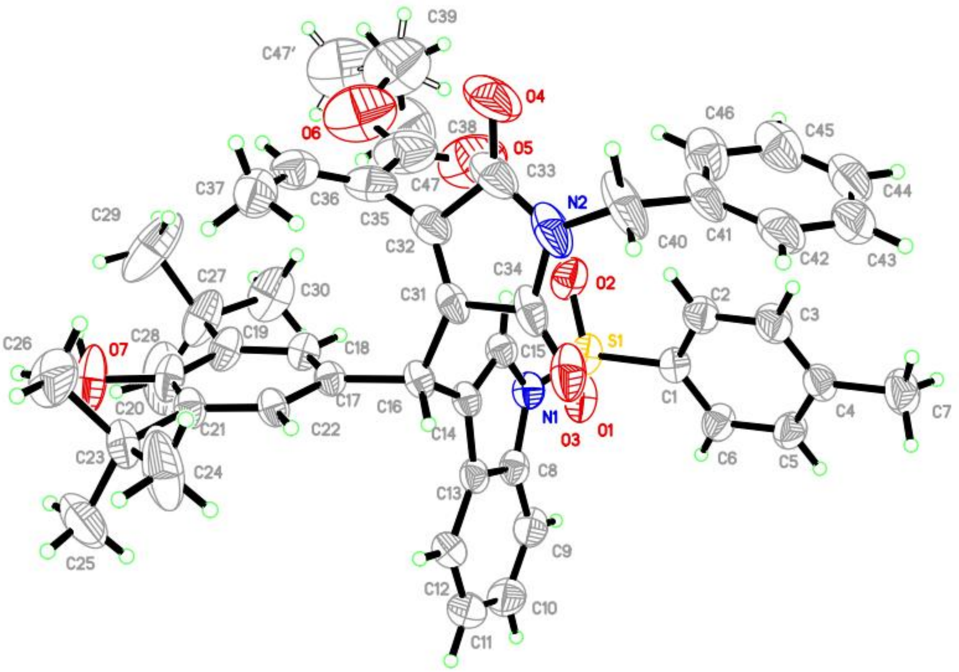

Table S1. Crystal data and structure refinement for 3sa.

Identification code	**3sa**
Empirical formula	$C_{47} H_{50} N_2 O_7 S$
Formula weight	786.95
Temperature/K	293
Crystal system	Triclinic
Space group	P-1
a/Å	9.6313(5)
b/Å	9.6819(5)
c/Å	23.3357(12)
α/°	85.571(4)

β/°	87.709(4)
γ/°	87.709(4)
Volume/Å^3	2161.74(19)
Z	2
ρ_{calc}g/cm^3	1.209
μ/mm^{-1}	1.083
F(000)	836
Crystal size/mm^3	0.120 x 0.120 x 0.110
Theta range for data collection/°	3.802 to 67.242
Index ranges	-11<=h<=10, -11<=k<=10, -26<=l<=27
Reflections collected	14541
Independent reflections	7719 [R(int) = 0.0229]
Data/restraints/parameters	7719 / 247 / 534
Goodness-of-fit on F^2	1.040
Final R indexes [I>=2σ (I)]	R_1 = 0.0834, wR_2 = 0.2366
Final R indexes [all data]	R_1 = 0.1071, wR_2 = 0.2637
Largest diff. peak/hole / e Å^{-3}	1.060 and -0.535

Table S2. Fractional Atomic Coordinates (×10^4) and Equivalent Isotropic Displacement Parameters (Å^2×10^3) for 3sa. U_{eq} is defined as 1/3 of of the trace of the orthogonalised U_{IJ} tensor.

Atom	x	y	z	U(eq)
S(1)	3273(1)	4692(1)	3103(1)	67(1)
N(1)	4574(3)	5679(3)	2912(1)	61(1)
N(2)	9860(4)	2943(5)	3521(2)	106(1)
O(1)	2024(3)	5529(4)	2988(1)	87(1)
O(2)	3600(3)	3431(3)	2829(1)	87(1)
O(3)	9188(3)	5065(4)	3832(1)	94(1)
O(4)	10353(5)	1196(4)	2898(2)	146(2)
O(5)	7135(7)	2081(7)	2340(3)	198(3)
O(6)	7756(6)	1913(7)	1448(3)	193(2)
O(7)	9521(4)	7961(5)	336(1)	125(2)
C(1)	3396(3)	4341(4)	3845(2)	58(1)
C(2)	4360(4)	3320(4)	4052(2)	74(1)
C(3)	4485(5)	3054(5)	4631(2)	82(1)
C(4)	3663(4)	3783(4)	5021(2)	69(1)
C(5)	2694(5)	4790(4)	4805(2)	75(1)
C(6)	2558(4)	5089(4)	4222(2)	72(1)
C(7)	3823(6)	3474(5)	5656(2)	94(1)
C(8)	4615(4)	7108(4)	2986(1)	59(1)
C(9)	3556(5)	8110(5)	3105(2)	78(1)

C(10)	3940(6)	9448(5)	3130(2)	92(1)
C(11)	5303(6)	9774(5)	3047(2)	92(1)
C(12)	6349(5)	8778(4)	2931(2)	73(1)
C(13)	6007(4)	7412(3)	2903(1)	56(1)
C(14)	6819(3)	6140(3)	2795(1)	52(1)
C(15)	5939(3)	5126(4)	2812(2)	58(1)
C(16)	8367(3)	6037(4)	2645(1)	55(1)
C(17)	8662(3)	6539(3)	2023(1)	51(1)
C(18)	7804(3)	6292(4)	1591(1)	59(1)
C(19)	8073(4)	6717(5)	1018(2)	72(1)
C(20)	9261(4)	7469(5)	899(2)	72(1)
C(21)	10151(4)	7750(4)	1322(2)	60(1)
C(22)	9825(3)	7268(3)	1882(1)	55(1)
C(23)	11450(4)	8585(5)	1187(2)	78(1)
C(24)	12275(7)	8650(9)	1722(3)	150(3)
C(25)	10997(7)	10062(6)	965(3)	142(2)
C(26)	12431(5)	7899(7)	738(3)	126(2)
C(27)	7108(5)	6384(8)	547(2)	109(1)
C(28)	6470(6)	7721(9)	239(3)	155(2)
C(29)	7926(7)	5441(12)	127(3)	190(3)
C(30)	5892(6)	5574(8)	801(2)	119(2)
C(31)	8998(3)	4607(4)	2826(2)	63(1)
C(32)	9348(4)	3481(4)	2554(2)	78(1)
C(33)	9911(5)	2380(5)	3001(3)	101(1)
C(34)	9335(4)	4304(5)	3461(2)	77(1)
C(35)	9183(5)	3122(5)	1976(3)	94(1)
C(36)	10111(6)	3336(5)	1550(2)	93(1)
C(37)	11443(6)	3958(6)	1590(2)	106(2)
C(38)	7891(8)	2340(8)	1929(5)	148(2)
C(39)	6738(11)	817(11)	1431(5)	199(3)
C(40)	10378(5)	2220(7)	4047(3)	128(2)
C(41)	9233(5)	1620(5)	4431(2)	89(1)
C(42)	9218(6)	1772(5)	5005(3)	101(1)
C(43)	8238(7)	1186(6)	5371(3)	113(2)
C(44)	7299(6)	424(6)	5171(3)	112(2)
C(45)	7273(6)	246(6)	4608(3)	114(2)
C(46)	8241(5)	841(6)	4227(3)	105(1)
C(47)	5491(16)	1808(18)	1431(10)	220(6)
C(47')	6090(30)	1090(30)	859(9)	210(8)

Table S3. Anisotropic displacement parameters (A^2 x 10^3) for 3sa. The anisotropic displacement factor exponent takes the form: -2 pi^2 [h^2 a*^2 U11+ ... + 2 h k a* b* U12]

Atom	U11	U22	U33	U23	U13	U12
S(1)	51(1)	98(1)	55(1)	-4(1)	-7(1)	-18(1)
N(1)	50(1)	73(1)	59(1)	2(1)	-2(1)	-4(1)
N(2)	84(2)	104(2)	116(2)	61(1)	10(2)	19(2)
O(1)	51(1)	143(2)	64(2)	13(1)	-13(1)	-10(1)
O(2)	88(2)	109(1)	72(1)	-26(1)	2(1)	-40(1)
O(3)	73(2)	135(2)	64(1)	31(1)	2(1)	13(2)
O(4)	133(3)	80(2)	207(4)	40(2)	29(3)	36(2)
O(5)	154(4)	164(5)	293(6)	-91(5)	60(4)	-79(4)
O(6)	164(4)	192(5)	241(5)	-89(4)	-48(4)	-43(3)
O(7)	87(2)	231(5)	50(1)	32(2)	2(1)	-18(2)
C(1)	51(2)	68(2)	56(1)	-1(1)	-6(1)	-10(1)
C(2)	72(2)	75(2)	72(1)	3(2)	0(2)	7(2)
C(3)	85(3)	79(3)	77(1)	13(2)	-10(2)	16(2)
C(4)	78(2)	65(2)	62(1)	7(1)	-12(1)	-10(1)
C(5)	86(3)	79(2)	57(1)	-5(2)	-7(2)	9(2)
C(6)	69(2)	85(2)	58(1)	-2(2)	-8(1)	11(2)
C(7)	116(4)	101(3)	66(1)	14(2)	-22(2)	-11(3)
C(8)	58(1)	69(1)	47(2)	7(1)	-5(1)	6(1)
C(9)	72(2)	88(2)	66(2)	4(2)	2(2)	21(1)
C(10)	105(2)	79(1)	85(3)	-1(2)	9(3)	29(2)
C(11)	120(2)	62(2)	89(3)	0(2)	11(3)	8(1)
C(12)	88(2)	59(1)	68(2)	4(2)	3(2)	-6(1)
C(13)	62(1)	59(1)	44(2)	7(1)	-5(1)	-1(1)
C(14)	51(1)	57(1)	47(2)	10(1)	-5(1)	-3(1)
C(15)	51(1)	61(1)	59(2)	2(2)	1(1)	-5(1)
C(16)	51(1)	59(1)	54(1)	12(1)	-6(1)	-7(1)
C(17)	47(1)	54(2)	50(1)	5(1)	-5(1)	-1(1)
C(18)	49(2)	74(2)	54(1)	-4(1)	-4(1)	-5(1)
C(19)	55(2)	110(3)	50(1)	-8(1)	-5(1)	1(1)
C(20)	56(2)	112(3)	46(1)	10(2)	2(1)	4(1)
C(21)	53(1)	69(2)	55(1)	14(1)	-1(1)	2(1)
C(22)	50(2)	61(2)	51(1)	10(1)	-6(1)	-6(1)
C(23)	69(2)	91(2)	71(2)	20(2)	9(1)	-14(2)
C(24)	130(4)	235(7)	96(3)	35(3)	-18(2)	-122(5)
C(25)	136(5)	87(2)	193(6)	47(3)	40(4)	-15(2)
C(26)	65(3)	160(4)	154(4)	-27(4)	39(3)	-16(3)
C(27)	68(2)	203(4)	60(2)	-31(2)	-11(1)	-7(2)
C(28)	87(4)	281(6)	90(4)	51(4)	-39(3)	-20(3)
C(29)	111(4)	356(9)	122(5)	-143(6)	-3(3)	-13(5)
C(30)	83(3)	191(5)	93(3)	-39(3)	-26(2)	-30(3)
C(31)	45(1)	64(1)	74(1)	23(1)	4(1)	-4(1)
C(32)	60(2)	64(1)	105(1)	17(1)	9(2)	1(1)

C(33)	74(3)	72(2)	147(2)	39(1)	7(3)	10(2)
C(34)	47(2)	96(2)	78(1)	41(1)	4(2)	5(2)
C(35)	80(2)	66(2)	135(2)	-19(2)	-13(2)	12(2)
C(36)	112(3)	79(3)	90(2)	-16(2)	-25(2)	6(2)
C(37)	124(3)	108(4)	87(3)	-13(3)	12(2)	-22(3)
C(38)	129(4)	108(4)	218(5)	-65(4)	2(3)	-34(3)
C(39)	187(6)	212(7)	219(8)	-86(6)	-39(6)	-66(5)
C(40)	77(3)	138(4)	149(2)	97(3)	-5(2)	5(2)
C(41)	72(2)	77(3)	107(2)	50(2)	-4(2)	14(2)
C(42)	108(3)	71(3)	116(2)	24(2)	-9(2)	15(2)
C(43)	138(4)	85(4)	103(2)	19(2)	20(2)	27(2)
C(44)	101(3)	90(4)	132(3)	43(3)	32(3)	21(2)
C(45)	90(3)	104(4)	141(3)	29(3)	5(3)	-9(2)
C(46)	83(3)	121(4)	105(2)	25(3)	-1(2)	1(2)
C(47)	162(4)	241(12)	261(15)	11(13)	14(11)	-71(6)
C(47')	180(15)	220(16)	250(12)	-70(12)	-66(11)	-61(13)

Table S4. Bond Lengths for 3sa.

Atom-Atom	Length/Å
S(1)-O(1)	1.421(3)
S(1)-O(2)	1.429(3)
S(1)-N(1)	1.661(3)
S(1)-C(1)	1.745(4)
N(1)-C(15)	1.398(4)
N(1)-C(8)	1.411(5)
N(2)-C(33)	1.366(8)
N(2)-C(34)	1.373(6)
N(2)-C(40)	1.453(6)
O(3)-C(34)	1.178(6)
O(4)-C(33)	1.229(6)
O(5)-C(38)	1.202(10)
O(6)-C(38)	1.241(10)
O(6)-C(39)	1.504(7)
O(7)-C(20)	1.380(4)
O(7)-H(7E)	0.8500
C(1)-C(2)	1.376(5)
C(1)-C(6)	1.379(5)
C(2)-C(3)	1.364(6)
C(2)-H(2)	0.9300
C(3)-C(4)	1.382(6)
C(3)-H(3)	0.9300
C(4)-C(5)	1.377(5)

C(4)-C(7)	1.500(5)
C(5)-C(6)	1.379(5)
C(5)-H(5)	0.9300
C(6)-H(6)	0.9300
C(7)-H(7A)	0.9600
C(7)-H(7B)	0.9600
C(7)-H(7C)	0.9600
C(8)-C(9)	1.387(5)
C(8)-C(13)	1.397(5)
C(9)-C(10)	1.381(7)
C(9)-H(9)	0.9300
C(10)-C(11)	1.376(7)
C(10)-H(10)	0.9300
C(11)-C(12)	1.374(6)
C(11)-H(11)	0.9300
C(12)-C(13)	1.394(5)
C(12)-H(12)	0.9300
C(13)-C(14)	1.442(5)
C(14)-C(15)	1.343(5)
C(14)-C(16)	1.514(4)
C(15)-H(15)	0.9300
C(16)-C(31)	1.505(5)
C(16)-C(17)	1.519(4)
C(16)-H(16)	0.9800
C(17)-C(18)	1.374(5)
C(17)-C(22)	1.385(4)
C(18)-C(19)	1.389(5)
C(18)-H(18)	0.9300
C(19)-C(20)	1.411(6)
C(19)-C(27)	1.537(6)
C(20)-C(21)	1.389(5)
C(21)-C(22)	1.386(4)
C(21)-C(23)	1.551(5)
C(22)-H(22)	0.9300
C(23)-C(24)	1.513(7)
C(23)-C(25)	1.520(7)
C(23)-C(26)	1.537(7)
C(24)-H(24A)	0.9600
C(24)-H(24B)	0.9600
C(24)-H(24C)	0.9600
C(25)-H(25A)	0.9600
C(25)-H(25B)	0.9600
C(25)-H(25C)	0.9600

C(26)-H(26A)	0.9600
C(26)-H(26B)	0.9600
C(26)-H(26C)	0.9600
C(27)-C(28)	1.528(9)
C(27)-C(30)	1.534(8)
C(27)-C(29)	1.542(8)
C(28)-H(28A)	0.9600
C(28)-H(28B)	0.9600
C(28)-H(28C)	0.9600
C(29)-H(29A)	0.9600
C(29)-H(29B)	0.9600
C(29)-H(29C)	0.9600
C(30)-H(30A)	0.9600
C(30)-H(30B)	0.9600
C(30)-H(30C)	0.9600
C(31)-C(32)	1.318(6)
C(31)-C(34)	1.531(6)
C(32)-C(35)	1.435(7)
C(32)-C(33)	1.517(6)
C(35)-C(36)	1.327(7)
C(35)-C(38)	1.518(7)
C(36)-C(37)	1.467(8)
C(36)-H(36)	0.9300
C(37)-H(37A)	0.9600
C(37)-H(37B)	0.9600
C(37)-H(37C)	0.9600
C(39)-C(47)	1.478(9)
C(39)-C(47')	1.489(10)
C(39)-H(39A)	0.9701
C(39)-H(39B)	0.9699
C(39)-H(39C)	0.9700
C(39)-H(39D)	0.9700
C(40)-C(41)	1.515(6)
C(40)-H(40A)	0.9700
C(40)-H(40B)	0.9700
C(41)-C(42)	1.357(7)
C(41)-C(46)	1.382(8)
C(42)-C(43)	1.374(8)
C(42)-H(42)	0.9300
C(43)-C(44)	1.330(9)
C(43)-H(43)	0.9300
C(44)-C(45)	1.339(9)
C(44)-H(44)	0.9300

C(45)-C(46)	1.389(7)
C(45)-H(45)	0.9300
C(46)-H(46)	0.9300
C(47)-H(47A)	0.9600
C(47)-H(47B)	0.9600
C(47)-H(47C)	0.9600
C(47')-H(47D)	0.9600
C(47')-H(47E)	0.9600
C(47')-H(47F)	0.9600

Table S5. Bond Angles for 3sa.

Atom-Atom-Atom	Angle/°
O(1)-S(1)-O(2)	121.15(19)
O(1)-S(1)-N(1)	106.31(17)
O(2)-S(1)-N(1)	105.31(17)
O(1)-S(1)-C(1)	109.13(17)
O(2)-S(1)-C(1)	108.75(19)
N(1)-S(1)-C(1)	104.98(16)
C(15)-N(1)-C(8)	108.1(3)
C(15)-N(1)-S(1)	122.6(3)
C(8)-N(1)-S(1)	126.8(2)
C(33)-N(2)-C(34)	110.0(4)
C(33)-N(2)-C(40)	124.2(5)
C(34)-N(2)-C(40)	125.6(6)
C(38)-O(6)-C(39)	115.1(8)
C(20)-O(7)-H(7E)	108.5
C(2)-C(1)-C(6)	120.0(3)
C(2)-C(1)-S(1)	119.1(3)
C(6)-C(1)-S(1)	120.8(3)
C(3)-C(2)-C(1)	119.7(4)
C(3)-C(2)-H(2)	120.1
C(1)-C(2)-H(2)	120.1
C(2)-C(3)-C(4)	121.8(4)
C(2)-C(3)-H(3)	119.1
C(4)-C(3)-H(3)	119.1
C(5)-C(4)-C(3)	117.6(4)
C(5)-C(4)-C(7)	121.6(4)
C(3)-C(4)-C(7)	120.8(4)
C(4)-C(5)-C(6)	121.7(4)
C(4)-C(5)-H(5)	119.1
C(6)-C(5)-H(5)	119.1
C(5)-C(6)-C(1)	119.1(4)

C(5)-C(6)-H(6)	120.4
C(1)-C(6)-H(6)	120.4
C(4)-C(7)-H(7A)	109.5
C(4)-C(7)-H(7B)	109.5
H(7A)-C(7)-H(7B)	109.5
C(4)-C(7)-H(7C)	109.5
H(7A)-C(7)-H(7C)	109.5
H(7B)-C(7)-H(7C)	109.5
C(9)-C(8)-C(13)	122.5(4)
C(9)-C(8)-N(1)	130.8(4)
C(13)-C(8)-N(1)	106.6(3)
C(10)-C(9)-C(8)	116.5(4)
C(10)-C(9)-H(9)	121.7
C(8)-C(9)-H(9)	121.7
C(11)-C(10)-C(9)	122.0(4)
C(11)-C(10)-H(10)	119.0
C(9)-C(10)-H(10)	119.0
C(12)-C(11)-C(10)	121.3(4)
C(12)-C(11)-H(11)	119.3
C(10)-C(11)-H(11)	119.3
C(11)-C(12)-C(13)	118.5(4)
C(11)-C(12)-H(12)	120.7
C(13)-C(12)-H(12)	120.7
C(12)-C(13)-C(8)	119.1(3)
C(12)-C(13)-C(14)	133.0(3)
C(8)-C(13)-C(14)	107.9(3)
C(15)-C(14)-C(13)	107.5(3)
C(15)-C(14)-C(16)	127.8(3)
C(13)-C(14)-C(16)	124.6(3)
C(14)-C(15)-N(1)	109.8(3)
C(14)-C(15)-H(15)	125.1
N(1)-C(15)-H(15)	125.1
C(31)-C(16)-C(14)	110.1(3)
C(31)-C(16)-C(17)	115.4(3)
C(14)-C(16)-C(17)	111.5(3)
C(31)-C(16)-H(16)	106.4
C(14)-C(16)-H(16)	106.4
C(17)-C(16)-H(16)	106.4
C(18)-C(17)-C(22)	118.7(3)
C(18)-C(17)-C(16)	121.8(3)
C(22)-C(17)-C(16)	119.5(3)
C(17)-C(18)-C(19)	122.6(3)
C(17)-C(18)-H(18)	118.7

C(19)-C(18)-H(18)	118.7
C(18)-C(19)-C(20)	116.5(3)
C(18)-C(19)-C(27)	120.9(4)
C(20)-C(19)-C(27)	122.6(4)
O(7)-C(20)-C(21)	119.4(4)
O(7)-C(20)-C(19)	117.8(4)
C(21)-C(20)-C(19)	122.8(3)
C(22)-C(21)-C(20)	117.3(3)
C(22)-C(21)-C(23)	120.2(3)
C(20)-C(21)-C(23)	122.5(3)
C(17)-C(22)-C(21)	122.2(3)
C(17)-C(22)-H(22)	118.9
C(21)-C(22)-H(22)	118.9
C(24)-C(23)-C(25)	108.3(6)
C(24)-C(23)-C(26)	107.2(5)
C(25)-C(23)-C(26)	109.5(5)
C(24)-C(23)-C(21)	111.0(3)
C(25)-C(23)-C(21)	109.7(4)
C(26)-C(23)-C(21)	111.0(4)
C(23)-C(24)-H(24A)	109.5
C(23)-C(24)-H(24B)	109.5
H(24A)-C(24)-H(24B)	109.5
C(23)-C(24)-H(24C)	109.5
H(24A)-C(24)-H(24C)	109.5
H(24B)-C(24)-H(24C)	109.5
C(23)-C(25)-H(25A)	109.5
C(23)-C(25)-H(25B)	109.5
H(25A)-C(25)-H(25B)	109.5
C(23)-C(25)-H(25C)	109.5
H(25A)-C(25)-H(25C)	109.5
H(25B)-C(25)-H(25C)	109.5
C(23)-C(26)-H(26A)	109.5
C(23)-C(26)-H(26B)	109.5
H(26A)-C(26)-H(26B)	109.5
C(23)-C(26)-H(26C)	109.5
H(26A)-C(26)-H(26C)	109.5
H(26B)-C(26)-H(26C)	109.5
C(28)-C(27)-C(30)	106.9(5)
C(28)-C(27)-C(19)	110.6(5)
C(30)-C(27)-C(19)	111.3(4)
C(28)-C(27)-C(29)	112.1(6)
C(30)-C(27)-C(29)	106.4(6)
C(19)-C(27)-C(29)	109.4(4)

C(27)-C(28)-H(28A)	109.5
C(27)-C(28)-H(28B)	109.5
H(28A)-C(28)-H(28B)	109.5
C(27)-C(28)-H(28C)	109.5
H(28A)-C(28)-H(28C)	109.5
H(28B)-C(28)-H(28C)	109.5
C(27)-C(29)-H(29A)	109.5
C(27)-C(29)-H(29B)	109.5
H(29A)-C(29)-H(29B)	109.5
C(27)-C(29)-H(29C)	109.5
H(29A)-C(29)-H(29C)	109.5
H(29B)-C(29)-H(29C)	109.5
C(27)-C(30)-H(30A)	109.5
C(27)-C(30)-H(30B)	109.5
H(30A)-C(30)-H(30B)	109.5
C(27)-C(30)-H(30C)	109.5
H(30A)-C(30)-H(30C)	109.5
H(30B)-C(30)-H(30C)	109.5
C(32)-C(31)-C(16)	134.1(4)
C(32)-C(31)-C(34)	108.7(4)
C(16)-C(31)-C(34)	117.2(4)
C(31)-C(32)-C(35)	133.5(4)
C(31)-C(32)-C(33)	106.7(5)
C(35)-C(32)-C(33)	119.6(4)
O(4)-C(33)-N(2)	127.1(5)
O(4)-C(33)-C(32)	124.6(6)
N(2)-C(33)-C(32)	108.3(4)
O(3)-C(34)-N(2)	125.7(4)
O(3)-C(34)-C(31)	128.0(4)
N(2)-C(34)-C(31)	106.3(4)
C(36)-C(35)-C(32)	124.0(5)
C(36)-C(35)-C(38)	124.2(6)
C(32)-C(35)-C(38)	111.6(6)
C(35)-C(36)-C(37)	126.1(5)
C(35)-C(36)-H(36)	116.9
C(37)-C(36)-H(36)	116.9
C(36)-C(37)-H(37A)	109.5
C(36)-C(37)-H(37B)	109.5
H(37A)-C(37)-H(37B)	109.5
C(36)-C(37)-H(37C)	109.5
H(37A)-C(37)-H(37C)	109.5
H(37B)-C(37)-H(37C)	109.5
O(5)-C(38)-O(6)	123.9(7)

O(5)-C(38)-C(35)	121.7(8)
O(6)-C(38)-C(35)	114.2(8)
C(47)-C(39)-O(6)	94.7(10)
C(47')-C(39)-O(6)	104.8(12)
C(47)-C(39)-H(39A)	111.7
O(6)-C(39)-H(39A)	113.3
C(47)-C(39)-H(39B)	113.6
O(6)-C(39)-H(39B)	112.8
H(39A)-C(39)-H(39B)	110.0
C(47')-C(39)-H(39C)	107.8
O(6)-C(39)-H(39C)	112.8
C(47')-C(39)-H(39D)	113.1
O(6)-C(39)-H(39D)	109.8
H(39C)-C(39)-H(39D)	108.6
N(2)-C(40)-C(41)	112.9(4)
N(2)-C(40)-H(40A)	109.0
C(41)-C(40)-H(40A)	109.0
N(2)-C(40)-H(40B)	109.0
C(41)-C(40)-H(40B)	109.0
H(40A)-C(40)-H(40B)	107.8
C(42)-C(41)-C(46)	117.9(5)
C(42)-C(41)-C(40)	119.3(6)
C(46)-C(41)-C(40)	122.6(6)
C(41)-C(42)-C(43)	121.1(6)
C(41)-C(42)-H(42)	119.4
C(43)-C(42)-H(42)	119.4
C(44)-C(43)-C(42)	120.4(6)
C(44)-C(43)-H(43)	119.8
C(42)-C(43)-H(43)	119.8
C(43)-C(44)-C(45)	120.6(6)
C(43)-C(44)-H(44)	119.7
C(45)-C(44)-H(44)	119.7
C(44)-C(45)-C(46)	120.2(7)
C(44)-C(45)-H(45)	119.9
C(46)-C(45)-H(45)	119.9
C(41)-C(46)-C(45)	119.8(6)
C(41)-C(46)-H(46)	120.1
C(45)-C(46)-H(46)	120.1
C(39)-C(47)-H(47A)	109.5
C(39)-C(47)-H(47B)	109.5
H(47A)-C(47)-H(47B)	109.5
C(39)-C(47)-H(47C)	109.5
H(47A)-C(47)-H(47C)	109.5

H(47B)-C(47)-H(47C)	109.5
C(39)-C(47')-H(47D)	109.5
C(39)-C(47')-H(47E)	109.5
H(47D)-C(47')-H(47E)	109.5
C(39)-C(47')-H(47F)	109.5
H(47D)-C(47')-H(47F)	109.5
H(47E)-C(47')-H(47F)	109.5

Table S6. Hydrogen Atom Coordinates (Å×10^4) and Isotropic Displacement Parameters (Å^2×10^3) for 3sa.

Atom	x	y	z	U(eq)
H(7E)	9631	7272	130	187
H(2)	4923	2812	3798	89
H(3)	5141	2364	4767	99
H(5)	2116	5282	5059	90
H(6)	1909	5785	4085	86
H(7A)	3907	4324	5833	141
H(7B)	4644	2864	5722	141
H(7C)	3021	3037	5818	141
H(9)	2635	7892	3164	93
H(10)	3257	10150	3206	111
H(11)	5519	10687	3070	110
H(12)	7266	9009	2872	87
H(15)	6199	4195	2765	69
H(16)	8784	6679	2881	66
H(18)	7011	5822	1687	71
H(22)	10407	7439	2173	66
H(24A)	13028	9235	1636	225
H(24B)	12643	7732	1848	225
H(24C)	11677	9024	2020	225
H(25A)	10281	10442	1218	214
H(25B)	10644	10055	586	214
H(25C)	11781	10620	950	214
H(26A)	13320	8285	733	190
H(26B)	12038	8066	366	190
H(26C)	12548	6917	837	190
H(28A)	6015	8300	517	232
H(28B)	5802	7496	-27	232
H(28C)	7192	8206	35	232
H(29A)	8403	6004	-162	285

H(29B)	7292	4904	-52	285
H(29C)	8594	4828	335	285
H(30A)	6250	4710	989	179
H(30B)	5302	5391	498	179
H(30C)	5364	6112	1074	179
H(36)	9898	3067	1191	112
H(37A)	11538	4213	1975	159
H(37B)	11466	4770	1327	159
H(37C)	12195	3298	1493	159
H(39A)	6829	329	1082	239
H(39B)	6775	156	1765	239
H(39C)	7186	-118	1449	239
H(39D)	6065	885	1750	239
H(40A)	10848	2861	4258	154
H(40B)	11056	1474	3950	154
H(42)	9882	2282	5152	121
H(43)	8231	1322	5762	135
H(44)	6656	12	5424	135
H(45)	6604	-278	4472	137
H(46)	8223	715	3837	127
H(47A)	5306	2104	1812	331
H(47B)	4705	1365	1311	331
H(47C)	5653	2601	1171	331
H(47D)	6060	2068	748	315
H(47E)	5167	788	882	315
H(47F)	6643	593	579	315

Ellipsoid Plot of **3sa**

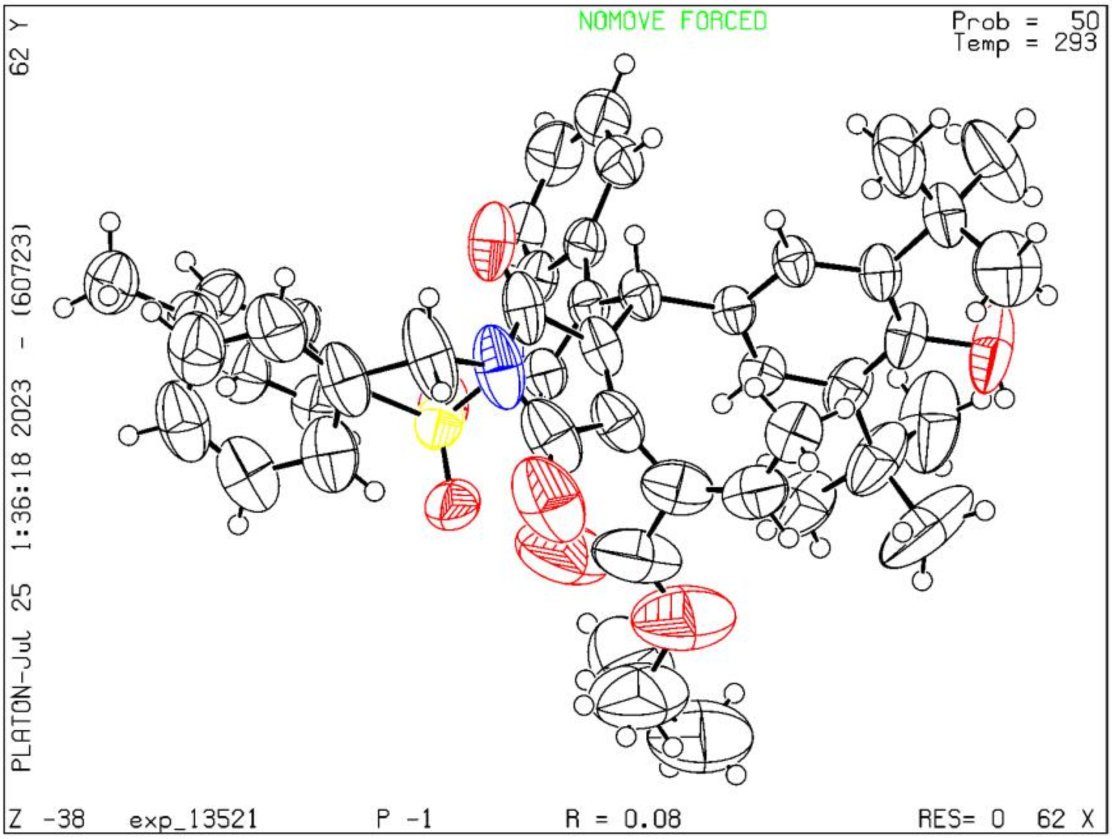

The ^{1}H NMR of **3pa** on the different temperature (20° C, 0° C, -10° C and -25° C)

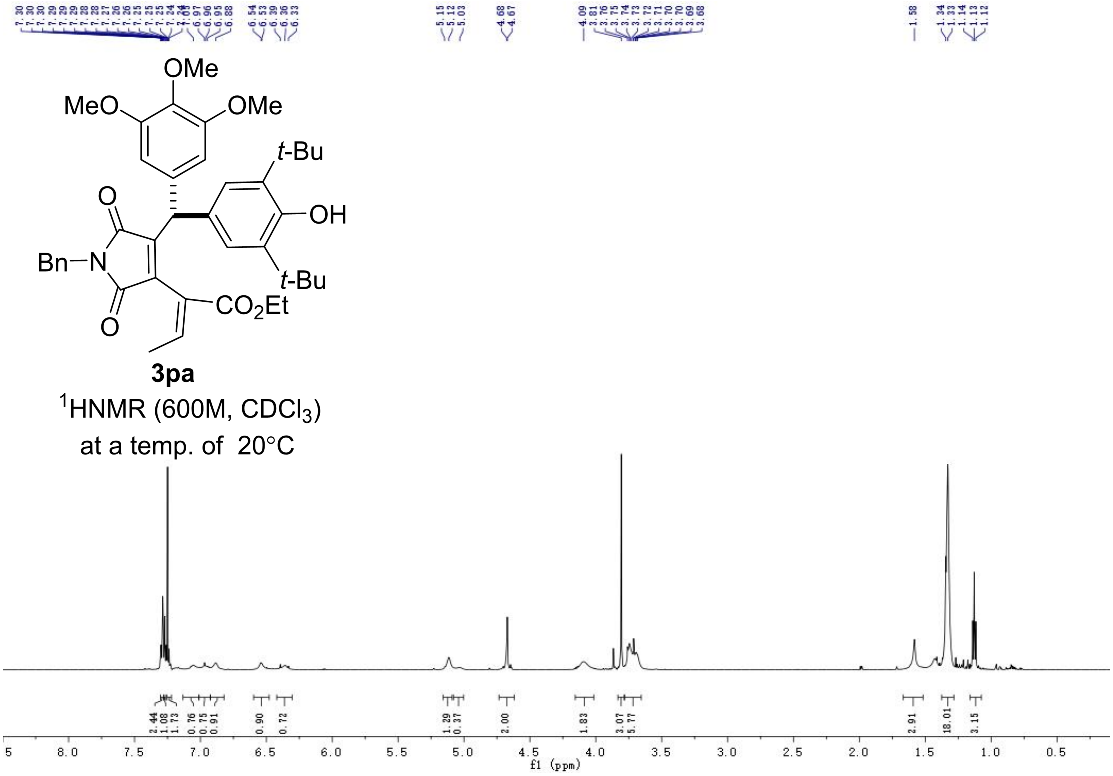

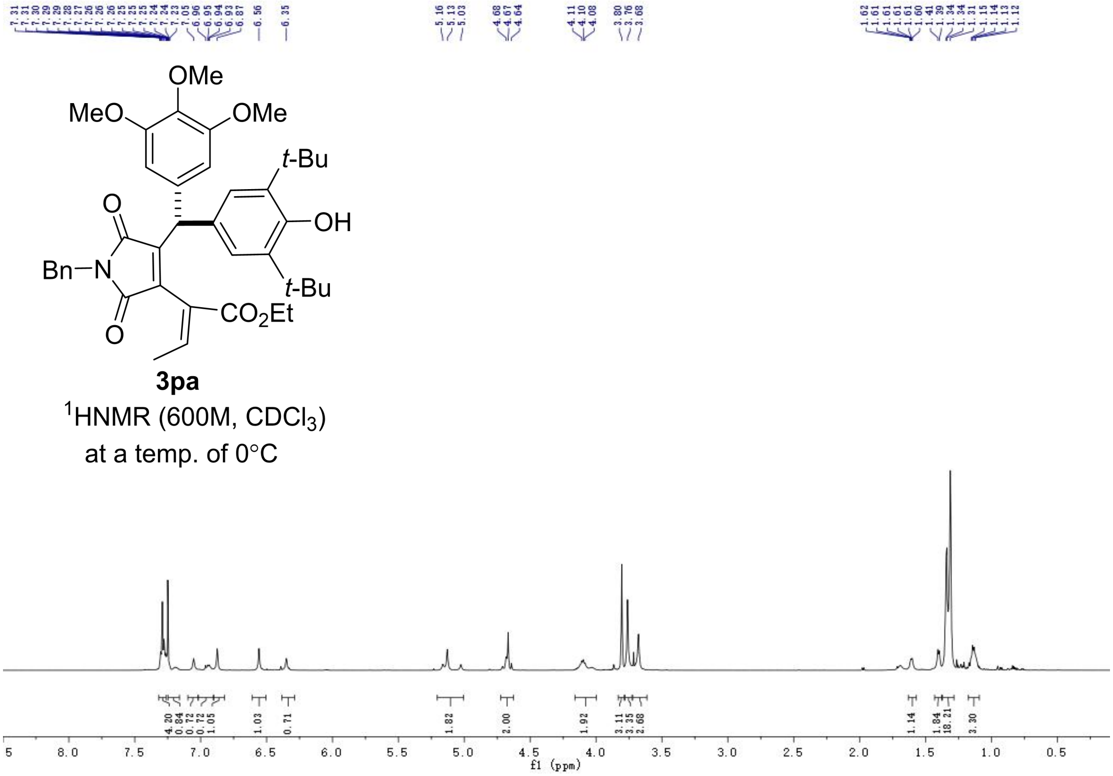

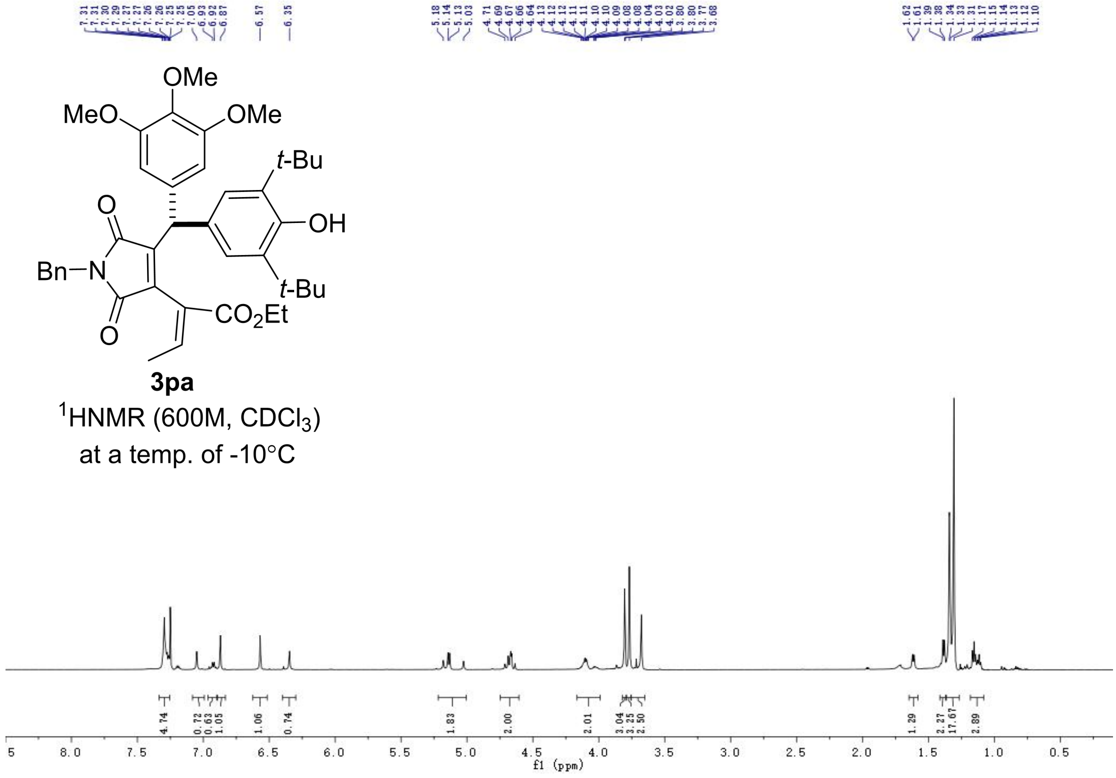
3pa
^{1}HNMR (600M, $CDCl_3$)
at a temp. of -10°C
f1 (ppm)

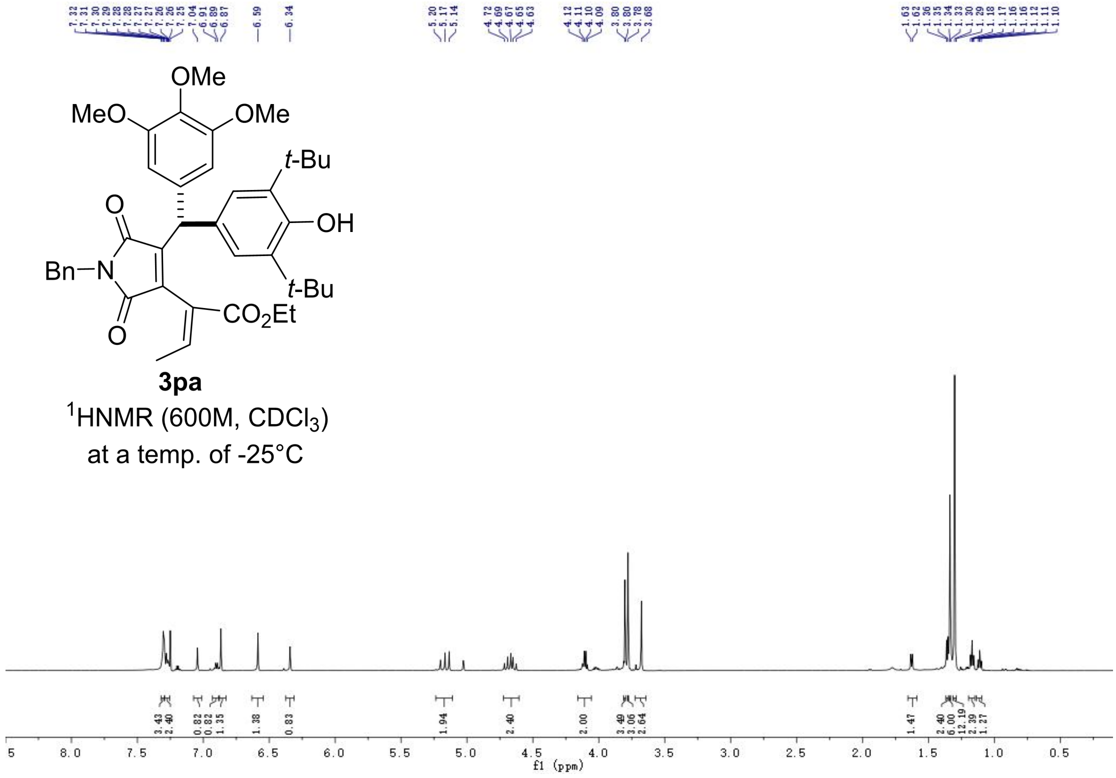
3pa
^{1}HNMR (600M, $CDCl_3$)
at a temp. of -25°C
f1 (ppm)

The ^{13}C NMR of **3pa** on the different temperature (25° C, 0° C, -10° C and -25° C)

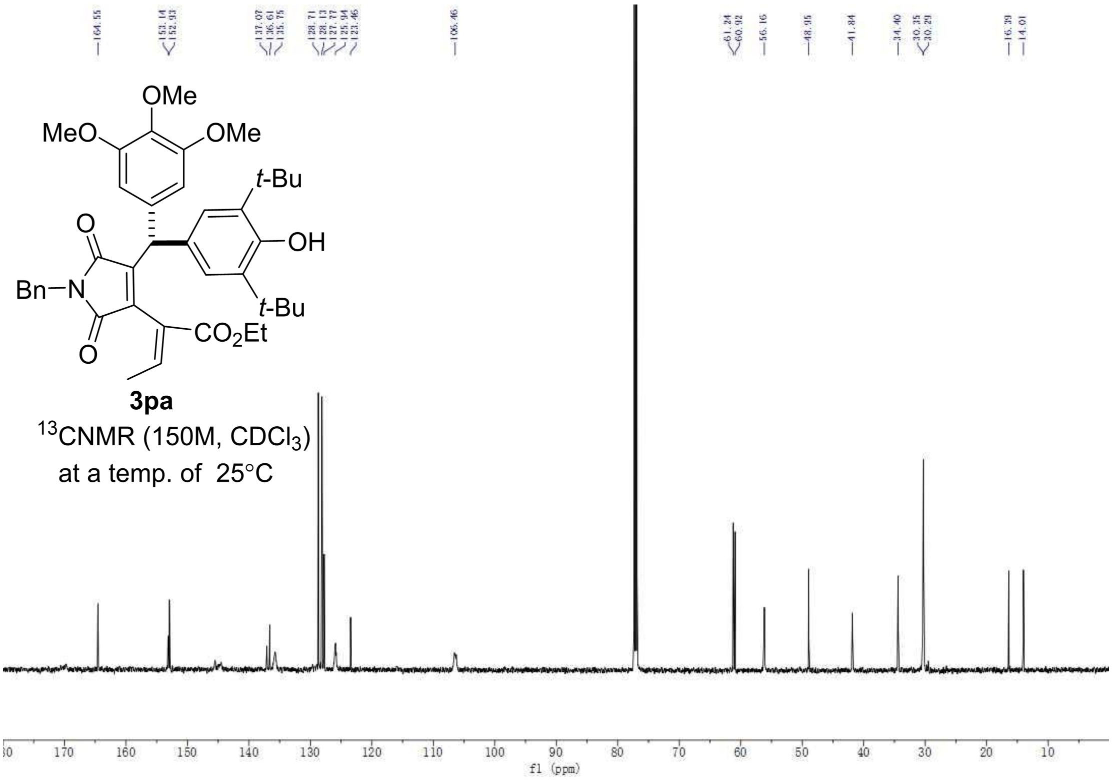

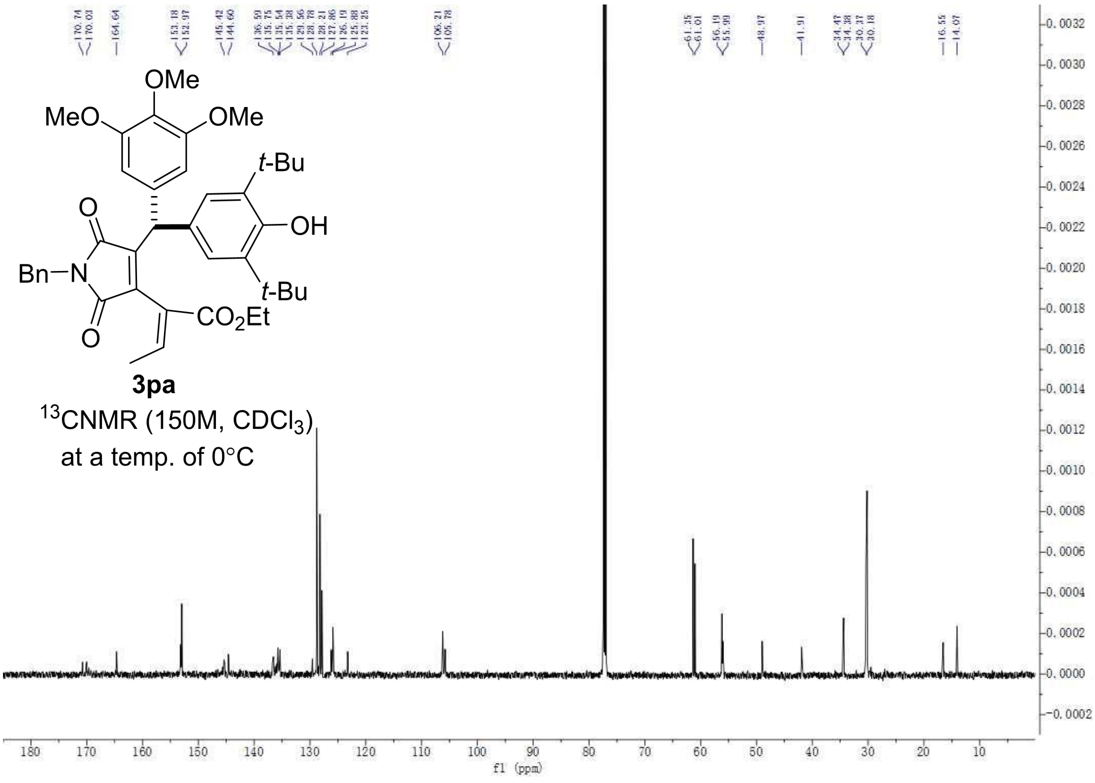

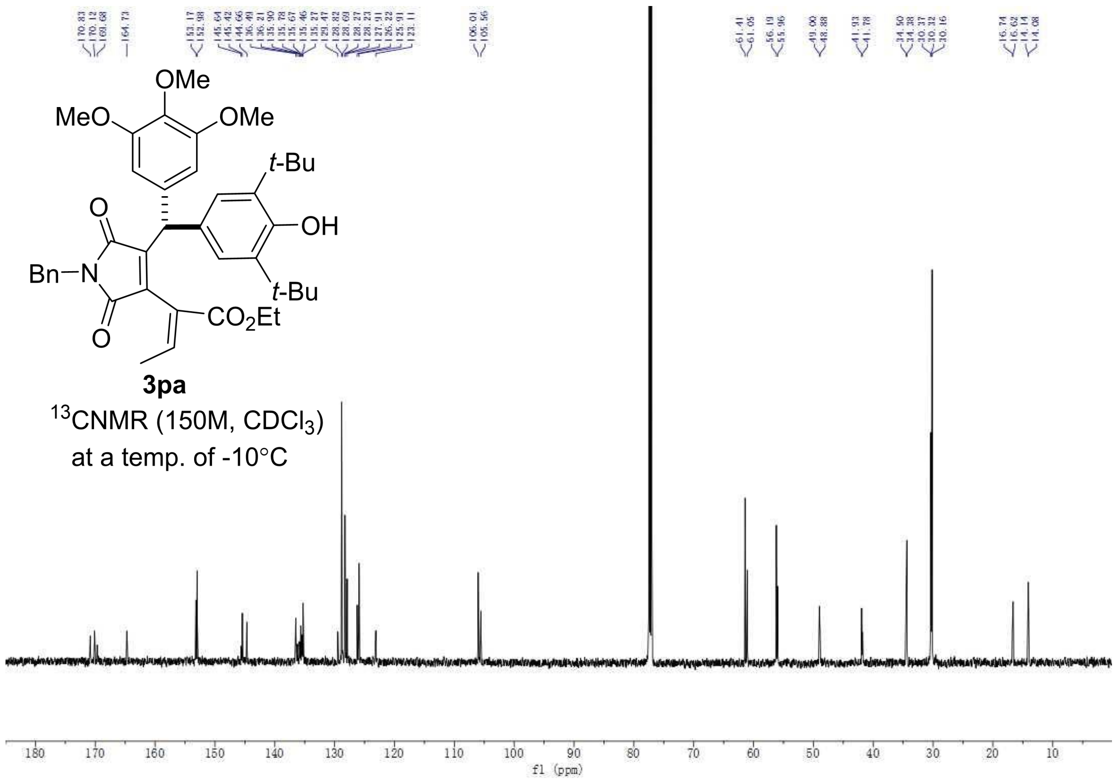
OMe
MeO
OMe
t-Bu
OH
O
Bn—N
t-Bu
O
CO2Et
3pa
13CNMR (150M, CDCl3)
at a temp. of -10°C
f1 (ppm)

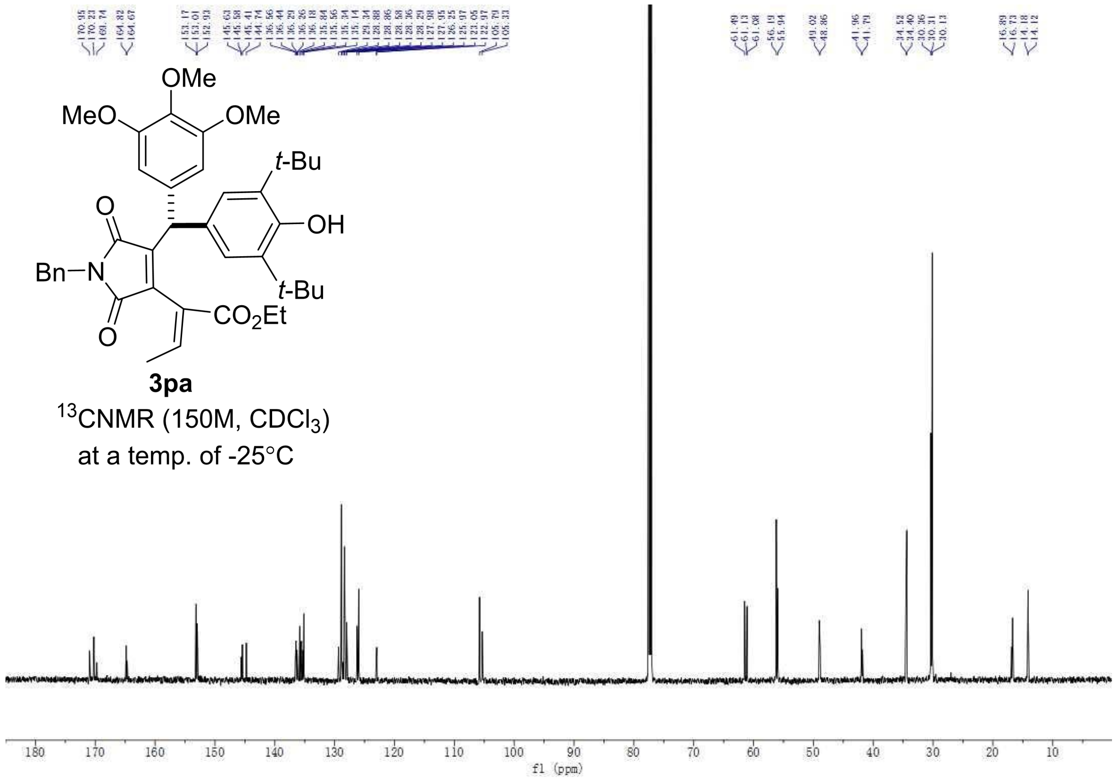
OMe
MeO
OMe
t-Bu
OH
O
Bn—N
t-Bu
O
CO2Et
3pa
13CNMR (150M, CDCl3)
at a temp. of -25°C
f1 (ppm)
